# Supplementary material for: Development of an orally bioavailable CDK12/13 degrader and induction of synthetic lethality with AKT pathway inhibition
Source: Cell Rep Med. 2024 Sep 30;5(10):101752. doi: 10.1016/j.xcrm.2024.101752 (PMC11513842; doi:10.1016/j.xcrm.2024.101752)
Supplement: Document S1. Figures S1–S7, Tables S1, and Method S1 [file mmc1.pdf]

Cell Reports Medicine, Volume *5*

Supplemental information

Development of an orally bioavailable CDK12/13 degrader and induction of synthetic lethality with AKT pathway inhibition

Yu Chang, Xiaoju Wang, Jianzhang Yang, Jean Ching-Yi Tien, Rahul Mannan, Gabriel Cruz, Yuping Zhang, Josh N. Vo, Brian Magnuson, Somnath Mahapatra, Hanbyul Cho, Saravana Mohan Dhanasekaran, Cynthia Wang, Zhen Wang, Licheng Zhou, Kaijie Zhou, Yang Zhou, Pujuan Zhang, Weixue Huang, Lanbo Xiao, Weihuang Raymond Liu, Rudana Hamadeh, Fengyun Su, Rui Wang, Stephanie J. Miner, Xuhong Cao, Yunhui Cheng, Rohit Mehra, Ke Ding, and Arul M. Chinnaiyan

Supplemental information

Development of an orally bioavailable CDK12/13 degrader and induction of synthetic lethality with AKT pathway inhibition

Yu Chang[1,2,8], Xiaoju Wang[1,2,3,8], Jianzhang Yang[4,5,8], Jean Ching-Yi Tien[1,2], Rahul Mannan[1,2], Gabriel Cruz[1,2], Yuping Zhang[1,2], Josh N. Vo[1,2], Brian Magnuson[1,2], Somnath Mahapatra[1,2], Hanbyul Cho[1,2], Saravana Mohan Dhanasekaran[1,2,3], Cynthia Wang[1,2], Zhen Wang[4], Licheng Zhou[4,5], Kaijie Zhou[4], Yang Zhou[5], Pujuan Zhang[4], Weixue Huang[4], Lanbo Xiao[1,2], Weihuang Raymond Liu[1], Rudana Hamadeh[1], Fengyun Su[1,2], Rui Wang[1,2], Stephanie J. Miner[1,2], Xuhong Cao[1,2,3,6], Yunhui Cheng[1,2], Rohit Mehra[1,2,3,7], Ke Ding[4,*], and Arul M. Chinnaiyan[1,2,3,6,7,9,*]

[1] Michigan Center for Translational Pathology, University of Michigan, Ann Arbor, MI, 48109, USA

[2] Department of Pathology, University of Michigan, Ann Arbor, MI, 48109, USA

[3] Rogel Cancer Center, University of Michigan, Ann Arbor, MI, 48109, USA

[4] State Key Laboratory of Chemical Biology, Shanghai Institute of Organic Chemistry, Chinese Academy of Sciences, Shanghai 200032, People's Republic of China

[5] School of Pharmaceutical Sciences, Jinan University, Guangzhou 511436, People's Republic of China

[6] Howard Hughes Medical Institute, University of Michigan, Ann Arbor, MI, 48109, USA

[7] Department of Urology, University of Michigan, Ann Arbor, MI, 48109, USA

[8]These authors contributed equally

[9]Lead contact

[*]Correspondence: arul@umich.edu (A.M.C.) and dingk@sioc.ac.cn (K.D.)

Figure S1

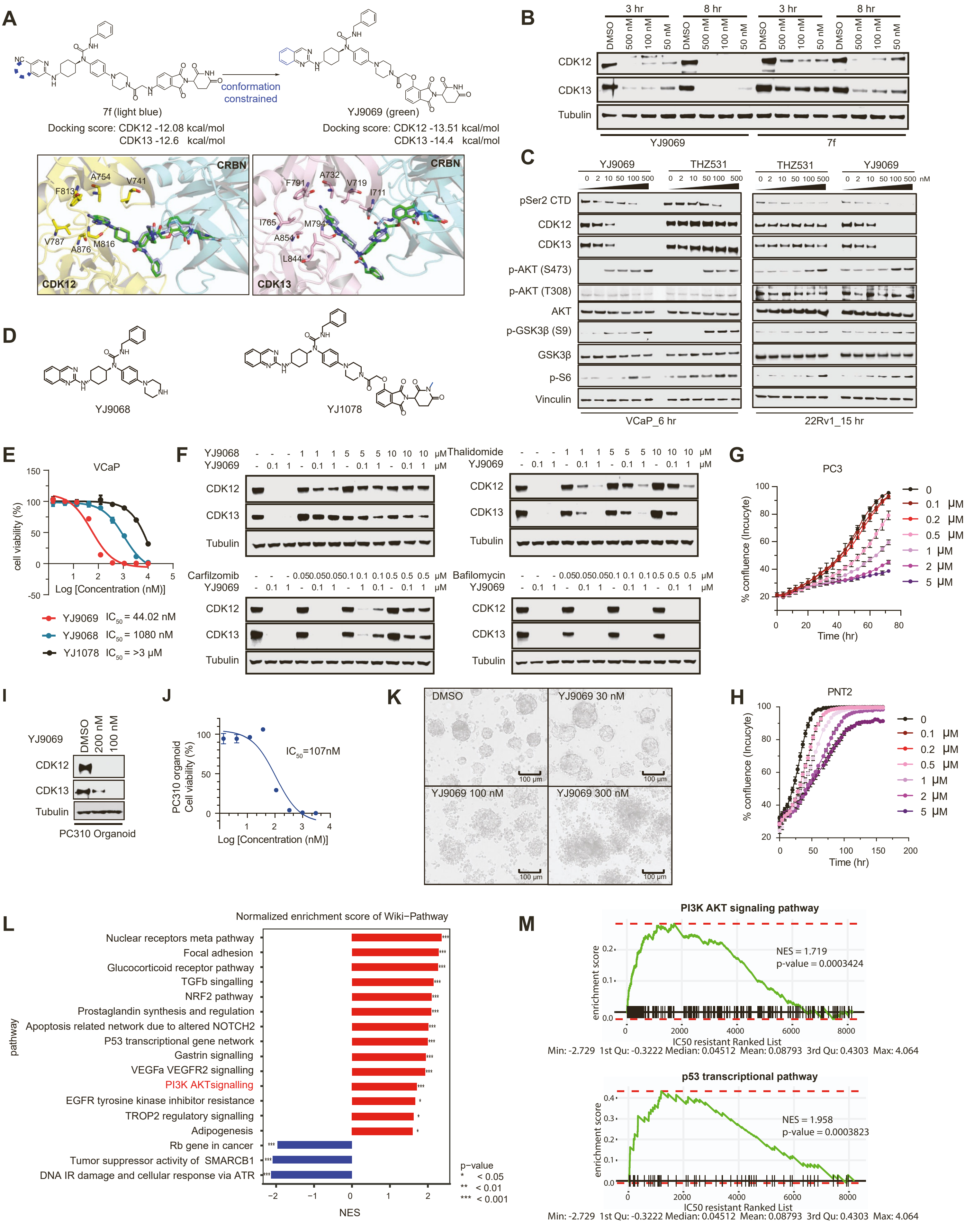

A
7f (light blue)
Docking score: CDK12 -12.08 kcal/mol
CDK13 -12.6 kcal/mol
conformation constrained
YJ9069 (green)
Docking score: CDK12 -13.51 kcal/mol
CDK13 -14.4 kcal/mol
CRBN
CDK12
CDK13
B
YJ9069
7f
C
VCaP_6 hr
22Rv1_15 hr
D
YJ9068
YJ1078
E
VCaP
YJ9069 IC50 = 44.02 nM
YJ9068 IC50 = 1080 nM
YJ1078 IC50 = >3 μM
F
G
PC3
H
PNT2
I
PC310 Organoid
J
IC50=107nM
K
DMSO
YJ9069 30 nM
YJ9069 100 nM
YJ9069 300 nM
L
Normalized enrichment score of Wiki−Pathway
M
PI3K AKT signaling pathway
NES = 1.719
p-value = 0.0003424
p53 transcriptional pathway
NES = 1.958
p-value = 0.0003823

Figure S1. Design and characterization of YJ9069 in multiple cancer cell lines. Related to Figure 1.
(A) Design and modeling of a CDK12/13 degrader YJ9069 derived from 7f (*J Med Chem. 2022, 11066-11083*).
(B) Immunoblots of CDK12 and CDK13 in VCaP cells upon treatment with YJ9069 or 7f for 3 and 8 hours at the indicated concentrations. Tubulin is used as a loading control.
(C) Immunoblots of CDK12 and CDK13 in VCaP and 22Rv1 cells upon treatment with YJ9069 or THZ531 at increasing concentrations. Vinculin is used as a loading control.
(D) Chemical structures of YJ9068 (warhead of YJ9069) and YJ1078 (inactive degrader of YJ9069).
(E) Dose-response curves and IC_{50} of VCaP cells treated with YJ9069, YJ9068, and YJ1078 for 5 days. Data are presented as mean +/− SD from n=3 independent experiments.
(F) Immunoblots of CDK12 and CDK13 in VCaP cells pre-treated with YJ9068, thalidomide, carfilzomib, or bafilomycin for 1 hour, then treated with YJ9069 at noted concentrations for 4 hours. Tubulin is the loading control probed on all immunoblots.
(G-H) Growth curves of PC3 and PNT2 cells upon treatment with increasing concentrations of YJ9069. Data are presented as mean +/− SD from n=3 independent experiments.
(I) Immunoblots of CDK12 and CDK13 levels upon treatment of PC310 organoids with YJ9069 at 100 nM and 200 nM for 15 hours. Tubulin is used as a loading control.
(J) Dose-response curves and IC_{50} of PC310 organoids treated with YJ9069. Data are presented as mean +/− SD from n=3 independent experiments.
(K) Images of PC310 organoids after treatment with vehicle or YJ9069 at 30, 100, and 300 nM for 5 days (scale=100 μm).
(L-M) Gene Set Enrichment Analysis (GSEA) of cell viability in 124 cell lines (Fig. 1G). NES, normalized enrichment score. RNA-sequencing data from 124 cell lines were obtained from the Cancer Cell Line Encyclopedia (CCLE). Cell lines were categorized based on IC_{50} values from Figure 1G, with 30 annotated as “Sensitive” (< 500 nM) and the remaining 94 as “Resistant” (≥ 500 nM). Gene rankings were based on log fold change (logFC), and enrichment analysis was performed against curated pathways from the Molecular Signatures Database (MSigDB 3.0).

Figure S2

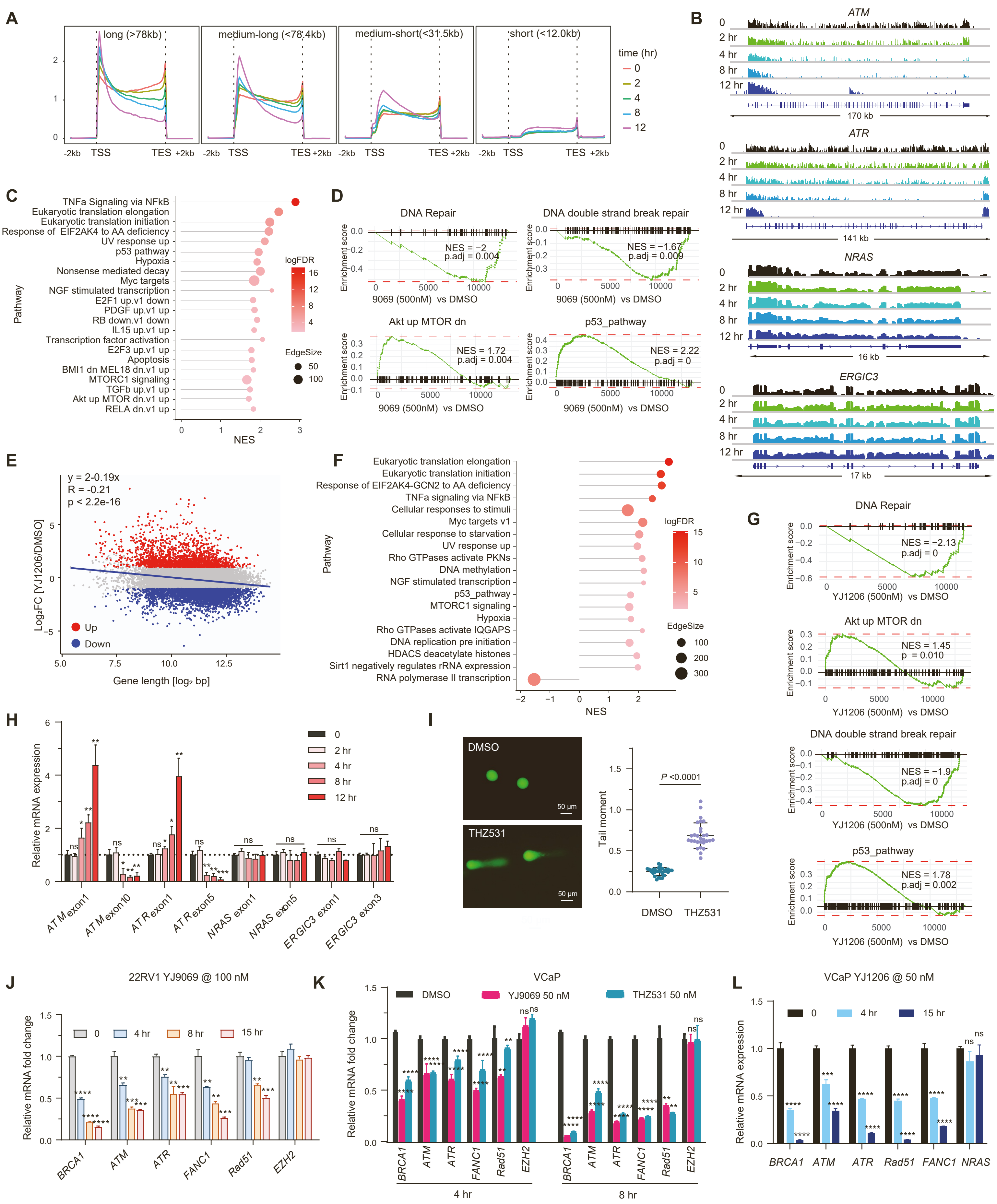

A
long (>78kb)
medium-long (<78.4kb)
medium-short(<31.5kb)
short (<12.0kb)
time (hr)
0
2
4
8
12
-2kb TSS TES +2kb
B
ATM
170 kb
ATR
141 kb
NRAS
16 kb
ERGIC3
17 kb
C
TNFa Signaling via NFkB
Eukaryotic translation elongation
Eukaryotic translation initiation
Response of EIF2AK4 to AA deficiency
UV response up
p53 pathway
Hypoxia
Nonsense mediated decay
Myc targets
NGF stimulated transcription
E2F1 up.v1 down
PDGF up.v1 up
RB down.v1 down
IL15 up.v1 up
Transcription factor activation
E2F3 up.v1 up
Apoptosis
BMI1 dn MEL18 dn.v1 up
MTORC1 signaling
TGFb up.v1 up
Akt up MTOR dn.v1 up
RELA dn.v1 up
Pathway
logFDR
EdgeSize
NES
D
DNA Repair
NES = −2
p.adj = 0.004
DNA double strand break repair
NES = −1.67
p.adj = 0.009
Akt up MTOR dn
NES = 1.72
p.adj = 0.004
p53_pathway
NES = 2.22
p.adj = 0
Enrichment score
9069 (500nM) vs DMSO
E
y = 2-0.19x
R = -0.21
p < 2.2e-16
Log₂FC [YJ1206/DMSO]
Up
Down
Gene length [log₂ bp]
F
Eukaryotic translation elongation
Eukaryotic translation initiation
Response of EIF2AK4-GCN2 to AA deficiency
TNFa signaling via NFkB
Cellular responses to stimuli
Myc targets v1
Cellular response to starvation
UV response up
Rho GTPases activate PKNs
DNA methylation
NGF stimulated transcription
p53_pathway
MTORC1 signaling
Hypoxia
Rho GTPases activate IQGAPS
DNA replication pre initiation
HDACS deacetylate histones
Sirt1 negatively regulates rRNA expression
RNA polymerase II transcription
Pathway
logFDR
EdgeSize
NES
G
DNA Repair
NES = −2.13
p.adj = 0
Akt up MTOR dn
NES = 1.45
p = 0.010
DNA double strand break repair
NES = −1.9
p.adj = 0
p53_pathway
NES = 1.78
p.adj = 0.002
YJ1206 (500nM) vs DMSO
H
Relative mRNA expression
0
2 hr
4 hr
8 hr
12 hr
ATM exon1
ATM exon10
ATR exon1
ATR exon5
NRAS exon1
NRAS exon5
ERGIC3 exon1
ERGIC3 exon3
I
DMSO
THZ531
50 μm
Tail moment
P <0.0001
J
22RV1 YJ9069 @ 100 nM
Relative mRNA fold change
0
4 hr
8 hr
15 hr
BRCA1
ATM
ATR
FANC1
Rad51
EZH2
K
VCaP
DMSO
YJ9069 50 nM
THZ531 50 nM
Relative mRNA fold change
BRCA1
ATM
ATR
FANC1
Rad51
EZH2
4 hr
8 hr
L
VCaP YJ1206 @ 50 nM
Relative mRNA expression
0
4 hr
15 hr
BRCA1
ATM
ATR
Rad51
FANC1
NRAS

Figure S2. DNA repair and DNA double strand break repair pathways are downregulated upon CDK12/13 degradation. Related to Figure 2.
(A) Average metagene profiles for protein-coding genes stratified according to quartiles of gene length distribution in VCaP cells treated with YJ9069 (500 nM) at different time points.
(B) Sequence tracks of nascent transcript expression for long genes (top panel, *ATM* and *ATR*) and short genes (bottom panel, *NRAS* and *ERGIC3*) in VCaP cells treated with YJ9069 at different time points.
(C) Top KEGG pathway analyses of RNA-seq in VCaP cells upon CDK12/13 degradation by YJ9069 (500 nM) for 12 hours.
(D) Gene set enrichment analysis (GSEA) plots for the top rank-ordered dysregulated gene pathways in VCaP cells after YJ9069 treatment. NES, normalized enrichment score; p. adj, adjusted p-value.
(E) Scatter plot showing Log_2 fold changes in gene expression vs. Log_2 scale in gene length for each protein-coding gene in VCaP cells following treatment with YJ1206 at 500 nM for 12 hours ($p < 2.2e{-16}$, F-test). Differentially expressed genes are indicated ($FDR < 0.05$ and $Log_2 FC > 1$).
(F) Top KEGG pathway analyses of RNA-seq in VCaP cells upon CDK12/13 degradation by YJ1206 (500 nM) for 12 hours.
(G) Gene set enrichment analysis (GSEA) plots for the top rank-ordered dysregulated gene pathways in VCaP cells after YJ1206 treatment.
(H) Analysis of indicated gene expression by qPCR for nascent RNA after treatment with YJ1206 at different time points. Data are presented as mean +/− SD from n=3 independent experiments.
(I) Representative images of comet assay in VCaP cells after treatment with vehicle or THZ531 (100 nM) for 12 hours (scale=50 μm) (left panel) and quantification of tail moments (right panel). Boxplots represent interquartile ranges; horizontal bars denote the median. For each condition, 30 cells were analyzed.
(J-L) Analysis of indicated gene expression by qPCR at 4 hours, 8 hours, and/or 15 hours (as indicated in the figure) with YJ9069, THZ531, or YJ1206 in 22Rv1 or VCaP cells. Data are presented as mean +/− SD from n=3 independent experiments per condition.

Figure S3

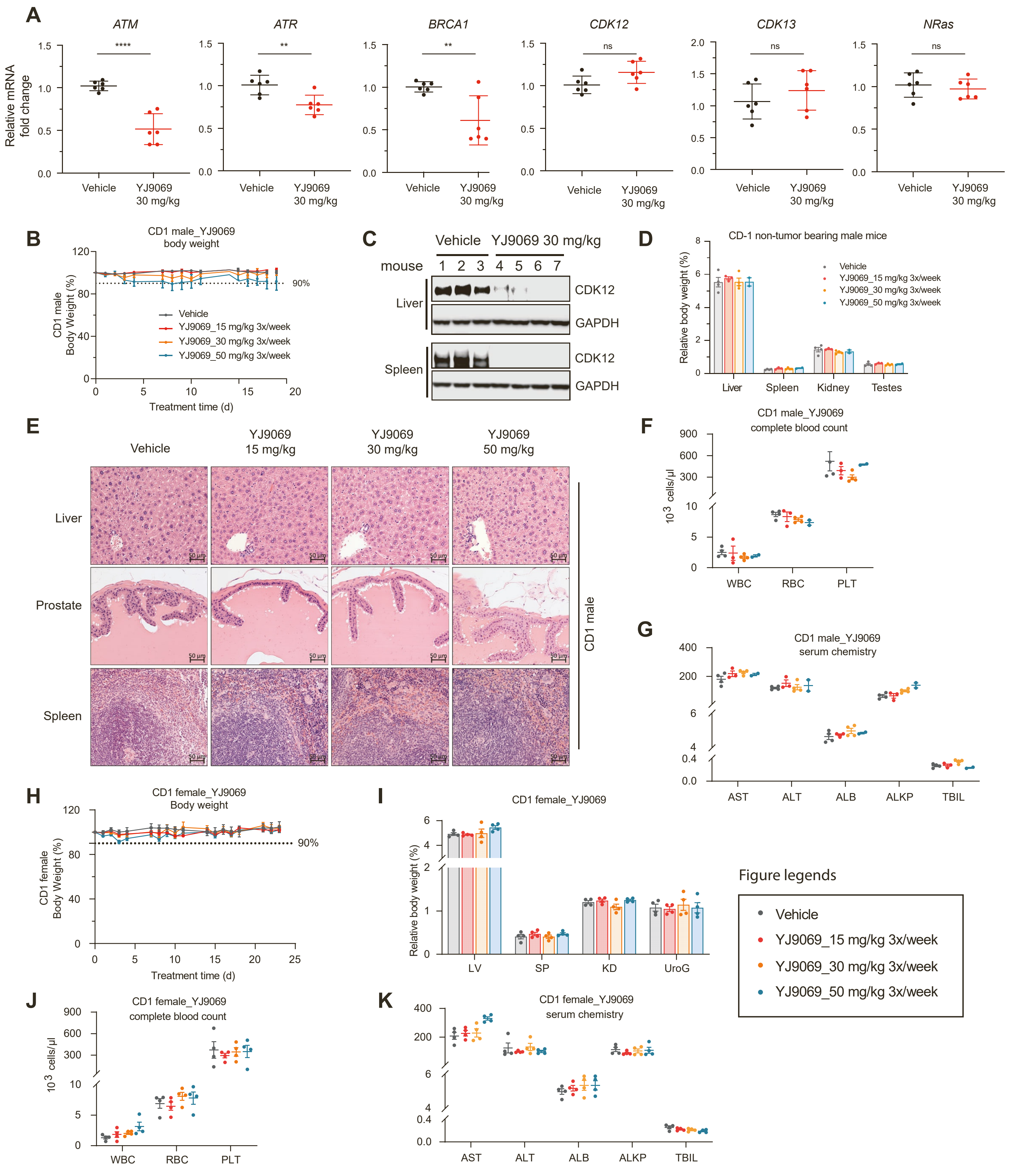

A
ATM
ATR
BRCA1
CDK12
CDK13
NRas
Relative mRNA fold change
****
**
**
ns
ns
ns
Vehicle
YJ9069 30 mg/kg
B
CD1 male_YJ9069 body weight
CD1 male Body Weight (%)
90%
Vehicle
YJ9069_15 mg/kg 3x/week
YJ9069_30 mg/kg 3x/week
YJ9069_50 mg/kg 3x/week
Treatment time (d)
C
Vehicle
YJ9069 30 mg/kg
mouse
1 2 3 4 5 6 7
Liver
Spleen
CDK12
GAPDH
D
CD-1 non-tumor bearing male mice
Relative body weight (%)
Liver
Spleen
Kidney
Testes
E
Vehicle
YJ9069 15 mg/kg
YJ9069 30 mg/kg
YJ9069 50 mg/kg
Liver
Prostate
Spleen
CD1 male
50 μm
F
CD1 male_YJ9069 complete blood count
10^3 cells/μl
WBC
RBC
PLT
G
CD1 male_YJ9069 serum chemistry
AST
ALT
ALB
ALKP
TBIL
H
CD1 female_YJ9069 Body weight
CD1 female Body Weight (%)
90%
Treatment time (d)
I
CD1 female_YJ9069
Relative body weight (%)
LV
SP
KD
UroG
J
CD1 female_YJ9069 complete blood count
10^3 cells/μl
WBC
RBC
PLT
K
CD1 female_YJ9069 serum chemistry
AST
ALT
ALB
ALKP
TBIL
Figure legends
Vehicle
YJ9069_15 mg/kg 3x/week
YJ9069_30 mg/kg 3x/week
YJ9069_50 mg/kg 3x/week

Figure S3. YJ9069 demonstrates mild toxicity in CD1 male immune-competent mice. Related to Figure 3.
(A) Expression of indicated genes (qPCR) in tumors after treatment with YJ9069 (i.v., 30 mg/kg, 3x/week) for 5 days in castrated VCaP xenografts. Data are presented as mean values ± SD of triplicate points (n = 6 per condition). $*p < 0.05$, $**p < 0.01$, $***p < 0.001$, $****p < 0.0001$ by t-test.
(B) Body weight percentage of the vehicle and YJ9069 groups at noted doses in CD-1 male mice throughout the treatment period (two-sided t-test). Data are presented as mean ± SEM (n = 4, biological replicates).
(C) Immunoblot of CDK12 from host organs in the CD1 male mouse after 5 days treatment with YJ9069 (i.v., 30 mg/kg, 3x/week). GAPDH is the loading control.
(D) Major organ weight measurement for vehicle and YJ9069 groups at noted doses in CD-1 male mouse. Data are presented as mean ± SEM (n = 4, biological replicates).
(E) Representative H&E staining for liver, spleen, and prostate from different groups of the CD-1 male toxicity study (scale=50 μm).
(F-G) Complete blood counts (F) and serum chemistry (G) for the vehicle and YJ9069 groups at noted doses in CD-1 male mice. WBC, white blood cells; RBC, red blood cells; PLT, platelets. Data are presented as mean ± SD (n = 4, biological replicates).
(H-K) is the same as Fig. S3B, S3D, and S3F-G, except in CD-1 female mice (n = 4 per condition).

Figure S4

A

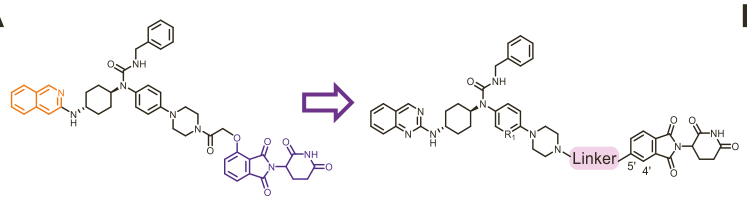

B

Compds	Linker + E3 ligand	R_1	VCaP IC_{50} (nM)
YJ9069	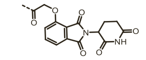	CH	22.90
YJ1090	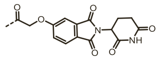	CH	93.85
YJ1094	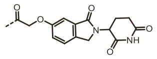	CH	25.21
YJ1114	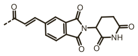	CH	2025
YJ1130	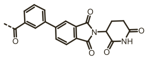	CH	125.5
YJ1096	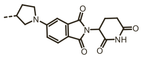	CH	97.75
YJ1105	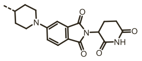	CH	15.11
YJ1205	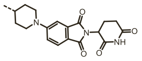	N	3.93
YJ1206	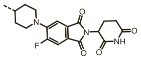	N	13.50

C

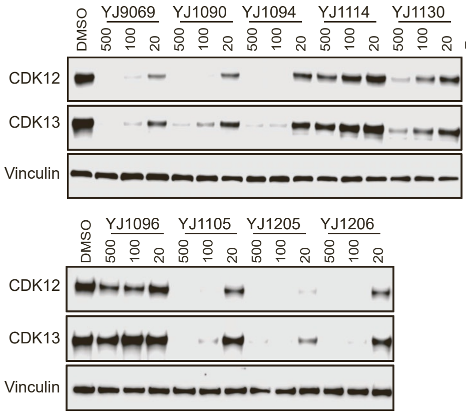

D

Parameter	YJ9069 (mg/kg)		YJ1105		YJ1205		YJ1206	
	i.v. (2.5)	Oral (10)	i.v. (2.5)	Oral (10)	i.v. (2.5)	Oral (10)	i.v. (2.5)	Oral (10)
$T_{1/2}$ (h)	4.39	NA	7.05	7.2	3.81	4.2	5.1 5	6. 24
T_{max} (h)	0.08	1.56	0.08	6	0.08	4.7	0.08	6.0
C_{max} (μM)	4.13	0.006	1.58	0.715	4.14	0.66	3.49	1.26
AUC(0 -t)(h *ng/mL)	2929.22	5.06	8039.69	8567.43	10519.6	6679.0	9463.33	14824.81
AUC(0 -∞)(h*ng/mL)	2968.30	NA	87 41 .34	9805.4	10615.68	6842.8	9763.68	16205.27
CL(mL/h/kg)	848.10	NA	288.16	NA	235.84	NA	257.41	NA
F(%)		0.04%		26.64%		15.87	39.16%	

E

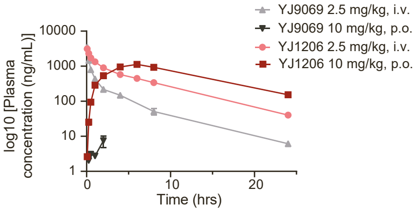

F

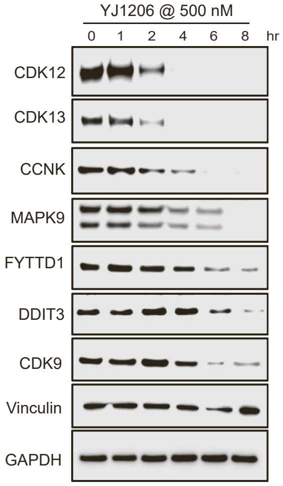

G

Gene name	Log₂(fold change: YJ1206/DMSO)	*P*-value
CDK12	-3.18	1E-17
CDK13	-3.17	1E-17
CDK1	-0.14	0.0070945
CDK2	0.02	0.7635315
CDK4	-0.25	0.0096923
CDK5	0.03	0.702421
CDK6	0.12	0.1139111
CDK7	-0.18	0.286255
CDK8	0.04	0.6896741
CDK9	-1.63	1E-17
CDK10	-0.18	0.3721337
CDK14	-0.16	0.5010827
CDK16	0.1	0.2626574
CDK17	-0.22	0.1593374
CDK18	0.01	0.8142111

H

Gene name	Log₂(fold change: YJ1206/DMSO)	*P*-value
CDK12	-3.18	1E-17
CDK13	-3.17	1E-17
FYTTD1	-2.96	1E-17
DDIT3	-2.78	1E-17
MAPK9	-2.29	1E-17
CCNK	-2.28	1E-17
FOS	3.62	1E-17
JUNB	2.48	1E-17

I

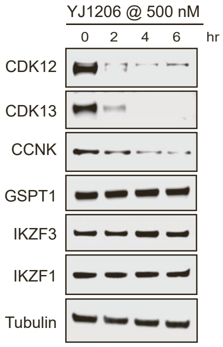

Figure S4. An oral available degrader, YJ1206, is developed and optimized with high specificity towards CDK12/13. Related to Figure 4.
(A-B) Chemical optimization of the linker from YJ9069 to oral CDK12/13 degraders (IC_{50} values are calculated from n = 3 independent experiments).
(C) Immunoblots of CDK12 and CDK13 in VCaP cells treated with synthesized CDK12/13 degraders for 4 hours.
(D) Pharmacokinetic profiles of YJ9069, YJ1105, YJ1205, and YJ1206 following intravenous (i.v., 2.5 mg/kg) and oral (p.o., 10 mg/kg) injection in Sprague Dawley (SD) rats.
(E) Plasma concentration time curve of YJ1206 and YJ9069 with intravenous (i.v., 2.5 mg/kg) and oral (p.o., 10 mg/kg) injection in SD rats (n = 3 per condition).
(F) Immunoblots of the down-regulated proteins shown in Fig. S4H in 22Rv1 cells treated with YJ1206 at different time points.
(G) Proteomic analysis of CDK family proteins in 22Rv1 cells treated with YJ1206 (500 nM) versus DMSO.
(H) The top up- and down-regulated proteins from the global proteomic profile study.
(I) Immunoblots of CDK12, CDK13, CCNK, and neo-substrates in 22Rv1 cells treated with YJ1206 (500 nM) at increasing time durations. Tubulin is used as a loading control.

Figure S5

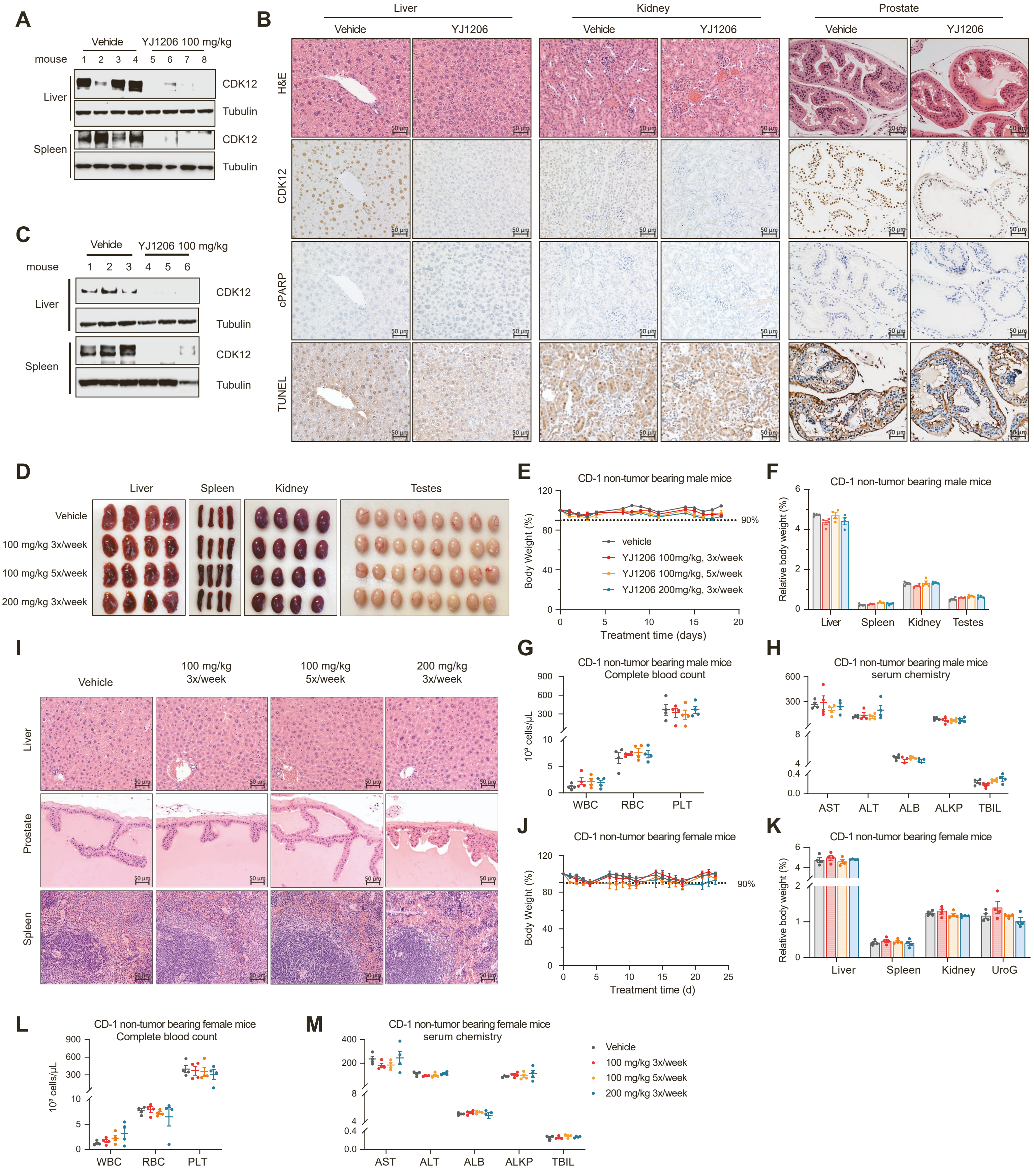
A
Vehicle
YJ1206 100 mg/kg
mouse 1 2 3 4 5 6 7 8
Liver
Spleen
CDK12
Tubulin
B
Liver
Kidney
Prostate
Vehicle
YJ1206
H&E
CDK12
cPARP
TUNEL
50 μm
C
Vehicle
YJ1206 100 mg/kg
mouse 1 2 3 4 5 6
D
Liver
Spleen
Kidney
Testes
Vehicle
100 mg/kg 3x/week
100 mg/kg 5x/week
200 mg/kg 3x/week
E
CD-1 non-tumor bearing male mice
Body Weight (%)
90%
vehicle
YJ1206 100mg/kg, 3x/week
YJ1206 100mg/kg, 5x/week
YJ1206 200mg/kg, 3x/week
Treatment time (days)
F
CD-1 non-tumor bearing male mice
Relative body weight (%)
Liver
Spleen
Kidney
Testes
G
CD-1 non-tumor bearing male mice
Complete blood count
10³ cells/μL
WBC
RBC
PLT
H
CD-1 non-tumor bearing male mice
serum chemistry
AST
ALT
ALB
ALKP
TBIL
I
Vehicle
100 mg/kg 3x/week
100 mg/kg 5x/week
200 mg/kg 3x/week
Liver
Prostate
Spleen
J
CD-1 non-tumor bearing female mice
Body Weight (%)
90%
Treatment time (d)
K
CD-1 non-tumor bearing female mice
Relative body weight (%)
Liver
Spleen
Kidney
UroG
L
CD-1 non-tumor bearing female mice
Complete blood count
10³ cells/μL
WBC
RBC
PLT
M
CD-1 non-tumor bearing female mice
serum chemistry
AST
ALT
ALB
ALKP
TBIL
Vehicle
100 mg/kg 3x/week
100 mg/kg 5x/week
200 mg/kg 3x/week

Figure S5. YJ1206 shows no evidence of toxicity in both male and female immuno-competent CD1 mice. Related to Figure 4.

(A) Immunoblot of CDK12 from host organs in the VCaP-CRPC xenograft model after 5 days treatment with YJ1206 (p.o., 100 mg/kg, 3x/week). Tubulin is the loading control.
(B) Representative H&E staining and immunohistochemistry of CDK12, cleaved PARP, and TUNEL for host organs (scale=50 μm).
(C) Immunoblot of CDK12 from host organs in CD1 male model after 5 days treatment with YJ1206 (p.o., 100 mg/kg, 3x/week). Tubulin is the loading control.
(D) Images for liver, spleen, kidney, and testes from CD1 male model treated with YJ1206 at different doses.
(E) Percent body weight of the vehicle and YJ1206 groups from CD1 male mice throughout the treatment period. Data are presented as mean ± SEM (n = 4 per condition).
(F) Major organ weight of the vehicle and YJ1206 groups at noted doses in CD-1 male mouse. Data are presented as mean ± SEM (n = 4, biological replicates).
(G-H) Complete blood counts and serum chemistry of the vehicle and YJ1206 groups at noted doses in CD-1 male mice. WBC, white blood cells; RBC, red blood cells; PLT, platelets. Data are presented as mean ± SD (n = 4, biological replicates).
(I) Representative H&E staining for liver, spleen, and prostate of the CD-1 male toxicity study of YJ1206 (scale=50 μm).
(J-M) same as in panels E-H, except in CD1 female mice (n = 4 per condition).

Figure S6

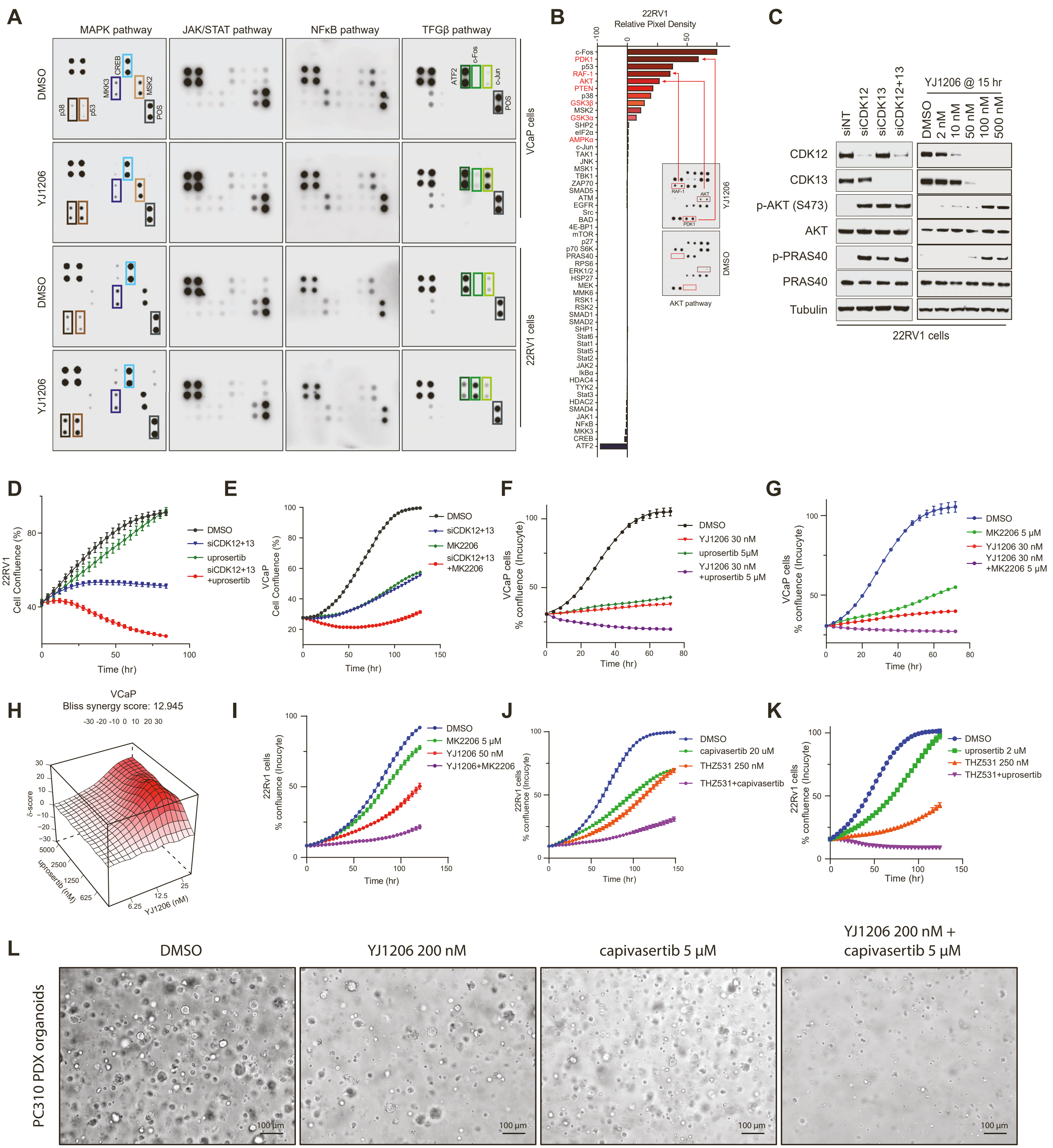

A
MAPK pathway
JAK/STAT pathway
NFκB pathway
TFGβ pathway
DMSO
YJ1206
VCaP cells
22RV1 cells
p38
p53
MKK3
CREB
MSK2
POS
ATF2
c-Fos
c-Jun
B
22RV1
Relative Pixel Density
AKT pathway
C
siNT
siCDK12
siCDK13
siCDK12+13
YJ1206 @ 15 hr
DMSO
2 nM
10 nM
50 nM
100 nM
500 nM
CDK12
CDK13
p-AKT (S473)
AKT
p-PRAS40
PRAS40
Tubulin
22RV1 cells
D
22RV1 Cell Confluence (%)
Time (hr)
DMSO
siCDK12+13
uprosertib
siCDK12+13 +uprosertib
E
VCaP Cell Confluence (%)
DMSO
siCDK12+13
MK2206
siCDK12+13 +MK2206
F
VCaP cells % confluence (Incucyte)
DMSO
YJ1206 30 nM
uprosertib 5μM
YJ1206 30 nM +uprosertib 5 μM
G
DMSO
MK2206 5 μM
YJ1206 30 nM
YJ1206 30 nM +MK2206 5 μM
H
VCaP
Bliss synergy score: 12.945
δ-score
uprosertib (nM)
YJ1206 (nM)
I
22Rv1 cells % confluence (Incucyte)
DMSO
MK2206 5 μM
YJ1206 50 nM
YJ1206+MK2206
J
DMSO
capivasertib 20 uM
THZ531 250 nM
THZ531+capivasertib
K
DMSO
uprosertib 2 uM
THZ531 250 nM
THZ531+uprosertib
L
DMSO
YJ1206 200 nM
capivasertib 5 μM
YJ1206 200 nM + capivasertib 5 μM
PC310 PDX organoids
100 μm

Figure S6. YJ1206 induces phosphorylation of AKT and exhibits synthetic lethality with AKT inhibitors *in vitro*. Related to Figure 5.

(A) Representative blots of human phosphorylation pathway profiling array for MAPK, JAK/STAT, NFκB, and TGFβ pathways in VCaP or 22Rv1 cells treated with or without YJ1206 for 15 hours at a concentration of 500 nM.

(B) Human phosphorylation pathway profiling array analysis of 22Rv1 cells treated with YJ1206 (500 nM) for 15 hours.

(C) Immunoblot of the noted proteins in 22Rv1 cells treated with YJ1206 at increasing concentrations, or with siRNA targeting CDK12 and/or CDK13. Tubulin is the loading control.

(D-E) Real-time growth curves of 22Rv1 or VCaP cells upon treatment with siCDK12/13 and/or AKT inhibitor, uprosertib or MK2206. Data are presented as mean +/− SD from n = 3 independent experiments.

(F-G) Real-time growth curves of VCaP cells upon treatment with YJ1206 and/or uprosertib or MK2206. Data are presented as mean +/− SD from n = 3 independent experiments.

(H) VCaP cells were treated with YJ1206 and/or uprosertib at varying concentrations to determine the effect on cell growth and drug synergism, with assessments using the Bliss method. Red peaks in the 3D plots denote synergy, and the average synergy score is noted above the plot.

(I-K) Real-time growth curves of 22Rv1 cells upon treatment with YJ1206 or THZ531 and/or AKT inhibitors, uprosertib, MK2206, or capivasertib. Data are presented as mean +/− SD from n = 3 independent experiments.

(L) Images of PC310 PDX organoids after treatment with YJ1206 and/or capivasertib for 5 days (scale=50 μm).

Figure S7

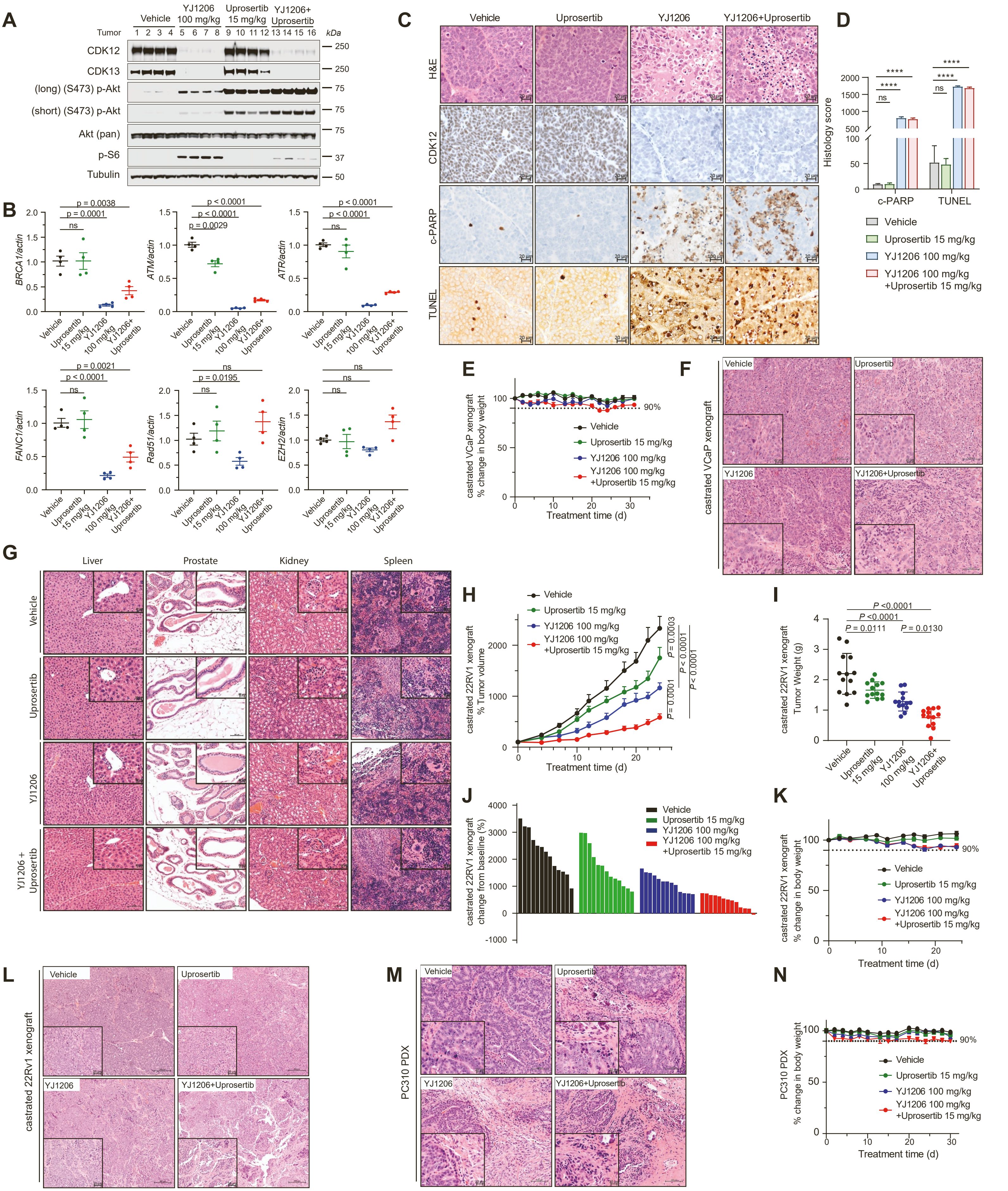
A
Vehicle
YJ1206 100 mg/kg
Uprosertib 15 mg/kg
YJ1206+ Uprosertib
Tumor 1 2 3 4 5 6 7 8 9 10 11 12 13 14 15 16
kDa
CDK12
CDK13
(long) (S473) p-Akt
(short) (S473) p-Akt
Akt (pan)
p-S6
Tubulin
250
250
75
75
75
37
50
B
BRCA1/actin
ATM/actin
ATR/actin
FANC1/actin
Rad51/actin
EZH2/actin
p = 0.0038
p = 0.0001
p = 0.0029
p < 0.0001
p = 0.0021
p = 0.0195
ns
Vehicle
Uprosertib 15 mg/kg
YJ1206 100 mg/kg
YJ1206+ Uprosertib
C
Vehicle
Uprosertib
YJ1206
YJ1206+Uprosertib
H&E
CDK12
c-PARP
TUNEL
D
Histology score
c-PARP
TUNEL
****
ns
Vehicle
Uprosertib 15 mg/kg
YJ1206 100 mg/kg
YJ1206 100 mg/kg +Uprosertib 15 mg/kg
E
castrated VCaP xenograft % change in body weight
90%
Treatment time (d)
F
castrated VCaP xenograft
G
Liver
Prostate
Kidney
Spleen
YJ1206 + Uprosertib
H
castrated 22RV1 xenograft % Tumor volume
P = 0.0003
P < 0.0001
P = 0.0001
I
castrated 22RV1 xenograft Tumor Weight (g)
P <0.0001
P = 0.0111
P = 0.0130
J
castrated 22RV1 xenograft change from baseline (%)
K
castrated 22RV1 xenograft % change in body weight
L
castrated 22Rv1 xenograft
M
PC310 PDX
N
PC310 PDX % change in body weight

Figure S7. The combinatorial regimen of CDK12/13 degraders with AKT inhibitors suppresses tumor growth *in vivo.* Related to Figure 6.
(A) Immunoblot of the noted proteins from castrated VCaP xenograft tumors after 5 days of treatment with YJ1206 (p.o., 100 mg/kg, 3x/week). Tubulin is the loading control.
(B) Expression of the indicated gene by qPCR in tumors from panel A. Data are presented as mean values ± SD (n = 4 per condition). $*p \leq 0.05$, $**p \leq 0.01$, $***p \leq 0.001$, $****p \leq 0.0001$ by t test.
(C) Representative H&E staining and immunohistochemistry of CDK12, cleaved PARP, and TUNEL for the VCaP CRPC xenograft tumors (scale=20 μm).
(D) Histology score for immunohistochemistry of cleaved PARP and TUNEL in the castrated VCaP xenograft tumors.
(E) Percentage of body weight for VCaP-CRPC throughout the treatment period. Data are presented as mean ± SEM (n = 20 per condition).
(F) Representative tumor images from the vehicle, uprosertib, YJ1206, and combinatorial regimen in castrated VCaP CDX models (scale=200 μm). The inset scale=20 μm.
(G) H&E staining of normal organs from the vehicle, uprosertib, YJ1206, and combinatorial regimen in castrated VCaP CDX (scale=200 μm). The inset scale=20 μm.
(H) Tumor volume of 22Rv1 model with YJ1206 (p.o., 100 mg/kg, 3x/week) alone or in combination with uprosertib (p.o., 15 mg/kg, 5x/week) treatment. Data are mean ± SEM (n = 12 per condition; two-sided t-test).
(I) Tumor weights from castrated 22Rv1 xenograft study (two-sided t-test). Data are presented as mean ± SEM.
(J) Waterfall plot depicting the change in tumor volume after 24 days of treatment from 22Rv1 study.
(K) Percent body weight for 22Rv1 CRPC model throughout the treatment period. Data are presented as mean ± SEM.
(L-M) Representative H&E staining for 22Rv1 CRPC xenograft or PC310 PDX tumors at the end point (scale=200 μm). The inset scale=20 μm.
(N) Percent body weight for PC310 PDX throughout the treatment period. Data are presented as mean ± SEM (n = 6 per condition).

Table S1. Detailed oligonucleotides information. Related to STAR Methods and Key resources table.

Oligonucleotides	SOURCE	IDENTIFIER
*FANCI*_Fwd: CACCACACTTACAGCCCTTG	Quereda, V. et al[16]	N/A
*FANCI*_Rev: ATTCCTCCGGAGCTCTGAC	Quereda, V. et al[16]	N/A
*NRAS*_Fwd: GCGAAGGCTTCCTCTGTGTA	Quereda, V. et al[16]	N/A
*NRAS*_Rev: CTTGTTTCCCACTAGCACCA	Quereda, V. et al[16]	N/A
*RAD51*_Fwd: GCTGATGAGTTTGGTGTAGCAG	Quereda, V. et al[16]	N/A
*RAD51*_Rev: GGAAGACAGGGAGAGTCGTAGA	Quereda, V. et al[16]	N/A
*EZH2*_Fwd: GACCTCTGTCTTACTTGTGGAGC	This paper	N/A
*EZH2*_Rev: CGTCAGATGGTGCCAGCAATAG	This paper	N/A
*ACTB*_Fwd: AGGATGCAGAAGGAGATCACTG	This paper	N/A
*ACTB*_Rev: AGTACTTGCGCTCAGGAGGAG	This paper	N/A
*ATM exon1*_Fwd: GACCGCGTGATACTGGATG	This paper	N/A
*ATM exon1*_Rev: TCAAACCCTGCGTGACTG	This paper	N/A
*ATM exon10*_Fwd: GGCATAAATATTCCAGCAGACC	This paper	N/A
*ATM exon10*_Rev: TTCCATAGTAGGGACAACAACA	This paper	N/A
*ATR exon1*_Fwd: CCTGGGTCCTGCATCCT	This paper	N/A
*ATR exon1*_Rev: CAGCCATAGCGCAGCAG	This paper	N/A
*ATR exon5*_Fwd: CTACCAAAGTCAGCAGCTTTATC	This paper	N/A
*ATR exon5*_Rev: CATGATGTAGGATCAGGGAATGT	This paper	N/A
*NRAS exon1*_Fwd: GCTGTTCATGGCGGTTC	This paper	N/A
*NRAS exon1*_Rev: CTCAAGCTCCACTGCCT	This paper	N/A
*NRAS exon5*_Fwd: GTTCTTCCACAGCACAAACAC	This paper	N/A
*NRAS exon5*_Rev: ATCACCAGCAGTTGCTACTTTA	This paper	N/A
*ERGIC3 exon1*_Fwd: CACGAGCCATCCAGAAGAAA	This paper	N/A
*ERGIC3 exon1*_Rev: AGGGAGAAAGAGGAGACAGAG	This paper	N/A
*ERGIC3 exon3*_Fwd: TGCTTCCTCTGTCTCCTCTT	This paper	N/A
*ERGIC3 exon3*_Rev: CTGACCGAGGAGGTGGA	This paper	N/A
siRNA targeting sequence CDK12: CUACAGAGCGACUUCCUUA	Horizon Discovery	J-004031-10-0050
siRNA targeting sequence CDK13: GCUGAUAGCUUACGAGGAA	Horizon Discovery	J-004688-06-0050

Method S1. Chemical structures and synthesis of CDK12/13 degraders and analogues. Related to Figure 1 and Figure 4.

General information: All commercially available reagents and solvents were used without further purification. All chemical reactions were monitored by thin-layer chromatography (TLC) plates with visualization under UV light (254 or 365 nm). ^{1}H NMR spectra were performed with Bruker AV-400/600 spectrometer, and ^{13}C NMR spectra were recorded on Bruker AV-600 spectrometer at 150 MHz; internal reference was either TMS or deuterated NMR solvent. Low-resolution mass spectra (MS) were recorded on an Agilent 1200 HPLC-MSD mass spectrometer. High resolution mass spectral analysis was recorded on an Applied Biosystems Q-STAR Elite ESI-LC-MS/MS mass spectrometer. Purity of all final compounds was confirmed to be >95% by HPLC analysis with the Agilent 1260 system. The analytical columns were YMC-Triart C18 reversed-phase column, 5 μm, 4.6 mm × 250 mm, and flow rate 1.0 mL/min.

Scheme S1. Synthetic route of YJ9068, YJ9069, YJ1090, YJ1094, YJ1114, YJ1130, YJ1096, YJ1105, YJ1205, YJ1206 and YJ1078[a]

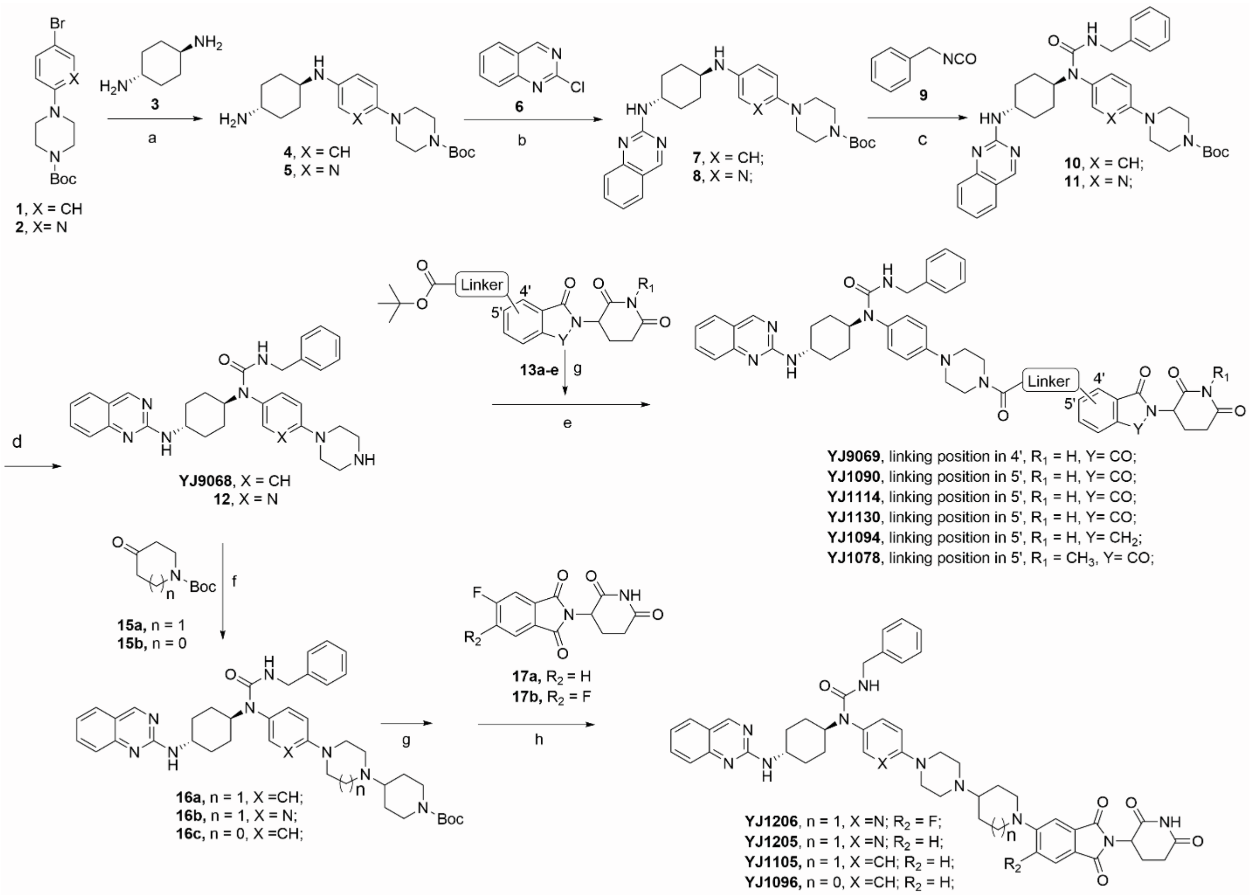

[a]Reagents and conditions: (a) *D*-proline, CuI, K_3PO_4, dry dimethyl sulfoxide (DMSO), 100 °C, 10 h, 44%; (b) Cs_2CO_3, *N,N*-dimethylformamide (DMF), 60 °C, 3 h, 90%; (c) *N,N*-diisopropylethylamine (DIPEA), DMF, 95 °C, 4 h, 62%; (d) TFA, DCM, 4 h, rt, 79%; (e) 2-(7-Azabenzotriazol-1-yl)-*N,',N',N'*-tetramethyluronium hexafluorophosphate (HATU), DIPEA, DMF, rt, 15 min, 75%; (f) DCM, Sodium triacetoxyborohydride, rt 52%; (g) TFA, DCM, 4 h, rt; (h) DMSO, DIPEA, 120 °C, 8 h, 63%.

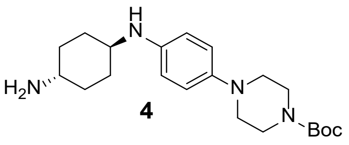

4

tert-butyl 4-(4-(((1r,4r)-4-aminocyclohexyl)amino)phenyl)piperazine-1-carboxylate (4)

Potassium phosphate (31.0 g, 146 mmol) was added to a solution of *trans*-cyclohexane-1,4-diamine (29.3 g, 256.4 mmol), *tert*-butyl 4-(4-bromophenyl)piperazine-1-carboxylate **3** (25.0 g, 73.26 mmol), CuI (1.4 g, 7.3 mmol), and *D*-Proline (843 mg, 7.3 mmol) in anhydrous DMSO (500 mL). The resulted suspension was then evacuated and backfilled with argon (3 cycles). The reaction mixture was then heated at 100 °C for 10 hours before being filtered through celite. The reaction solvent was evaporated under reduced pressure and purified by silica gel column chromatography to afford the title compound as a gray solid. (12.0 g, yield 44%): ^{1}H NMR (400 MHz, DMSO-d_6) δ 6.74 (d, J = 8.8 Hz, 2H), 6.49 (d, J = 8.9 Hz, 2H), 4.88 (d, J = 8.2 Hz, 1H), 3.42 (t, J = 5.1 Hz, 4H), 3.03 (s, 1H), 2.83 (t, J = 5.1 Hz, 4H), 2.76 (s, 1H), 1.95 (d, J = 12.8 Hz, 2H), 1.85 (d, J = 12.4 Hz, 2H), 1.41 (s, 9H), 1.26 (q, J = 10.9 Hz, 2H), 1.11 (q, J = 11.6 Hz, 2H). HRMS (ESI) for $C_{21}H_{34}N_4O_2$ $[M+H]^+$, calcd: 375.2755, found: 375.2739.

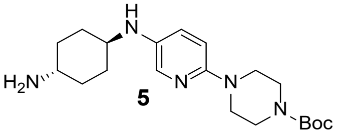

5

tert-butyl 4-(5-(((1r,4r)-4-aminocyclohexyl)amino)pyridin-2-yl)piperazine-1-carboxylate (5)

Compound **5** was synthesized by following a similar procedure as that of Compound **4**. ^{1}H NMR (400 MHz, DMSO-d_6) δ 7.60 (s, 1H), 6.94 (d, J = 8.9 Hz, 1H), 6.68 (d, J = 8.9 Hz, 1H), 4.83 (d, J = 8.5 Hz, 1H), 3.40 (t, J = 4.8 Hz, 4H), 3.19 (t, J = 4.8 Hz, 4H), 3.08 – 2.95 (m, 2H), 2.62 – 2.53 (m, 1H), 1.95 – 1.85 (m, 2H), 1.82 – 1.70 (m, 2H), 1.42 (s, 9H), 1.12 (q, J = 11.3 Hz, 4H).

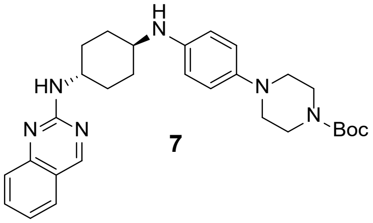

7

tert-butyl 4-(4-(((1r,4r)-4-(quinazolin-2-ylamino)cyclohexyl)amino)phenyl) piperazine-1-carboxylate (7)

To a solution of *tert*-butyl 4-(4-(((1r,4r)-4-aminocyclohexyl)amino)phenyl)piperazine-1-carboxylate **4** (9.0 g, 24 mmol) in DMF (50 mL) was added 2-chloroquinazoline (3.95 g, 24 mmol), Cs_2CO_3 (9.4 g, 28.9 mmol). The mixture was stirred at room temperature overnight. The reaction mixture was then filtered, and the solvent was removed under reduced pressure. The crude material was purified by column chromatography to afford **7** as white solid (10.8 g, yield 90%). ^{1}H NMR (400 MHz, DMSO-d_6) δ 9.09 (s, 1H), 7.77 (d, J = 8.0 Hz, 1H), 7.67 (t, J = 7.7 Hz, 1H), 7.45 (d, J = 8.5 Hz, 1H), 7.29 (d, J = 8.0 Hz, 1H), 7.20 (t, J = 7.4 Hz, 1H), 6.76 (d, J = 8.2 Hz, 2H), 6.52 (d, J = 8.3 Hz, 2H), 4.95 (s, 1H), 3.86 (d, J = 9.8 Hz, 1H), 3.43 (t, J = 5.0 Hz, 4H), 3.18 – 3.01 (m, 1H), 2.84 (s, 4H), 2.01 (d, J = 11.6 Hz, 4H), 1.50 – 1.32 (m, 11H), 1.30 – 1.15 (m, 2H).

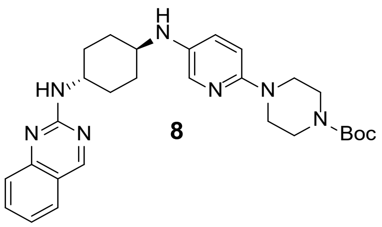

8

tert-butyl 4-(5-(((1r,4r)-4-(quinazolin-2-ylamino)cyclohexyl)amino)pyridin-2-yl)piperazine-1-carboxylate (8)

Compound **8** was synthesized by following a similar procedure as that of Compound **7**. ^{1}H NMR (400 MHz, DMSO-d_6) δ 9.09 (s, 1H), 7.77 (d, *J* = 8.0 Hz, 1H), 7.70 – 7.61 (m, 2H), 7.45 (d, *J* = 8.6 Hz, 1H), 7.29 (d, *J* = 7.9 Hz, 1H), 7.20 (t, *J* = 7.5 Hz, 1H), 6.98 (d, *J* = 8.6 Hz, 1H), 6.70 (d, *J* = 8.9 Hz, 1H), 4.92 (s, 1H), 3.93 – 3.80 (m, 1H), 3.40 (t, *J* = 5.4 Hz, 4H), 3.21 (t, *J* = 5.4 Hz, 4H), 3.17 – 3.06 (m, 1H), 2.02 (d, *J* = 12.1 Hz, 4H), 1.49 – 1.35 (m, 11H), 1.24 (q, *J* = 13.2, 12.4 Hz, 2H).

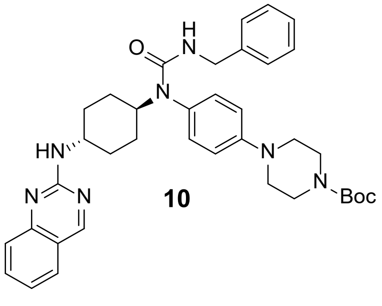

10

tert-butyl 4-(4-(3-benzyl-1-((1r,4r)-4-(quinazolin-2-ylamino)cyclohexyl)ureido) phenyl)piperazine-1-carboxylate (10)

To a solution of *tert*-butyl 4-(4-(((1r,4r)-4-(quinazolin-2-ylamino)cyclohexyl)amino)phenyl)piperazine-1-carboxylate **7** (10.0 g, 19.89 mmol) and DIPEA (7.7 g, 59.67 mmol) in DMF (20 mL) was added benzyl isocyanate (7.94 g, 59.67 mmol) at room temperature. The mixture was stirred at 95 °C for 4 hours. The solvent was removed under reduced pressure and purified by column chromatography to give a white solid (7.8 g, yield 62%). ^{1}H NMR (400 MHz, DMSO-d_6) δ 9.05 (s, 1H), 7.74 (d, *J* = 8.0 Hz, 1H), 7.63 (t, *J* = 7.8 Hz, 1H), 7.40 (d, *J* = 8.8 Hz, 1H), 7.31 – 7.22 (m, 3H), 7.21 – 7.13 (m, 4H), 7.08 – 6.98 (m, 4H), 5.58 (t, *J* = 6.1 Hz, 1H), 4.28 (t, *J* = 12.3 Hz, 1H), 4.16 (d, *J* = 5.8 Hz, 2H), 3.58 (s, 1H), 3.47 (t, *J* = 5.1 Hz, 4H), 3.18 (t, *J* = 5.2 Hz, 4H), 1.96 (d, *J* = 12.0 Hz, 2H), 1.79 (d, *J* = 12.3 Hz, 2H), 1.50 – 1.33 (m, 12H), 1.13 (q, *J* = 13.5, 12.5 Hz, 2H).

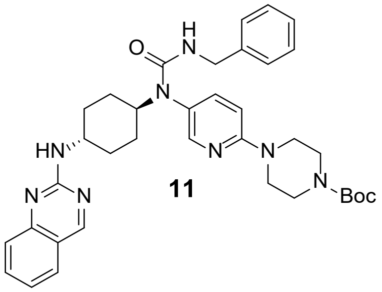

11

tert-butyl 4-(5-(3-benzyl-1-((1r,4r)-4-(quinazolin-2-ylamino)cyclohexyl)ureido) pyridin-2-yl)piperazine-1-carboxylate (11)

Compound **11** was synthesized by following a similar procedure as that of Compound **10**. ^{1}H NMR (400 MHz, DMSO-d_6) δ 9.05 (s, 1H), 7.93 (s, 1H), 7.74 (d, *J* = 8.0 Hz, 1H), 7.63 (t, *J* = 7.8 Hz, 1H), 7.39 (t, *J* = 9.1 Hz, 2H), 7.31 – 7.20 (m, 3H), 7.20 – 7.13 (m, 4H), 6.91 (d, *J* = 8.9 Hz, 1H), 6.05 (t, *J* = 6.1 Hz, 1H), 4.28 (t, *J* = 12.5 Hz, 1H), 4.15 (d, *J* = 6.0 Hz, 2H), 3.66 – 3.49 (m, 5H), 3.45 (t, *J* = 5.1 Hz, 4H), 2.07 – 1.89 (m, 2H), 1.79 (d, *J* = 12.3 Hz, 2H), 1.50 – 1.33 (m, 11H), 1.09 (q, *J* = 12.5 Hz, 2H).

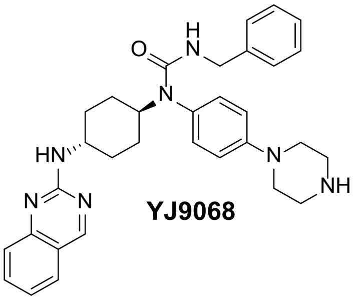

YJ9068

3-benzyl-1-(4-(piperazin-1-yl)phenyl)-1-((1r,4r)-4-(quinazolin-2-ylamino)cyclohexyl) urea (YJ9068)

TFA (7 mL) was added to a solution of *tert*-butyl 4-(4-(3-benzyl-1-((1r,4r)-4-(quinazolin-2-ylamino)cyclohexyl)ureido)phenyl)piperazine-1-carboxylate **10** (5 g, 7.86 mmol) in DCM (21 mL), and the mixture was stirred at 50 °C overnight. The reaction mixture was then concentrated to dryness under reduced pressure, and the resultant crude material was purified by column chromatography to afford the title compound as white solid (3.3 g, yield 79%).^{1}H NMR (400 MHz, DMSO-d_6) δ 9.05 (s, 1H), 7.74 (dd, J = 8.0, 1.5 Hz, 1H), 7.63 (t, J = 8.5 Hz, 1H), 7.41 (d, J = 8.5 Hz, 1H), 7.31 – 7.21 (m, 3H), 7.21 – 7.13 (m, 4H), 7.00 (q, J = 9.1 Hz, 4H), 5.56 (t, J = 6.1 Hz, 1H), 4.28 (tt, J = 12.1, 3.7 Hz, 1H), 4.16 (d, J = 6.0 Hz, 2H), 3.65 – 3.53 (m, 1H), 3.11 (dd, J = 6.3, 3.7 Hz, 4H), 2.84 (t, J = 5.0 Hz, 4H), 1.96 (d, J = 10.9 Hz, 2H), 1.79 (d, J = 10.8 Hz, 2H), 1.41 (q, J = 13.1 Hz, 2H), 1.13 (q, J = 13.0 Hz, 2H).

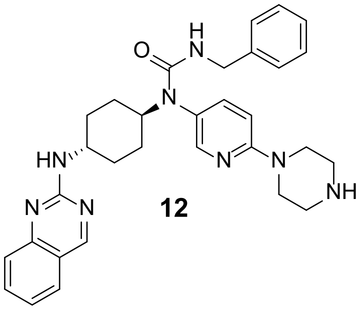

12

3-benzyl-1-(6-(piperazin-1-yl)pyridin-3-yl)-1-((1r,4r)-4-(quinazolin-2-ylamino) cyclohexyl)urea (12)

Compound **12** was synthesized by following a similar procedure as that of Compound **YJ9068**. ^{1}H NMR (400 MHz, DMSO-d_6) δ 9.05 (s, 1H), 7.94 (s, 1H), 7.75 (d, J = 8.0 Hz, 1H), 7.63 (t, J = 7.8 Hz, 1H), 7.39 (t, J = 8.5 Hz, 2H), 7.31 – 7.25 (m, 2H), 7.23 (d, J = 7.6 Hz, 1H), 7.21 – 7.13 (d, J = 7.4 Hz, 4H), 6.92 (d, J = 8.9 Hz, 1H), 6.04 (t, J = 6.2 Hz, 1H), 4.28 (t, J = 12.4 Hz, 1H), 4.16 (d, J = 5.9 Hz, 2H), 3.67 – 3.49 (m, 5H), 3.00 (t, J = 5.3 Hz, 4H), 1.98 (d, J = 12.3 Hz, 2H), 1.80 (d, J = 12.3 Hz, 2H), 1.42 (q, J = 12.4 Hz, 2H), 1.09 (q, J = 12.4 Hz, 2H).

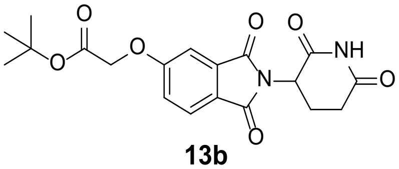

13b

tert-butyl 2-((2-(2,6-dioxopiperidin-3-yl)-1,3-dioxoisoindolin-5-yl)oxy)acetate (13b)

^{1}H NMR (400 MHz, DMSO-d_6) δ 11.11 (s, 1H), 7.81 (dd, J = 8.5, 7.3 Hz, 1H), 7.49 (d, J = 7.2 Hz, 1H), 7.38 (d, J = 8.6 Hz, 1H), 5.11 (dd, J = 12.9, 5.4 Hz, 1H), 4.97 (s, 2H), 2.96 – 2.83 (m, 1H), 2.65 – 2.53 (m, 2H), 2.10 – 1.99 (m, 1H), 1.43 (s, 9H).

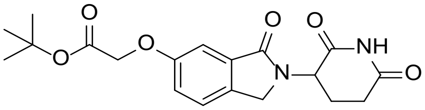

13c

tert-butyl 2-((2-(2,6-dioxopiperidin-3-yl)-3-oxoisoindolin-5-yl)oxy)acetate (13c)

^{1}H NMR (400 MHz, DMSO-d_6) δ 10.99 (s, 1H), 7.52 (d, J = 8.3 Hz, 1H), 7.21 (dd, J = 8.3, 2.5 Hz, 1H), 7.16 (d, J = 2.4 Hz, 1H), 5.10 (dd, J = 13.3, 5.1 Hz, 1H), 4.77 (s, 2H), 4.43 – 4.21 (m, 2H), 2.91 (ddd, J = 17.3, 13.6, 5.4 Hz, 1H), 2.66 – 2.56 (m, 1H), 2.39 (qd, J = 13.2, 4.4 Hz, 1H), 2.06 – 1.95 (m, 1H), 1.43 (s, 9H).

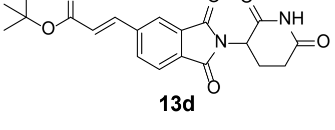

13d

tert-butyl (E)-3-(2-(2,6-dioxopiperidin-3-yl)-1,3-dioxoisoindolin-5-yl)acrylate (13d)

A mixture of 5-bromo-2-(2,6-dioxopiperidin-3-yl)isoindoline-1,3-dione (1 g, 2.95 mmol), *tert*-butyl acrylate (757.3 mg, 5.9 mmol), DIPEA (762.2 mg, 5.9 mmol), $Pd(AcO)_2$ (33.2 mg, 0.15 mmol), $P(ph)_3$ (77.5 mg, 0.3 mmol), and anhydrous DMF (40 mL) were added to a 100 mL round-bottom flask. The flask was evacuated and backfilled with argon (3 cycles). After stirring at 100 °C overnight, the mixture was then filtered and the solvent removed under vacuum. The crude material was purified by column chromatography to give **13d** as white solid (900 mg, yield 79%). ^{1}H NMR (400 MHz, DMSO-d_6) δ 11.14 (s, 1H), 8.30 (s, 1H), 8.19 (d, *J* = 7.8 Hz, 1H), 7.93 (d, *J* = 7.7 Hz, 1H), 7.74 (d, *J* = 16.1 Hz, 1H), 6.85 (d, *J* = 16.1 Hz, 1H), 5.17 (dd, *J* = 12.9, 5.4 Hz, 1H), 2.90 (ddd, *J* = 17.1, 13.8, 5.4 Hz, 1H), 2.65 – 2.52 (m, 2H), 2.08 (ddd, *J* = 12.6, 5.6, 3.3 Hz, 1H), 1.50 (s, 9H). MS (ESI), *m/z*: 382.8$[M-H]^-$.

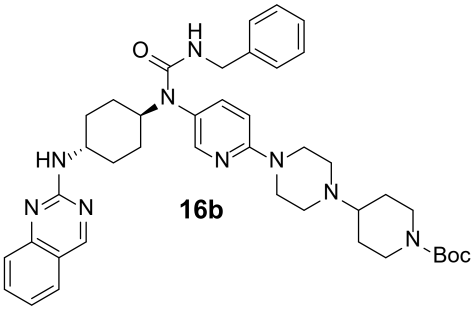

16b

tert-butyl 4-(4-(5-(3-benzyl-1-((1r,4r)-4-(quinazolin-2-ylamino)cyclohexyl) ureido)pyridin-2-yl)piperazin-1-yl)piperidine-1-carboxylate (16b)

To a solution of **13** (200 mg, 0.37 mmol) in DCM (20 mL) was added *tert*-butyl 4-oxopiperidine-1-carboxylate **15** (111 mg, 0.56 mmol). After stirring at room temperature for 30 minutes, then sodium triacetoxyborohydride (117.6 mg, 0.56 mmol) was added to the mixture. The reaction mixture was stirred for another 15 minutes then filtered, and the solvent was removed under reduced pressure. The crude material was purified by column chromatography to afford **16b** as white solid (138 mg, yield 52%).^{1}H NMR (400 MHz, DMSO-d_6) δ 9.05 (s, 1H), 7.91 (d, *J* = 2.6 Hz, 1H), 7.74 (d, *J* = 8.0 Hz, 1H), 7.63 (t, *J* = 7.9 Hz, 1H), 7.41 (d, *J* = 8.5 Hz, 1H), 7.37 – 7.31 (m, 1H), 7.26 (dt, *J* = 13.8, 7.8 Hz, 3H), 7.17 (d, *J* = 7.4 Hz, 4H), 6.88 (d, *J* = 9.0 Hz, 1H), 6.04 (t, *J* = 6.0 Hz, 1H), 4.29 (d, *J* = 12.0 Hz, 1H), 4.15 (d, *J* = 6.0 Hz, 2H), 3.60 (s, 2H), 3.52 (t, *J* = 5.2 Hz, 4H), 3.02 (d, *J* = 12.2 Hz, 2H), 2.58 (t, *J* = 5.1 Hz, 4H), 2.44 (dt, *J* = 14.3, 8.4 Hz, 3H), 2.32 (s, 1H), 1.97 (s, 2H), 1.84 – 1.69 (m, 4H), 1.51 – 1.33 (m, 11H), 1.29 – 1.21 (m, 2H),1.09 (q, *J* = 13.3 Hz, 2H).

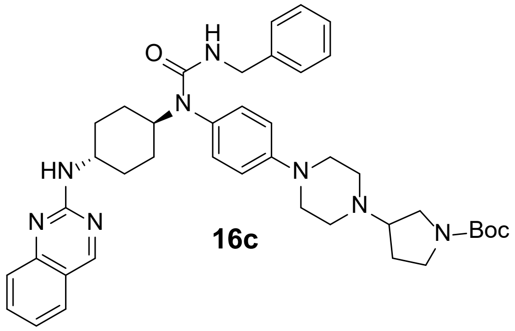

16c

tert-butyl 3-(4-(4-(3-benzyl-1-((1r,4r)-4-(quinazolin-2-ylamino)cyclohexyl)ureido)phenyl)piperazin-1-yl)pyrrolidine-1-carboxylate (16c)

Compound **16c** was synthesized by following a similar procedure as that of **16b**.^{1}H NMR (400 MHz, DMSO-d_6) δ 9.05 (s, 1H), 7.74 (d, *J* = 8.0 Hz, 1H), 7.63 (t, *J* = 7.7 Hz, 1H), 7.40 (d, *J* = 8.5 Hz, 1H), 7.31 – 7.25 (m, 2H), 7.22 (d, *J* = 7.8 Hz, 1H), 7.21 – 7.13 (m, 4H), 7.07 – 6.96 (m, 4H), 5.57 (t, *J* = 6.9 Hz, 1H), 4.28 (t, *J* = 11.4 Hz, 1H), 4.16 (d, *J* = 6.0 Hz, 2H), 3.65 – 3.45 (m, 3H), 3.41 (t, *J* = 9.7 Hz, 1H), 3.26 – 3.11 (m, 5H), 3.01 (q, *J* = 10.7 Hz, 1H), 2.88 – 2.73 (m, 1H), 2.65 – 2.56 (m, 2H), 2.12 – 1.89 (m, 4H), 1.74 – 1.62 (m, 1H), 1.72 (s, 1H), 1.50 – 1.32 (m, 11H), 1.13 (q, *J* = 13.3, 12.8 Hz, 2H).

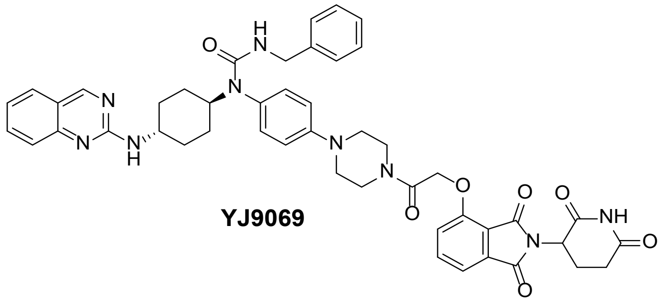

YJ9069

3-benzyl-1-(4-(4-(2-((2-(2,6-dioxopiperidin-3-yl)-1,3-dioxoisoindolin-4-yl)oxy) acetyl)piperazin-1-yl)phenyl)-1-((1r,4r)-4-(quinazolin-2-ylamino)cyclohexyl)urea (YJ9069)

To a solution of **YJ9068** (54 mg, 0.1 mmol), 2-((2-(2,6-dioxopiperidin-3-yl)-1,3-dioxoisoindolin-4-yl)oxy)acetic acid **13** (36.5 mg, 0.11), HATU (45.6 mg, 0.12 mmol), and DIPEA (25.8 mg, 0.2 mmol) in DMF (10 mL). The mixture was stirred at room temperature for 15 minutes and then evaporated under vacuum, purified by silica column chromatography to afford the compound **YJ9069** as a white solid (64 mg, yield 75%). ^{1}H NMR (400 MHz, DMSO-d_6) δ 11.12 (s, 1H), 9.05 (s, 1H), 7.79 (t, *J* = 7.9 Hz, 1H), 7.74 (d, *J* = 8.0 Hz, 1H), 7.63 (t, *J* = 7.8 Hz, 1H), 7.46 (d, *J* = 7.2 Hz, 1H), 7.39 (t, *J* = 8.1 Hz, 2H), 7.27 (q, *J* = 9.9, 8.7 Hz, 3H), 7.22 – 7.13 (m, 4H), 7.11 – 7.02 (m, 4H), 5.60 (t, *J* = 6.0 Hz, 1H), 5.26 (s, 2H), 5.12 (dd, *J* = 12.8, 5.4 Hz, 1H), 4.27 (d, *J* = 12.4 Hz, 1H), 4.17 (d, *J* = 6.0 Hz, 2H), 3.62 (s, 4H), 3.31 (s, 1H), 3.24 (s, 2H), 2.96 – 2.82 (m, 1H), 2.65 – 2.53 (m, 2H), 2.10 – 2.00 (m, 1H), 1.97 (d, *J* = 10.8 Hz, 2H), 1.80 (d, *J* = 11.8 Hz, 2H), 1.41 (q, *J* = 12.5 Hz, 2H), 1.13 (q, *J* = 12.3 Hz, 2H). ^{13}C NMR (151 MHz, DMSO-d_6) δ 173.31, 170.43, 167.29, 165.76, 165.61, 162.50, 157.33, 156.06, 150.34, 141.73, 137.09, 134.48, 133.55, 132.13 (2C), 129.17, 128.52 (3C), 128.32, 127.17 (4C), 126.75, 122.21, 120.67, 119.95, 116.60, 116.40 (2C), 116.00, 66.57, 53.54, 49.23, 49.13, 48.29, 48.16, 44.33, 43.94, 41.69, 40.043, 31.75, 31.40, 30.87 (2C), 22.46. HRMS (ESI) for $C_{47}H_{47}N_9O_7$[M+H]$^+$, calcd: 850.36712, found: 850.3646. HPLC analysis: MeOH-H_2O (80:20), 12.08 min, 97.4% purity.

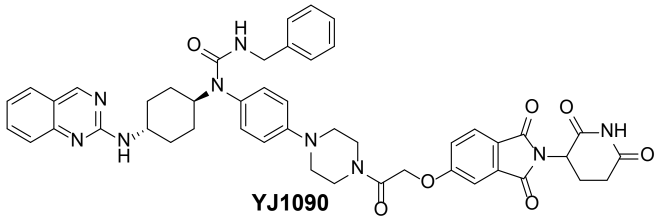

YJ1090

3-benzyl-1-(4-(4-(2-((2-(2,6-dioxopiperidin-3-yl)-1,3-dioxoisoindolin-5-yl)oxy)acetyl)piperazin-1-yl)phenyl)-1-((1r,4r)-4-(quinazolin-2-ylamino)cyclohexyl)urea (YJ1090)

Compound **YJ1090** was synthesized by following a similar procedure as that of **YJ9069**. ^{1}H NMR (400 MHz, DMSO-d_6) δ 11.11 (s, 1H), 9.05 (s, 1H), 7.85 (d, *J* = 8.3 Hz, 1H), 7.74 (d, *J* = 8.3 Hz, 1H), 7.63 (t, *J* = 7.9 Hz, 1H), 7.48 (d, *J* = 2.3 Hz, 1H), 7.43 – 7.35 (m, 2H), 7.32 – 7.24 (m, 3H), 7.23 (d, *J* = 8.2 Hz, 1H), 7.21 – 7.13 (m, 4H), 7.11 – 7.00 (m, 4H), 5.60 (t, *J* = 5.8 Hz, 1H), 5.21 (s, 2H), 5.12 (dd, *J* = 12.9, 5.4 Hz, 1H), 4.27 (t, *J* = 12.3 Hz, 1H), 4.17 (d, *J* = 6.0 Hz, 2H), 3.70 – 3.52 (m, 5H), 3.31 (s, 2H), 3.24 (s, 2H), 2.95 – 2.83 (m, 1H), 2.65 – 2.54 (m, 2H), 2.10 – 2.01 (m, 1H), 1.97 (d, *J* = 11.4 Hz, 2H), 1.80 (d, *J* = 11.9 Hz, 2H), 1.41 (q, *J* = 12.4 Hz, 2H), 1.14 (q, *J* = 12.0 Hz, 2H). ^{13}C NMR (151 MHz, DMSO-d_6) δ 173.30, 170.42, 167.42, 167.27, 165.69, 164.15, 162.50, 157.32, 150.32, 141.74, 134.47, 134.17, 132.13 (2C), 129.19, 128.52 (3C), 128.32, 127.17 (4C), 126.74, 125.65, 123.70, 122.21, 121.69, 119.95, 116.39 (2C), 109.58, 66.60, 53.54, 49.45, 49.13, 48.34, 48.16, 44.28, 43.94, 41.64, 40.44, 31.75, 31.41, 30.87 (2C), 22.52. HRMS (ESI) for $C_{47}H_{47}N_9O_7$[M+H]$^+$, calcd: 850.36712, found: 850.3633. HPLC analysis: MeOH-H_2O (75:25), 6.96 min, 95.0% purity.

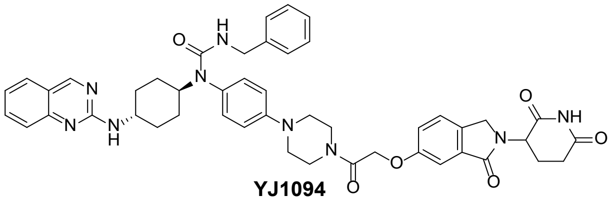

YJ1094

3-benzyl-1-(4-(4-(2-((2-(2,6-dioxopiperidin-3-yl)-3-oxoisoindolin-5-yl)oxy)acetyl)piperazin-1-yl)phenyl)-1-((1r,4r)-4-(quinazolin-2-ylamino)cyclohexyl)urea (YJ1094)

Compound **YJ1094** was synthesized by following a similar procedure as that of **YJ9069**.^{1}H NMR (400 MHz, DMSO-d_6) δ 10.98 (s, 1H), 9.05 (s, 1H), 7.74 (d, *J* = 7.9 Hz, 1H), 7.63 (t, *J* = 7.7 Hz, 1H), 7.51 (d, *J* = 8.2 Hz, 1H), 7.40 (d, *J* = 8.5 Hz, 1H), 7.31 – 7.21 (m, 5H), 7.21 – 7.13 (m, 4H), 7.08 – 7.00 (m, 4H), 5.60 (t, *J* = 5.9 Hz, 1H), 5.11 (dd, *J* = 13.2, 5.1 Hz, 1H), 5.03 (s, 2H), 4.43 – 4.21 (m, 3H), 4.16 (d, *J* = 6.0 Hz, 2H), 3.71 – 3.53 (m, 5H), 3.30 (s, 2H), 3.23 (s, 2H), 2.96 – 2.84 (m, 1H), 2.68 – 2.53 (m, 2H), 2.04 – 1.90 (s, 3H), 1.80 (d, *J* = 12.1 Hz, 2H), 1.41 (q, *J* = 12.3 Hz, 2H), 1.20 – 1.06 (m, 2H). ^{13}C NMR (151 MHz, DMSO-d_6) δ 173.41, 171.47, 168.57, 166.31, 162.50, 158.74, 157.33, 150.34, 141.75, 134.97, 134.48, 133.27, 132.11 (2C), 129.16, 128.52 (3C), 128.32, 127.16 (4C), 126.74, 124.92, 122.21, 120.65, 119.95, 116.38 (2C), 107.67, 66.52, 53.54, 52.21 (2C), 48.45, 48.18, 47.28, 44.44, 43.93, 41.63, 40.43, 31.74, 31.66, 30.86 (2C), 22.92. HRMS (ESI) for $C_{47}H_{49}N_9O_6$[M+H]$^+$, calcd: 836.38786, found: 836.3843. HPLC analysis: MeOH-H_2O (75:25), 6.33 min, 95.6% purity.

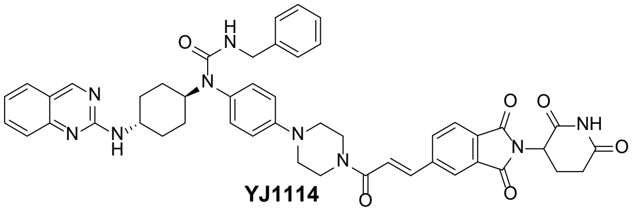

YJ1114

3-benzyl-1-(4-(4-((E)-3-(2-(2,6-dioxopiperidin-3-yl)-1,3-dioxoisoindolin-5-yl)acryloyl)piperazin-1-yl)phenyl)-1-((1r,4r)-4-(quinazolin-2-ylamino)cyclohexyl)urea (YJ1114)

Compound **YJ1114** was synthesized by following a similar procedure as that of **YJ9069**.^{1}H NMR (400 MHz, DMSO-d_6) δ 11.15 (s, 1H), 9.05 (s, 1H), 8.48 (s, 1H), 8.19 (d, *J* = 7.8 Hz, 1H), 7.96 (d, *J* = 7.7 Hz, 1H), 7.74 (d, *J* = 7.9 Hz, 1H), 7.71 (s, 2H), 7.63 (t, *J* = 7.8 Hz, 1H), 7.39 (d, *J* = 8.6 Hz, 1H), 7.31 – 7.21 (m, 3H), 7.21 – 7.13 (m, 4H), 7.07 (s, 4H), 5.59 (t, *J* = 6.4 Hz, 1H), 5.19 (dd, *J* = 13.0, 5.4 Hz, 1H), 4.27 (t, *J* = 11.9 Hz, 1H), 4.17 (d, *J* = 5.8 Hz, 2H), 3.95 (s, 2H), 3.76 (s, 2H), 3.58 (s, 1H), 2.97 – 2.83 (m, 1H), 2.69 – 2.56 (m, 2H), 2.13 – 2.04 (m, 2H), 1.96 (d, *J* = 11.9 Hz, 2H), 1.80 (d, *J* = 11.9 Hz, 2H), 1.41 (q, *J* = 12.1 Hz, 2H), 1.16 (q, *J* = 12.1 Hz, 2H). ^{13}C NMR (151 MHz, DMSO-d_6) δ 173.26, 170.31, 167.43, 167.23, 164.45, 162.49, 157.29, 150.37, 142.28, 141.79, 140.14, 135.37, 134.45, 132.59, 132.13 (2C), 131.66, 129.21, 128.51(3C), 128.31, 127.18(4C), 126.72, 124.38, 122.87, 122.63, 122.18, 119.95, 116.38 (2C), 53.54, 49.57 (2C), 48.90, 48.27, 45.38, 43.95, 42.19, 40.50, 31.76, 31.41, 30.88 (2C), 22.45. HRMS (ESI) for $C_{48}H_{47}N_9O_6$[M+H]$^+$, calcd: 846.37221, found: 846.3687. HPLC analysis: MeOH-H_2O (75:25), 14.00 min, 99.3% purity.

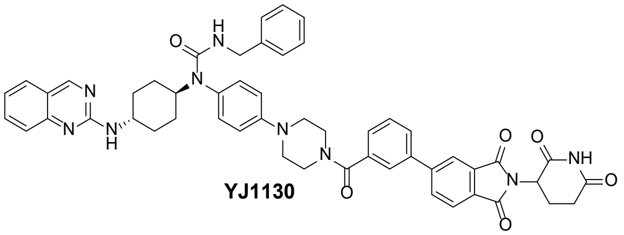

YJ1130

3-benzyl-1-(4-(4-(3-(2-(2,6-dioxopiperidin-3-yl)-1,3-dioxoisoindolin-5-yl)benzoyl)piperazin-1-yl)phenyl)-1-((1r,4r)-4-(quinazolin-2-ylamino)cyclohexyl)urea (YJ1130)

Compound **YJ1130** was synthesized by following a similar procedure as that of **YJ9069**.^{1}H NMR (400 MHz, DMSO-d_6) δ 11.15 (s, 1H), 9.05 (s, 1H), 8.30 – 8.21 (m, 2H), 8.03 (d, *J* = 7.7 Hz, 1H), 7.97 (d, *J* = 7.9 Hz, 1H), 7.92

(s, 1H), 7.75 (d, *J* = 8.1 Hz, 1H), 7.68 – 7.60 (m, 2H), 7.55 (d, *J* = 7.6 Hz, 1H), 7.40 (d, *J* = 8.5 Hz, 1H), 7.31 – 7.23 (m, 3H), 7.21 – 7.13 (m, 4H), 7.11 – 6.98 (m, 4H), 5.57 (t, *J* = 6.8 Hz, 1H), 5.19 (dd, *J* = 12.6, 5.3 Hz, 1H), 4.28 (t, *J* = 12.1 Hz, 1H), 4.16 (d, *J* = 5.7 Hz, 2H), 3.82 (s, 1H), 3.57 (s, 4H), 3.25 (s, 4H), 2.97 – 2.84 (m, 1H), 2.69 – 2.58 (m, 2H), 2.14 – 2.04 (m, 1H), 1.96 (d, *J* = 11.4 Hz, 2H), 1.80 (d, *J* = 12.0 Hz, 2H), 1.41 (q, *J* = 12.1 Hz, 2H), 1.14 (q, *J* = 12.1 Hz, 2H). ^{13}C NMR (151 MHz, DMSO-d_6) δ 173.26, 170.33, 169.02, 167.42, 167.39, 162.48, 157.27, 150.37, 146.31, 141.76, 138.78, 137.40, 134.44, 133.72, 132.81, 132.13 (2C), 130.67, 129.99, 129.25, 128.96, 128.51 (3C), 128.31, 127.93, 127.17 (4C), 126.72, 126.26, 124.58, 122.21, 122.18, 119.96, 116.46 (2C), 53.53, 49.57 (2C), 49.12, 48.28, 47.52, 43.95, 40.51, 31.75, 31.42 (2C), 30.88 (2C), 22.47. HRMS (ESI) for $C_{52}H_{49}N_9O_6[M+H]^+$, calcd: 896.38786, found: 896.3845. HPLC analysis: MeOH-H_2O (75:25), 19.82 min, 98.9% purity.

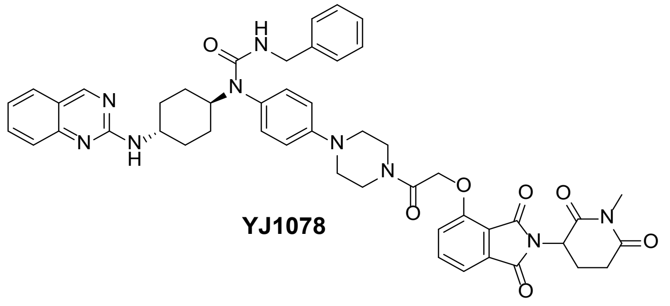

YJ1078

3-benzyl-1-(4-(4-(2-((2-(1-methyl-2,6-dioxopiperidin-3-yl)-1,3-dioxoisoindolin-4-yl)oxy)acetyl)piperazin-1-yl)phenyl)-1-((1r,4r)-4-(quinazolin-2-ylamino)cyclohexyl) urea (YJ1078)

Compound **YJ1078** was synthesized by following a similar procedure as that of **YJ9069**. ^{1}H NMR (400 MHz, DMSO-d_6) δ 9.05 (s, 1H), 7.79 (t, *J* = 7.8 Hz, 1H), 7.74 (d, *J* = 8.0 Hz, 1H), 7.63 (t, *J* = 7.8 Hz, 1H), 7.47 (d, *J* = 7.3 Hz, 1H), 7.39 (d, *J* = 8.4 Hz, 2H), 7.31 – 7.21 (m, 3H), 7.21 – 7.13 (m, 4H), 7.10 – 6.99 (s, 4H), 5.59 (t, *J* = 6.0 Hz, 1H), 5.25 (s, 2H), 5.18 (dd, *J* = 12.7, 5.0 Hz, 1H), 4.28 (t, *J* = 10.4 Hz, 1H), 4.17 (d, *J* = 6.0 Hz, 2H), 3.67 – 3.56 (m, 5H), 3.32 (s, 2H), 3.24 (s, 2H), 3.02 (s, 3H), 3.00 – 2.88 (m, 1H), 2.81 – 2.70 (m, 1H), 2.60 – 2.54 (m, 1H), 2.06 (d, *J* = 9.7 Hz, 1H), 1.97 (d, *J* = 11.6 Hz, 2H), 1.80 (d, *J* = 11.9 Hz, 2H), 1.42 (q, *J* = 12.8, 12.4 Hz, 2H), 1.14 (q, *J* = 12.3, 11.6 Hz, 2H). ^{13}C NMR (151 MHz, DMSO-d_6) δ 172.28, 170.18, 167.28, 165.76, 165.60, 162.50, 157.33, 156.07, 150.34, 141.72, 137.14, 134.48, 133.54, 132.12 (2C), 129.17, 128.52 (3C), 128.32, 127.17 (4C), 126.75, 122.21, 120.71, 119.95, 116.58, 116.39 (2C), 116.03, 66.61, 53.54, 49.81, 49.13, 48.31, 48.15, 44.34, 43.94, 41.70, 40.43, 31.75, 31.55, 30.87 (2C), 27.08, 21.67. HRMS (ESI) for $C_{48}H_{49}N_9O_7[M+H]^+$, calcd: 864.38277, found: 864.3804. HPLC analysis: MeOH-H_2O (75:25), 9.27 min, 97.5% purity.

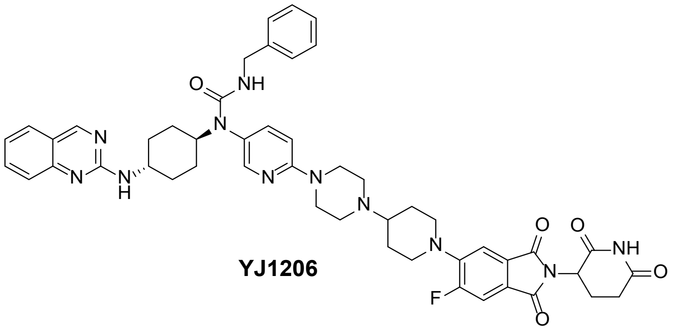

YJ1206

3-benzyl-1-(6-(4-(1-(2-(2,6-dioxopiperidin-3-yl)-6-fluoro-1,3-dioxoisoindolin-5-yl)piperidin-4-yl)piperazin-1-yl)pyridin-3-yl)-1-((1r,4r)-4-(quinazolin-2-ylamino) cyclohexyl)urea (YJ1206)

TFA (3 mL) was added to a suspension of **16b** (72 mg, 0.1 mmol) in DCM (6 mL). After stirring at room temperature for 3 hours, the reaction mixture was quenched with water and extracted with DCM three times. The combined organic phases were concentrated to dryness under reduced pressure. The resultant crude material was added to a suspension of compound **17b** (35 mg, 0.12 mmol) and DIPEA (39 mg, 0.3 mmol) in DMSO (10 mL). The resulting mixture was stirred at 120 °C for 8 h. The solvent was removed under vacuum to afford crude material which was purified by flash column chromatography to afford **YJ1206** as a yellow solid (56 mg, yield 63%). ^{1}H NMR (400 MHz, DMSO-d_6) δ 11.11 (s, 1H), 9.05 (s, 1H), 7.92 (s, 1H), 7.73 (t, *J* = 9.7 Hz, 2H), 7.63 (t, *J* = 8.0 Hz, 1H), 7.46 (d, *J* =

7.4 Hz, 1H), 7.41 (d, J = 8.5 Hz, 1H), 7.35 (d, J = 9.1 Hz, 1H), 7.31 – 7.25 (m, 2H), 7.21 (d, J = 7.5 Hz, 1H), 7.21 – 7.13 (m, 3H), 6.90 (d, J = 9.0 Hz, 1H), 6.05 (t, J = 5.4 Hz, 1H), 5.11 (dd, J = 13.0, 5.3 Hz, 1H), 4.28 (t, J = 12.3 Hz, 1H), 4.15 (d, J = 6.0 Hz, 2H), 3.68 (d, J = 11.9 Hz, 2H), 3.64 – 3.58 (m, 1H), 3.55(t, J = 4.5 Hz, 4H), 2.99 – 2.82 (m, 3H), 2.64 (s, 4H), 2.61 – 2.56 (m, 1H), 2.54(s, 2H), 2.08 – 1.87 (m, 5H), 1.79 (d, J = 11.7 Hz, 2H), 1.62 (q, J = 12.1, 11.3 Hz, 2H), 1.42 (q, J = 13.2, 12.5 Hz, 2H), 1.10 (q, J = 12.3 Hz, 2H). ^{13}C NMR (151 MHz, DMSO-d_6) δ 173.25, 170.40, 167.17, 166.69, 162.47, 158.33, 157.45, 156.89 (d, J = 253.4 Hz, 1C), 149.86, 146.00 (d, J = 8.49 Hz, 1C), 141.83, 140.60, 134.42, 129.26 (d, J = 2.2 Hz, 1C), 128.48 (3C), 128.30, 127.19 (4C), 126.69, 125.28, 124.42, 123.52 (d, J = 9.9 Hz, 1C), 122.17, 119.97, 114.24 (d, J = 4.5 Hz, 1C), 112.48 (d, J = 25.5 Hz, 1C), 107.40, 60.96, 55.39, 53.50, 49.84 (2C), 49.52 (2C), 49.11 (2C), 45.30, 43.97, 40.51, 31.72, 31.42, 30.85 (2C), 28.32 (2C), 22.55. HRMS (ESI) for $C_{49}H_{52}N_{11}O_5F[M+H]^+$, calcd: 894.42097, found: 894.4176. HPLC analysis: MeOH-H_2O (80:20), 8.79 min, 98.9% purity.

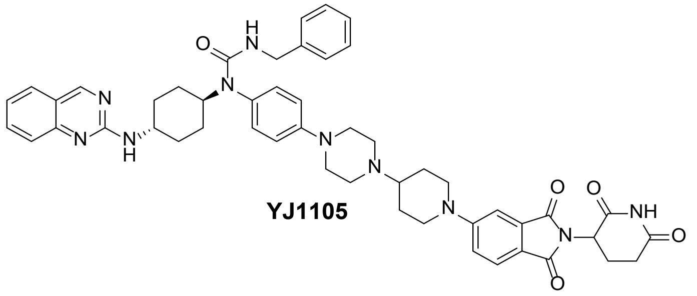

YJ1105

3-benzyl-1-(4-(4-(1-(2-(2,6-dioxopiperidin-3-yl)-1,3-dioxoisoindolin-5-yl)piperidin-4-yl)piperazin-1-yl)phenyl)-1-((1r,4r)-4-(quinazolin-2-ylamino)cyclohexyl)urea (YJ1105)

Compound **YJ1105** was synthesized by following a similar procedure as that of **YJ1206**.^{1}H NMR (400 MHz, DMSO-d_6) δ 11.08 (s, 1H), 9.05 (s, 1H), 7.74 (d, J = 8.0 Hz, 1H), 7.67 (d, J = 8.5 Hz, 1H), 7.63 (t, J = 7.5 Hz, 2H), 7.40 (d, J = 8.5 Hz, 1H), 7.34 (d, J = 2.2 Hz, 1H), 7.30 – 7.24 (m, 3H), 7.23 (d, J = 8.1 Hz, 1H), 7.20 – 7.12 (m, 4H), 7.01 (q, J = 8.8 Hz, 4H), 5.57 (t, J = 6.0 Hz, 1H), 5.07 (dd, J = 12.9, 5.4 Hz, 1H), 4.26 (t, J = 12.1 Hz, 1H), 4.16 (d, J = 6.0 Hz, 2H), 4.09 (d, J = 10.1 Hz, 2H), 3.65 – 3.53 (m, 1H), 3.26 – 3.12 (m, 4H), 3.00 (t, J = 12.3 Hz, 2H), 2.94 – 2.82 (m, 1H), 2.65 (t, J = 4.9 Hz, 4H), 2.62 – 2.59 (m, 1H), 2.59 – 2.53 (m, 2H), 2.07 – 1.86 (m, 5H), 1.78 (d, J = 11.6 Hz, 2H), 1.50 (q, J = 11.1 Hz, 2H), 1.41 (q, J = 12.5 Hz, 4H), 1.13 (q, J = 12.5, 11.9 Hz, 2H). ^{13}C NMR (151 MHz, DMSO-d_6) δ 173.37, 170.61, 168.11, 167.48, 162.50, 157.39, 155.22, 150.62, 141.70, 134.48, 131.99 (2C), 128.56, 128.52 (3C), 128.32, 127.14 (4C), 126.75, 125.53, 125.24, 122.23, 119.94, 118.17, 118.01, 115.79 (2C), 108.24, 60.99, 53.51, 49.20 (3C), 48.38 (2C), 47.06 (2C), 43.91, 40.73, 40.36, 31.75, 31.42, 30.85 (2C), 27.64 (2C), 22.65. HRMS (ESI) for $C_{50}H_{54}N_{10}O_5[M+H]^+$, calcd: 875.43514, found: 875.4322. HPLC analysis: MeOH-H_2O (80:20), 11.36 min, 99.2% purity.

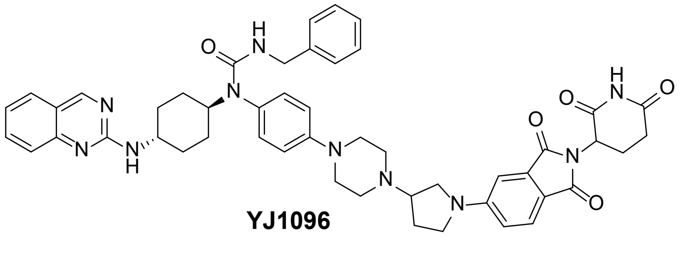

YJ1096

3-benzyl-1-(4-(4-(1-(2-(2,6-dioxopiperidin-3-yl)-1,3-dioxoisoindolin-5-yl)pyrrolidin-3-yl)piperazin-1-yl)phenyl)-1-((1r,4r)-4-(quinazolin-2-ylamino)cyclohexyl)urea (YJ1096)

Compound **YJ1096** was synthesized by following a similar procedure as that of **YJ1206**.^{1}H NMR (400 MHz, DMSO-d_6) δ 11.07 (s, 1H), 9.05 (s, 1H), 7.74 (d, J = 8.0 Hz, 1H), 7.68 – 7.59 (m, 2H), 7.40 (d, J = 8.4 Hz, 1H), 7.28 (t, J = 7.5 Hz, 2H), 7.23 (d, J = 8.1 Hz, 1H), 7.18 (t, J = 7.3 Hz, 4H), 7.04 (s, 4H), 6.99 (s, 1H), 6.87 (d, J = 9.0 Hz, 1H), 5.58 (d, J = 7.0 Hz, 1H), 5.06 (dd, J = 12.7, 5.4 Hz, 1H), 4.30 (t, J = 12.4 Hz, 1H), 4.16 (d, J = 5.9 Hz, 2H), 3.75 (t, J = 8.4 Hz, 1H), 3.66 – 3.53 (m, 2H), 3.42 (q, J = 8.8 Hz, 2H), 3.31 – 3.27 (m, 1H), 3.24 (t, J = 6.2 Hz, 4H), 3.02 (p, J = 7.6 Hz, 1H), 2.95 – 2.82 (m, 1H), 2.67 (s, 4H), 2.63 – 2.53 (m, 2H), 2.34 – 2.26 (m, 1H), 2.05 – 1.88 (m, 4H), 1.79 (d, J = 11.7 Hz, 2H), 1.41 (q, J = 12.4 Hz, 2H), 1.13 (q, J = 12.8 Hz, 2H). ^{13}C NMR (151 MHz, DMSO-d_6) δ

173.35, 170.65, 168.20, 167.74, 162.49, 157.36, 152.27, 150.57, 141.76, 134.46, 132.03 (2C), 128.74, 128.52 (4C), 128.31, 127.15 (4C), 126.74, 125.41, 122.21, 119.95, 116.22, 115.92 (2C), 115.71, 106.05, 63.97, 53.52, 52.42, 51.76 (2C), 49.15 (2C), 48.05 (2C), 47.49, 43.93, 40.43, 31.76, 31.45, 30.87 (2C), 29.28, 22.71. HRMS (ESI) for $C_{49}H_{52}N_{10}O_5[M+H]^+$, calcd: 861.41949, found: 861.4168. HPLC analysis: MeOH-H_2O (75:25), 15.48 min, 98.2% purity.

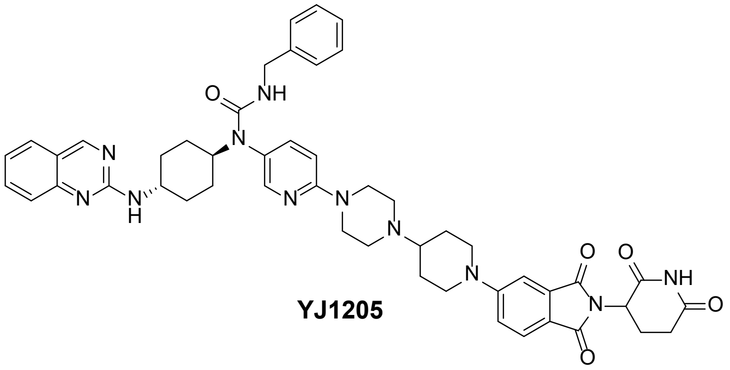

YJ1205

3-benzyl-1-(6-(4-(1-(2-(2,6-dioxopiperidin-3-yl)-1,3-dioxoisoindolin-5-yl)piperidin-4-yl)piperazin-1-yl)pyridin-3-yl)-1-((1r,4r)-4-(quinazolin-2-ylamino)cyclohexyl)urea (YJZ1205)

Compound **YJ1205** was synthesized by following a similar procedure as that of **YJ1206**. ^{1}H NMR (400 MHz, DMSO-d_6) δ 11.08 (s, 1H), 9.05 (s, 1H), 7.91 (s, 1H), 7.74 (d, J = 8.0 Hz, 1H), 7.70 – 7.58 (m, 2H), 7.40 (d, J = 8.4 Hz, 1H), 7.34 (s, 2H), 7.31 – 7.21 (m, 4H), 7.21 – 7.13 (m, 4H), 6.88 (d, J = 9.0 Hz, 1H), 6.05 (t, J = 6.6 Hz, 1H), 5.07 (dd, J = 12.8, 5.3 Hz, 1H), 4.26 (t, J = 12.8 Hz, 1H), 4.12 (dd, J = 18.7, 9.2 Hz, 4H), 3.60 (s, 1H), 3.52 (s, 4H), 3.00 (t, J = 12.4 Hz, 2H), 2.94 – 2.81 (m, 1H), 2.70 – 2.53 (m, 7H), 2.07 – 1.93 (m, 3H), 1.90 (d, J = 12.2 Hz, 2H), 1.78 (d, J = 11.7 Hz, 2H), 1.57 – 1.33 (m, 4H), 1.09 (q, J = 12.9 Hz, 2H). ^{13}C NMR (151 MHz, DMSO-d_6) δ 173.30, 170.59, 168.09, 167.44, 162.47, 158.32, 157.45, 155.22, 149.85, 141.82, 140.60, 134.51, 134.43, 128.48 (3C), 128.31, 127.18 (4C), 126.68, 125.49, 124.43, 122.17, 119.97, 118.17, 118.09, 108.28, 107.40, 61.12, 55.39, 53.49, 49.21(2C), 49.10 (2C), 47.10 (2C), 45.29, 43.96, 31.70, 31.45, 30.85 (2C), 27.66 (2C), 22.66. HRMS (ESI) for $C_{49}H_{53}N_{11}O_5[M+H]^+$, calcd: 876.43039, found: 876.4276. HPLC analysis: MeOH-H_2O (80:20), 6.94 min, 97.8% purity.

The ^{1}H NMR, ^{13}C NMR, HRMS, and HPLC traces of compounds YJ9069, YJ1078, and YJ1206.

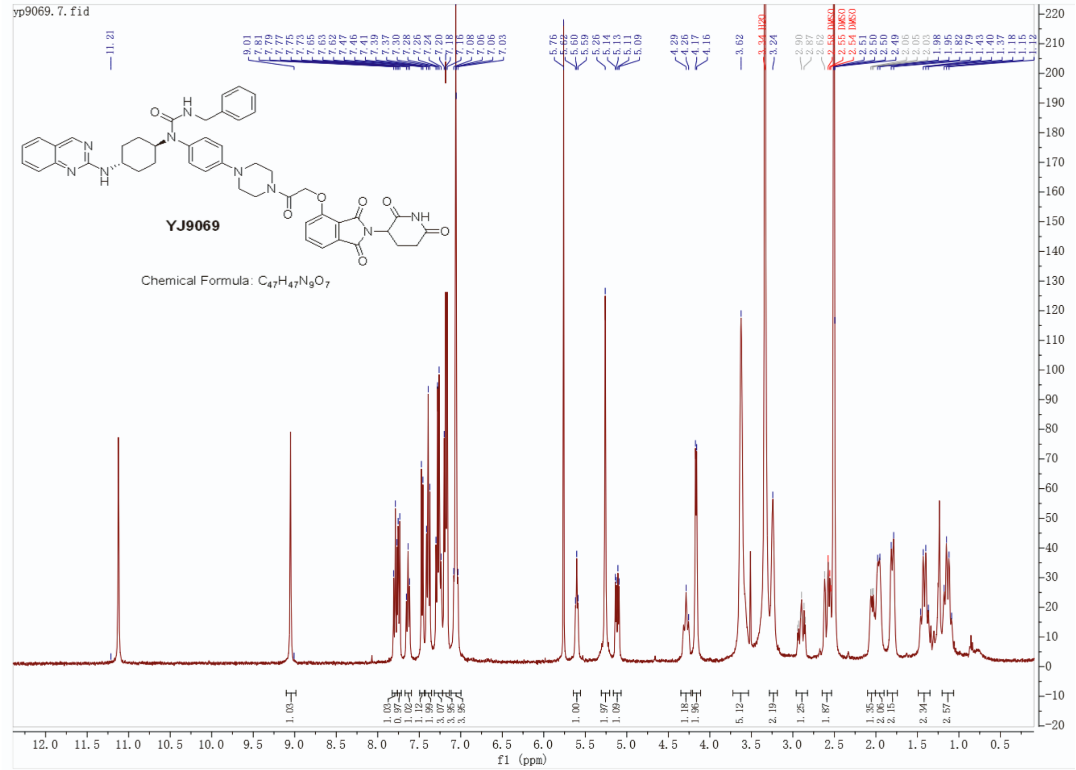

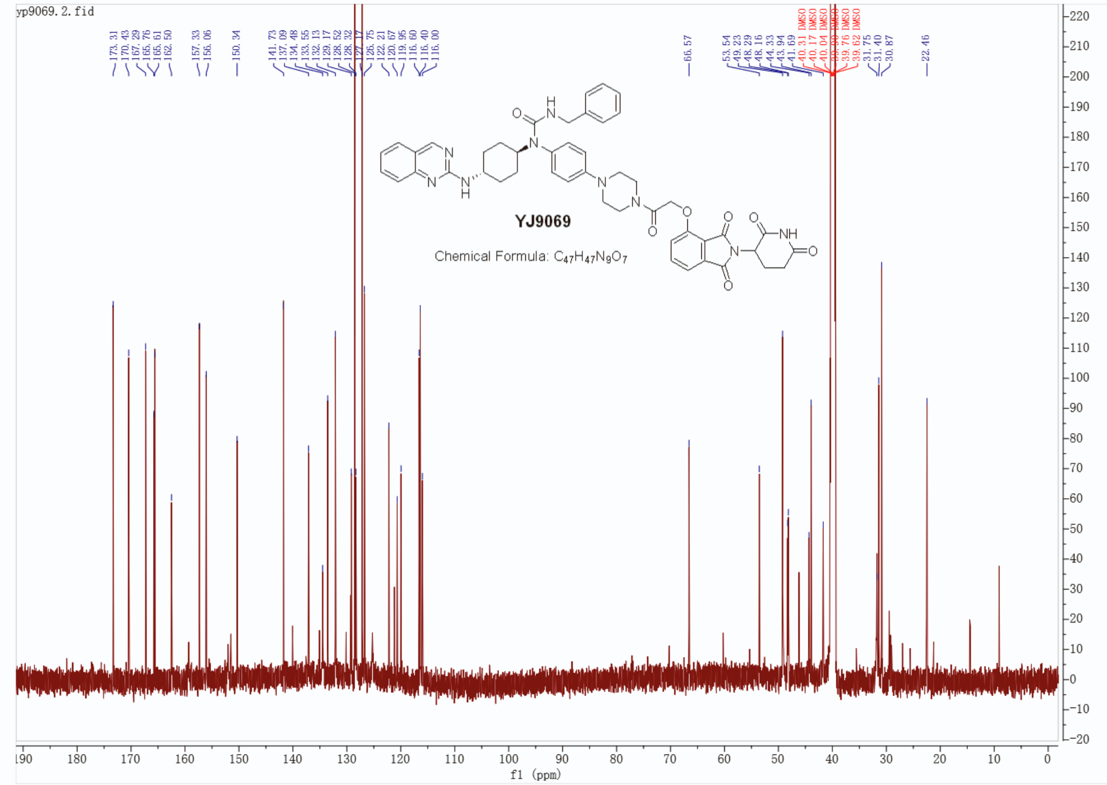

Data File E:\DK\YJZ\data\20210904\4996.D
Sample Name: 9069

===

Acq. Operator : 系统
Sample Operator : 系统
Acq. Instrument : 1260LC Location : 11
Injection Date : 04/09/2021 14:49:45
Inj Volume : 10.000 µl
Method : E:\DK\TL\方法\70C-30D-30min-1u.M
Last changed : 04/09/2021 14:22:01 by 系统
(modified after loading)

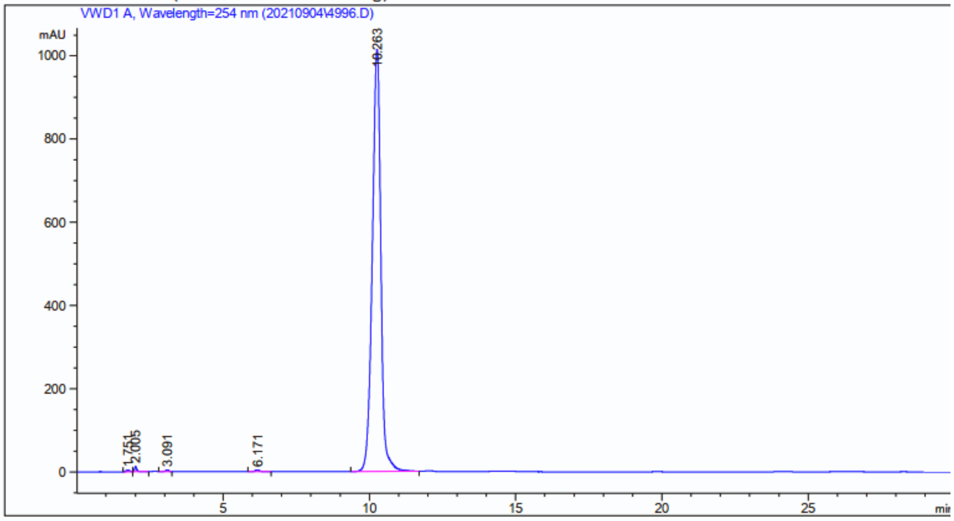

===

Area Percent Report

===

Sorted By : Signal
Multiplier : 1.0000
Dilution : 1.0000
Sample Amount: : 10.00000 [ng/ul] (not used in calc.)
Use Multiplier & Dilution Factor with ISTDs

Signal 1: VWD1 A, Wavelength=254 nm

Peak #	RetTime [min]	Type	Width [min]	Area [mAU*s]	Height [mAU]	Area %
1	1.751	BV	0.1065	36.82307	4.80255	0.1776
2	2.005	VB	0.0835	69.05422	12.48212	0.3330
3	3.091	VB	0.1160	29.43379	3.77336	0.1419
4	6.171	BB	0.1584	44.70434	4.22628	0.2156
5	10.263	BB	0.3102	2.05592e4	1015.45947	99.1320

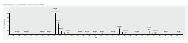

Elemental Composition Results - Spectrum 1

Peak Mass	Display Formula	S Fit	RDB	Delta [ppm]	Theo. mass	Rank	Combined Score	# Matched Iso.	# Missed Iso.	MS Cov. [%]	Pattern Cov. [%]
850.3646	$C_{46}H_{52}O_{11}N_5$	68.80	23.50	-1.36	850.36578	1	98.36	5	0	100.00	100.00
850.3646	$C_{45}H_{56}O_{15}N$	65.54	18.50	0.21	850.36445	2	98.19	5	0	100.00	100.00
850.3646	$C_{47}H_{48}O_7N_9$	59.19	28.50	-2.94	850.36712	3	97.85	5	0	100.00	100.00
850.3646	$C_{58}H_{48}O_4N_3$	28.76	36.50	0.81	850.36393	4	94.19	5	0	97.82	97.12
850.3646	$C_{41}H_{52}O_{13}N_7$	24.88	19.50	3.37	850.36176	5	85.54	5	0	88.91	89.93

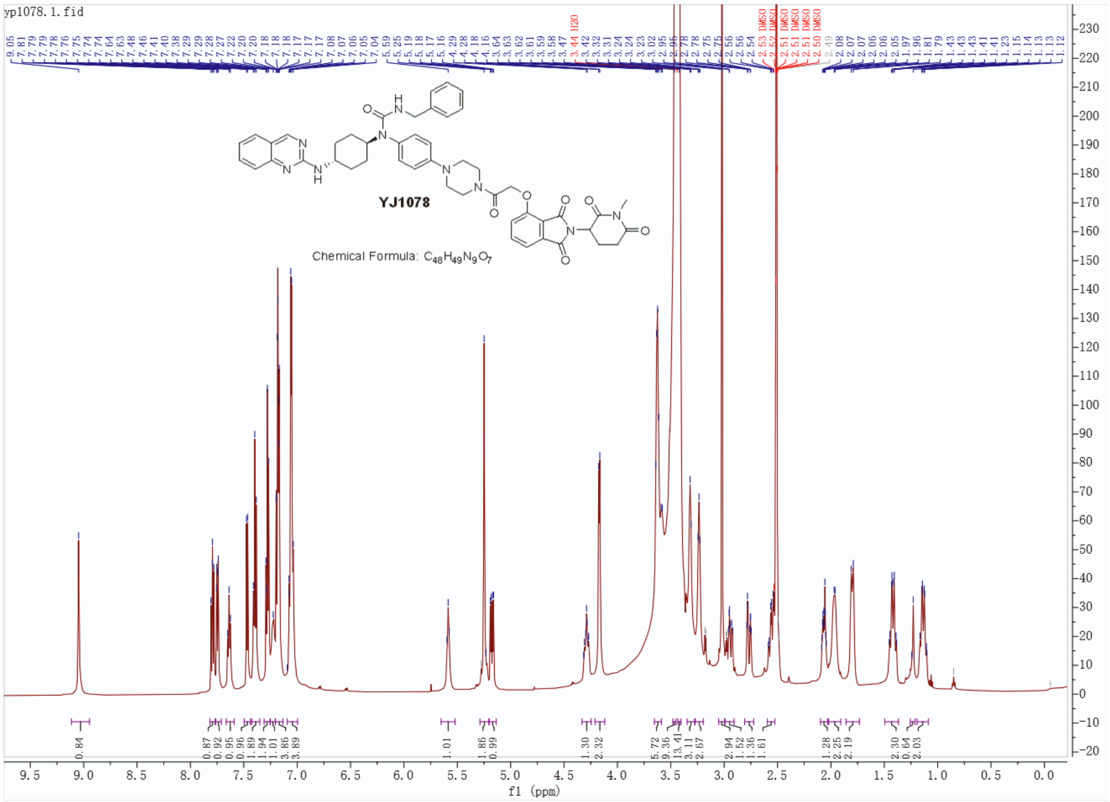
yp1078.1.fid
YJ1078
Chemical Formula: $C_{48}H_{49}N_9O_7$
f1 (ppm)

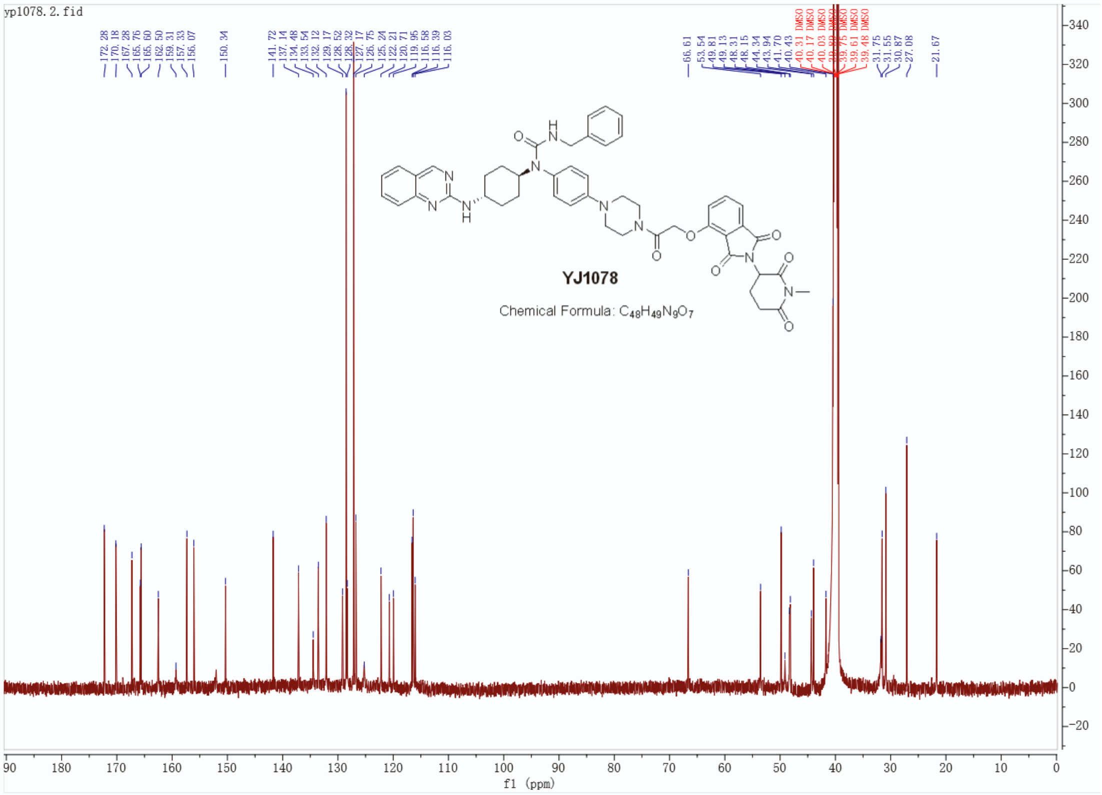
yp1078.2.fid
YJ1078
Chemical Formula: $C_{48}H_{49}N_9O_7$
f1 (ppm)

Data File E:\DK\YJZ\data\20221017\YJZ1901DEF_LC 2022-10-17 21-09-45\008-88-1078.D
Sample Name: 1078

===

Acq. Operator : 系统 Seq. Line : 8
Acq. Instrument : 1260LC Location : 88
Injection Date : 18/10/2022 00:53:15 Inj : 1
Inj Volume : 5.000 µl
Different Inj Volume from Sample Entry! Actual Inj Volume : 10.000 µl
Method : E:\DK\YJZ\data\20221017\YJZ1901DEF_LC 2022-10-17 21-09-45\75C-25D-30min-Y.M
(Sequence Method)
Last changed : 17/10/2022 21:09:45 by 系统

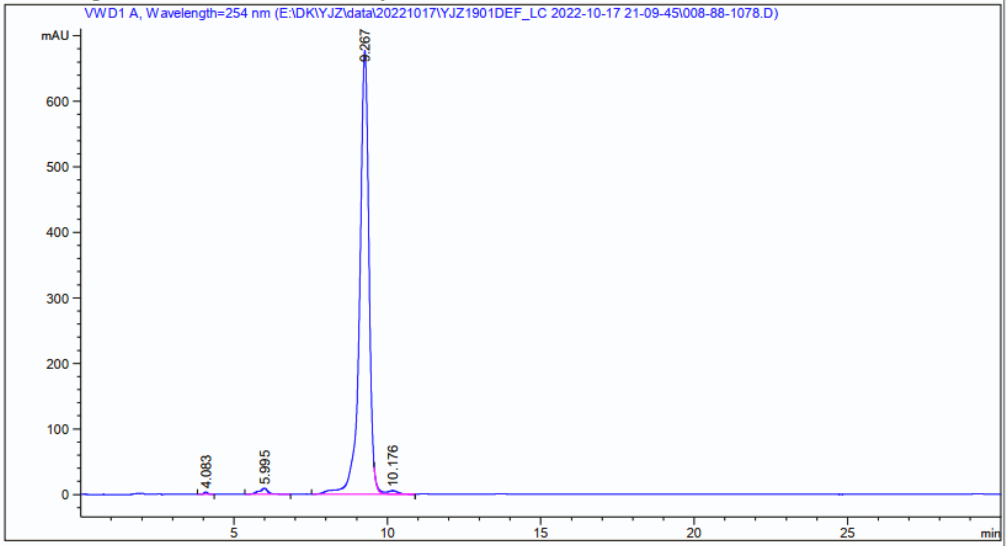

===
Area Percent Report
===

Sorted By : Signal
Multiplier : 1.0000
Dilution : 1.0000
Use Multiplier & Dilution Factor with ISTDs

Signal 1: VWD1 A, Wavelength=254 nm

Peak #	RetTime [min]	Type	Width [min]	Area [mAU*s]	Height [mAU]	Area %
1	4.083	BB	0.1157	27.69697	3.64108	0.1890
2	5.995	BB	0.2804	186.65962	9.25254	1.2735
3	9.267	BV R	0.3182	1.42854e4	676.94824	97.4599
4	10.176	VB E	0.4224	157.96930	5.28968	1.0777

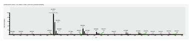

Elemental Composition Results - Spectrum 1

Peak Mass	Display Formula	S Fit	RDB	Delta [ppm]	Theo. mass	Rank	Combined Score	# Matched Iso.	# Missed Iso.	MS Cov. [%]	Pattern Cov. [%]
864.3804	$C_{47}H_{54}O_{11}N_5$	65.96	23.50	-1.16	864.38143	1	98.15	5	0	99.94	100.00
864.3804	$C_{48}H_{50}O_7N_9$	59.89	28.50	-2.71	864.38277	2	97.83	5	0	99.94	100.00
864.3804	$C_{46}H_{58}O_{15}N$	54.23	18.50	0.39	864.38010	3	97.34	6	0	99.74	99.61
864.3804	$C_{42}H_{54}O_{13}N_7$	32.12	19.50	3.50	864.37741	4	96.12	5	0	99.68	99.69
864.3804	$C_{59}H_{50}O_4N_3$	35.04	36.50	0.98	864.37958	5	94.71	6	0	98.03	97.00

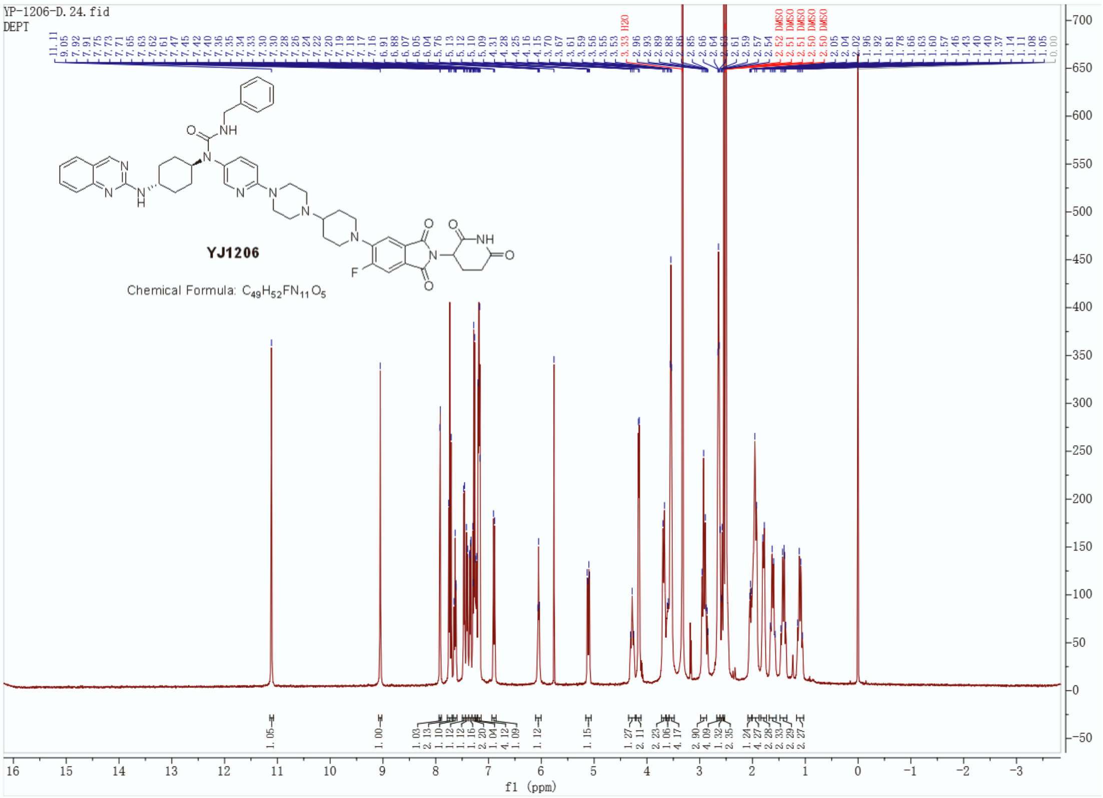

YP-1206-D.24.fid
DEPT
YJ1206
Chemical Formula: C49H52FN11O5
f1 (ppm)

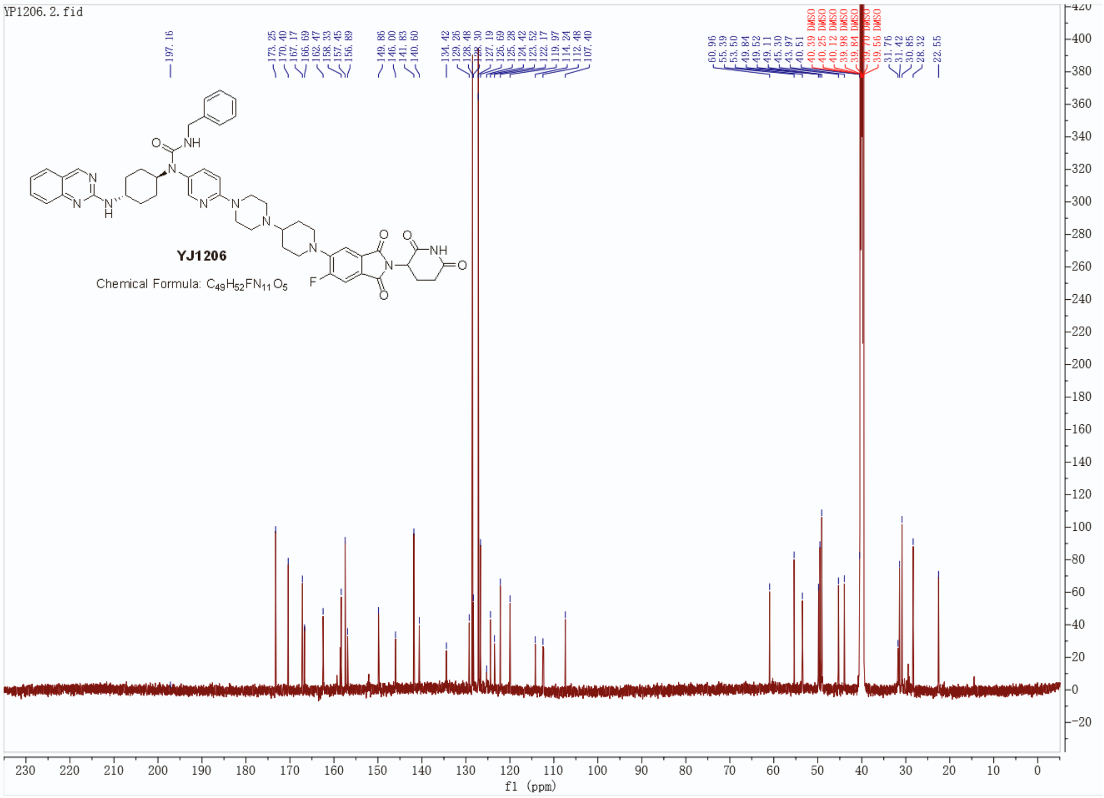

YP1206.2.fid
YJ1206
Chemical Formula: C49H52FN11O5
f1 (ppm)

Data File E:\DK\YJZ\data\20221019\YJZ1901DEF_LC 2022-10-19 17-10-43\004-77-1206.D
Sample Name: 1206

===

Acq. Operator : 系统 Seq. Line : 4
Acq. Instrument : 1260LC Location : 77
Injection Date : 19/10/2022 18:14:13 Inj : 1
Inj Volume : 5.000 µl
Different Inj Volume from Sample Entry! Actual Inj Volume : 10.000 µl
Method : E:\DK\YJZ\data\20221019\YJZ1901DEF_LC 2022-10-19 17-10-43\80C-20D-20MIN-20UL.M (Sequence Method)
Last changed : 19/10/2022 17:10:43 by 系统

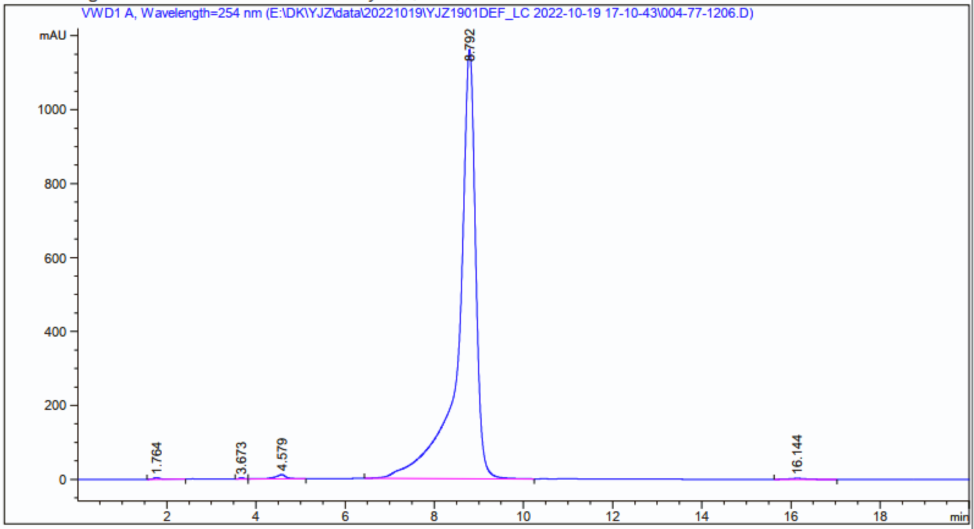

===

Area Percent Report

===

Sorted By : Signal
Multiplier : 1.0000
Dilution : 1.0000
Use Multiplier & Dilution Factor with ISTDs

Signal 1: VWD1 A, Wavelength=254 nm

Peak #	RetTime [min]	Type	Width [min]	Area [mAU*s]	Height [mAU]	Area %
1	1.764	BV R	0.1694	51.99037	4.20069	0.1647
2	3.673	BV	0.1049	27.78824	3.95838	0.0880
3	4.579	VB R	0.2552	219.13762	11.82306	0.6941
4	8.792	BB	0.3837	3.12137e4	1160.87976	98.8703
5	16.144	BB	0.3604	57.72878	2.45443	0.1829

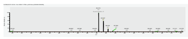

Elemental Composition Results - Spectrum 1

Peak Mass	Display Formula	S Fit	RDB	Delta [ppm]	Theo. mass	Rank	Combined Score	# Matched Iso.	# Missed Iso.	MS Cov. [%]	Pattern Cov. [%]
894.4176	$C_{52}H_{52}O_4N_{11}$	66.86	32.50	-2.49	894.41983	5	98.26	5	0	100.00	100.00
894.4176	$C_{44}H_{62}O_{14}N_3F_2$	62.02	14.50	-2.05	894.41944	6	98.00	5	0	100.00	100.00
894.4176	$C_{48}H_{48}O_2N_{17}$	60.97	33.50	0.52	894.41714	7	97.95	5	0	100.00	100.00
894.4176	$C_{57}H_{54}O_3N_5F_2$	59.22	32.50	-1.48	894.41892	8	97.85	5	0	100.00	100.00
894.4176	$C_{47}H_{52}O_6N_{13}$	53.94	28.50	2.01	894.41580	9	97.58	5	0	100.00	100.00
894.4176	$C_{49}H_{53}O_5N_{11}F$	52.01	28.50	-3.76	894.42097	10	97.47	5	0	100.00	100.00
