# Supplementary material for: Development of an orally bioavailable CDK12/13 degrader and induction of synthetic lethality with AKT pathway inhibition
Source: Cell Rep Med. 2024 Sep 30;5(10):101752. doi: 10.1016/j.xcrm.2024.101752 (PMC11513842; doi:10.1016/j.xcrm.2024.101752)
Supplement: Document S2. Article plus supplemental information [file mmc2.pdf]

Cell Reports Medicine

Article

Development of an orally bioavailable CDK12/13 degrader and induction of synthetic lethality with AKT pathway inhibition

Graphical abstract

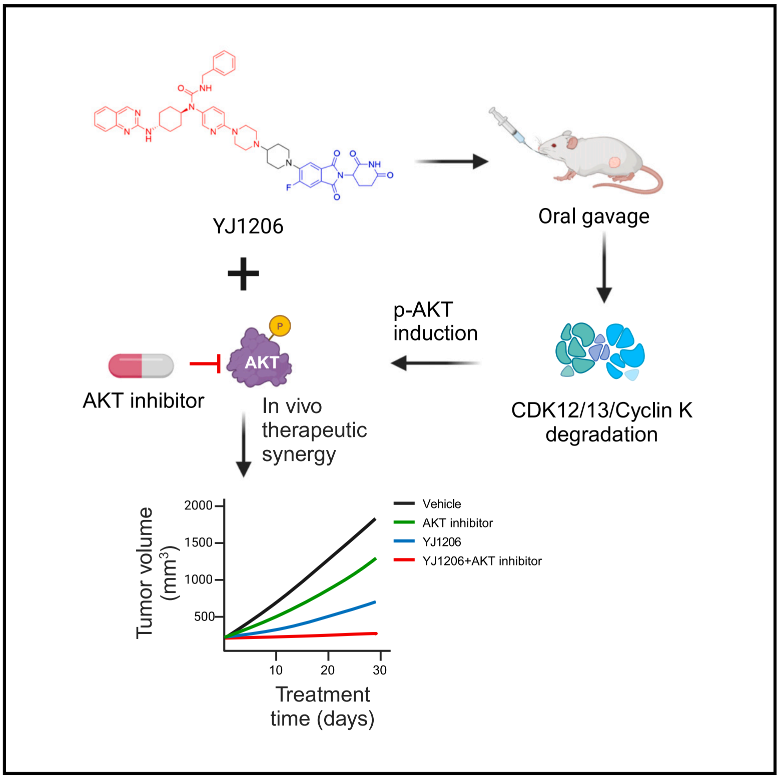

Highlights

- Development of an orally bioavailable CDK12/13 degrader (YJ1206)
- YJ1206 exhibits minimal adverse effects in immune-competent mice
- YJ1206 is efficacious in preclinical models of advanced prostate cancer
- YJ1206 induces synthetic lethality in conjunction with AKT pathway inhibition

Authors

Yu Chang, Xiaoju Wang, Jianzhang Yang, ..., Rohit Mehra, Ke Ding, Arul M. Chinnaiyan

Correspondence

dingk@sioc.ac.cn (K.D.), arul@umich.edu (A.M.C.)

In brief

Chang et al. develop YJ1206, an orally bioavailable degrader that exhibits high specificity in degrading CDK12/13 and demonstrates robust anti-tumor activity *in vivo*. The degradation of CDK12/13 leads to activation of the AKT pathway. YJ1206 in combination with AKT inhibitors achieves a striking synthetic lethal effect in prostate cancer models.

Chang et al., 2024, Cell Reports Medicine *5*, 101752
October 15, 2024 © 2024 The Author(s). Published by Elsevier Inc.
https://doi.org/10.1016/j.xcrm.2024.101752

CellPress

Cell Reports Medicine

CellPress
OPEN ACCESS

Article

Development of an orally bioavailable CDK12/13 degrader and induction of synthetic lethality with AKT pathway inhibition

Yu Chang,[1,2,8] Xiaoju Wang,[1,2,3,8] Jianzhang Yang,[4,5,8] Jean Ching-Yi Tien,[1,2] Rahul Mannan,[1,2] Gabriel Cruz,[1,2] Yuping Zhang,[1,2] Josh N. Vo,[1,2] Brian Magnuson,[1,2] Somnath Mahapatra,[1,2] Hanbyul Cho,[1,2] Saravana Mohan Dhanasekaran,[1,2,3] Cynthia Wang,[1,2] Zhen Wang,[4] Licheng Zhou,[4,5] Kaijie Zhou,[4] Yang Zhou,[5] Pujuan Zhang,[4] Weixue Huang,[4] Lanbo Xiao,[1,2] Weihuang Raymond Liu,[1] Rudana Hamadeh,[1] Fengyun Su,[1,2] Rui Wang,[1,2] Stephanie J. Miner,[1,2] Xuhong Cao,[1,2,3,6] Yunhui Cheng,[1,2] Rohit Mehra,[1,2,3,7] Ke Ding,[4,*] and Arul M. Chinnaiyan[1,2,3,6,7,9,*]

[1]Michigan Center for Translational Pathology, University of Michigan, Ann Arbor, MI 48109, USA
[2]Department of Pathology, University of Michigan, Ann Arbor, MI 48109, USA
[3]Rogel Cancer Center, University of Michigan, Ann Arbor, MI 48109, USA
[4]State Key Laboratory of Chemical Biology, Shanghai Institute of Organic Chemistry, Chinese Academy of Sciences, Shanghai 200032, People's Republic of China
[5]School of Pharmaceutical Sciences, Jinan University, Guangzhou 511436, People's Republic of China
[6]Howard Hughes Medical Institute, University of Michigan, Ann Arbor, MI 48109, USA
[7]Department of Urology, University of Michigan, Ann Arbor, MI 48109, USA
[8]These authors contributed equally
[9]Lead contact
*Correspondence: dingk@sioc.ac.cn (K.D.), arul@umich.edu (A.M.C.)
https://doi.org/10.1016/j.xcrm.2024.101752

SUMMARY

Cyclin-dependent kinases 12/13 play pivotal roles in orchestrating transcription elongation, DNA damage response, and maintenance of genomic stability. Biallelic *CDK12* loss has been documented in various malignancies. Here, we develop a selective CDK12/13 PROTAC degrader, YJ9069, which effectively inhibits proliferation in subsets of prostate cancer cells preferentially over benign immortalized cells. CDK12/13 degradation rapidly triggers gene-length-dependent transcriptional elongation defects, leading to DNA damage and cell-cycle arrest. *In vivo*, YJ9069 significantly suppresses prostate tumor growth. Modifications of YJ9069 yielded an orally bioavailable CDK12/13 degrader, YJ1206, which exhibits comparable efficacy with significantly less toxicity. To identify pathways synthetically lethal upon CDK12/13 degradation, phosphorylation pathway arrays were performed using cell lines treated with YJ1206. Interestingly, degradation or genetic knockdown of CDK12/13 led to activation of the AKT pathway. Targeting CDK12/13 for degradation, in conjunction with inhibiting the AKT pathway, resulted in a synthetic lethal effect in preclinical prostate cancer models.

INTRODUCTION

Prostate cancer is one of the leading causes of cancer-related deaths in men worldwide.[1] Patients with localized and advanced prostate tumors are sensitive to androgen deprivation therapies.[2–4] Although treatment with second-generation antiandrogens, such as enzalutamide and abiraterone, initially constrains tumors, most advanced patients ultimately relapse with metastatic castration-resistant prostate cancer (mCRPC)[5–7] and succumb to this disease.[8] Thus, there is an urgent need to develop therapeutic regimens to combat therapy resistance in castration-resistant prostate cancer (CRPC) and increase survival.

CDK12 and its paralog CDK13 belong to the transcriptional cyclin-dependent kinase (CDK) family of serine/threonine protein kinases and participate in overlapping cellular processes.[9] CDK12 and CDK13 play crucial roles in transcription elongation, DNA damage response (DDR), and maintenance of genomic stability.[10,11] Functionally, CDK12 and CDK13 act cooperatively in complex with cyclin K (CCNK) to phosphorylate serine 2 of the RNA polymerase II C-terminal domain (CTD), a modification that regulates transcription elongation, splicing, and cleavage and polyadenylation.[12] Depletion or loss of function of the CDK12/13/CCNK complex induces DNA damage through reduced expression of DDR genes[13,14] and inhibits cell proliferation in cancer cells.[15] Double knockdown of *CDK12* and *CDK13* augments the cytotoxicity versus knockdown of either gene alone.[16]

Emerging evidence suggests a significant involvement of CDK12 in various cancers. In prostate cancer, *CDK12* mutations occur in 5%–7% of patients with mCRPC.[17–19] The biallelic loss of *CDK12* leads to a unique genomic signature characterized by widespread focal tandem duplications.[17] CDK12 also modulates

© 2024 The Author(s). Published by Elsevier Inc.
This is an open access article under the CC BY license (http://creativecommons.org/licenses/by/4.0/).

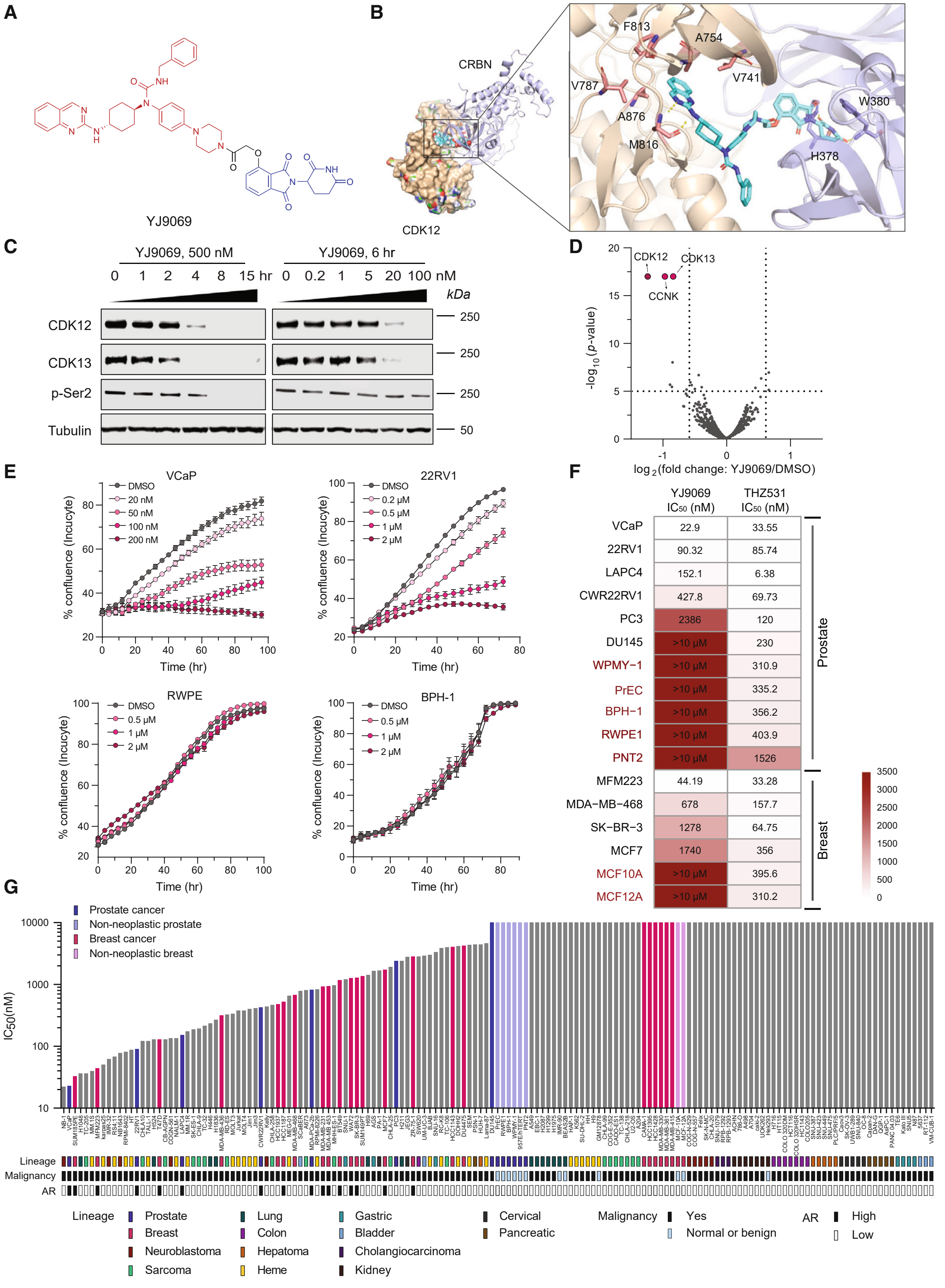

(legend on next page)

the growth of Ewing sarcoma driven by EWS-FLI1 fusion.[20] Loss-of-function mutations in ovarian cancer cause defects in multiple DNA repair pathways, which increase genomic instability.[21] Knockdown of the CDK12/CCNK complex sensitizes cells to chemotherapy in high-grade serous ovarian cancer.[22] In breast cancer, inhibition of CDK12/CDK13 results in deficiencies in DNA damage repair, promoting synergy with DNA-damaging chemotherapy.[16]

The protein serine/threonine kinase AKT, also termed protein kinase B, is another pivotal regulator of various cellular processes, including apoptosis, survival, proliferation, and metabolism.[23] The PI3K/AKT signaling pathway is implicated in the pathogenesis of numerous malignancies.[24] The AKT pathway is upregulated in 30%–60% of prostate cancers, particularly in CRPC and those with high Gleason scores.[25–27] Monotherapy with PI3K-AKT pathway inhibitors has shown minimal activity in clinical trials in prostate cancer; however, combination therapies targeting this pathway have demonstrated enhanced anti-tumor effects, suggesting the potential of strategic combination treatments.[28] Recently, the United States Food and Drug Administration (FDA) has approved the first AKT inhibitor, capivasertib, in combination with the estrogen receptor antagonist fulvestrant, for the treatment of hormone receptor-positive, HER2-negative breast cancer.[29] In addition, combining AKT inhibitors with inhibitors of androgen signaling increases anti-tumor effects in phosphatase and tensin homolog (PTEN) null tumor cell lines and tumor models of prostate cancer.[30] Moreover, combining the AKT inhibitor ipatasertib with abiraterone improves radiographical progression-free survival in mCRPC patients with PTEN protein loss compared to abiraterone alone.[31]

The development of CDK12/13 inhibitors, such as the irreversible covalent inhibitor THZ531[20,32,33] and the noncovalent inhibitor SR-4835,[16] has shown promise in inducing synthetic lethality in multiple cancer types.[32,34] However, these inhibitors failed in clinical trials due to toxicity.[35] Proteolysis targeting chimera (PROTAC) technology has emerged as a groundbreaking strategy in drug development for inducing protein degradation.[36,37] Recently, Jiang et al. developed the first CDK12 PROTAC degrader, BSJ-4-116, based on the covalent CDK12 inhibitor THZ531.[38] However, no preclinical studies have been reported for BSJ-4-116. Recently, we published a PROTAC degrader 7f,[39] which exhibited remarkable degradation of CDK12/13 *in vitro* and *in vivo*. In this current study, we refine compound 7f to a CDK12/13 degrader, YJ9069, with enhanced *in vitro* and *in vivo* activity. Based on YJ9069, we further developed YJ1206, an orally bioavailable CDK12/13 degrader. Both *in vitro* and *in vivo* data demonstrate a more potent and highly selective efficacy of YJ1206 compared to 7f and YJ9069 with an improved safety profile. Notably, degradation of CDK12/13 by YJ1206 induces AKT phosphorylation, revealing significant synergistic antiproliferative effects when combined with AKT inhibitors in prostate cancer models. Our data strongly suggest that YJ1206, as an oral CDK12/13 degrader, may serve as a promising candidate for combination therapy with AKT pathway inhibitors, offering an avenue for the effective treatment of prostate cancer.

RESULTS

YJ9069, a specific degrader of CDK12/13, exhibits preferential cytotoxicity in multiple cancers

Building upon compound 7f, we synthesized a degrader, YJ9069, which has enhanced activity against CDK12 and 13. The development of YJ9069 involved two pivotal structural modifications: (1) incorporation of a cyclic nicotinonitrile group in the molecule's head region to fit in the hydrophobic pocket and (2) changing the linker to thalidomide from a meta to an ortho substitution to amplify strain energy. These adjustments significantly improved YJ9069's binding affinity and docking scores with CDK12/cereblon and CDK13/cereblon complexes, outperforming compound 7f (Figures 1A, 1B, and S1A, Method S1). *In vitro*, YJ9069 displayed an enhanced degradation of both CDK12 and CDK13 proteins compared to 7f in a dose- and time-dependent manner (Figures 1C and S1B) and effectively inhibited phosphorylation of RNA polymerase II at serine 2 in VCaP and 22Rv1 cells (Figures 1C and S1C). Treatment with either free thalidomide alone, YJ9068 (the warhead of YJ9069), or an inactive epimer YJ1078 (Figures S1D) did not affect target protein levels or cancer cell survival and growth (Figures S1E and S1F). Competition of YJ9069 with thalidomide or YJ9068 rescued degradation of CDK12/13 targets (Figure S1F). Furthermore, pre-treatment of VCaP cells with proteasomal inhibitor carfilzomib, but not lysosomal inhibitor bafilomycin, hindered target protein degradation in a dose-dependent manner

Figure 1. YJ9069, a specific degrader of CDK12/13, exhibits preferential cytotoxicity in multiple cancers

(A) Chemical structure of CDK12/13 degrader YJ9069. CDK12/13 warhead is in red, and E3 ligand thalidomide is in blue.

(B) Docking model of YJ9069 (cyan sticks) with CDK12 (PDB: 6CKX) and CRBN (PDB: 6R1A) complex.

(C) Immunoblots of CDK12, CDK13, and pSer2 of RNAPII CTD in VCaP cells treated with YJ9069 at increasing concentrations or time durations. Tubulin is used as a loading control.

(D) Effects of YJ9069 (500 nM, 5 h) on the proteome of 22Rv1 cells. Data plotted Log2 of the fold change (FC) versus DMSO (dimethyl sulfoxide) control against −Log10 of the *p* value per protein (FDR, false discovery rate) from $n = 3$ independent experiments. All tests performed were two-tailed t test assuming equal variances. CDK12, CDK13, and CCNK are highlighted.

(E) Growth curves of VCaP, 22Rv1, RWPE, and BPH-1 cells upon treatment with increasing concentrations of YJ9069. Data are presented as mean ± standard deviation from $n = 3$ independent experiments.

(F) IC_{50} of YJ9069 and THZ531 in a panel of human-derived prostate and breast cell lines after 5 days of treatment. WPMY-1 is a human-derived prostate stromal cell line, while the others are human epithelial cell lines. All benign immortalized cell lines are highlighted as dark red. IC_{50} values are calculated from $n = 3$ independent experiments.

(G) IC_{50} of YJ9069 in a panel of human-derived cancer or normal cell lines after 5 days of treatment. AR, androgen receptor. IC_{50} values are calculated from $n = 3$ independent experiments.

See also Figure S1 and Method S1.

(Figure S1F), indicating that YJ9069 required the proteasome machinery for its action. Mass spectrometry-based proteomics analysis confirmed CDK12, CDK13, and CCNK as the only significantly downregulated proteins (Figure 1D).

In several prostate cancer cell lines, YJ9069 effectively inhibited cell growth as measured by IncuCyte in a dose-dependent manner while having no antiproliferative effect on benign or non-neoplastic prostate cells at parallel doses (Figures 1E, S1G, and S1H). Notably, treatment with YJ9069 disrupted PC310 organoid morphology and reduced cell viability at the noted concentrations (Figures S1I–S1K). We next compared the effects of the YJ9069 degrader to the CDK12/13 inhibitor, THZ531, on a series of prostate and breast cell lines (Figure 1F). In general, most prostate and breast cancer cell lines tested were sensitive to both THZ531 and YJ9069. By contrast, most benign immortalized cell lines (such as PrEC, WPMY-1, BPH-1, RWPE1, PNT2, MCF10A, and MCF12A) were relatively resistant to YJ9069 (half-maximal inhibitory concentration [IC_{50}] > 10 μM) as compared to THZ531. The wider *in vitro* therapeutic window of YJ9069 compared to THZ531 suggests a potential advantage of the degrader relative to the inhibitor approach. Cell viability screening was extended to a panel of 155 total normal and cancer cell lines from 14 distinct lineages. While normal and non-neoplastic cells were resistant to YJ9069 (IC_{50} > 10,000 nM), androgen receptor (AR)-positive prostate and breast cancer cells were preferentially sensitive (IC_{50} < 500 nM) (Figure 1G). Additionally, Ewing's sarcoma cell lines with the EWS-FLI1 fusion, such as TC-205, CHLA10, and CB-AGPN, demonstrated a higher sensitivity to YJ9069 compared to other sarcoma cell lines, which is in line with a previous report that THZ531 impairs DNA damage repair in an EWS/FLI-dependent manner in Ewing sarcoma.[20] We further performed bioinformatic analyses on the cell lines (Figure 1G) to identify the molecular signatures rendering sensitivity to YJ9069. As shown in Figures S1L and S1M, multiple signature pathways, including p53 transcription, transforming growth factor β signaling, as well as PI3K/AKT pathway, were upregulated, while tumor suppressor and DNA damage pathways were downregulated in the resistant cell lines. Collectively, these results show that YJ9069 is a specific PROTAC degrader of CDK12 and CDK13 with potent growth inhibitory effects in subsets of cancer cell lines.

CDK12/13 degradation leads to a gene-length-dependent elongation defect

Inhibition of CDK12 has previously been reported to selectively regulate the expression of long genes.[10–12,15] To determine whether our CDK12/13 degrader elicited similar effects, gene expression was profiled by RNA sequencing (RNA-seq) in VCaP cells treated with YJ9069 for 5 h. Notably, a significant correlation between gene length and gene expression was observed, with longer genes more likely to be downregulated with YJ9069 treatment (Figure 2A). To further define the relationship, downregulated genes were categorized into 4 quartiles based on the distribution of gene lengths (short [<12 kb], medium-short [12–31.5 kb], medium-long [31.5–78.4 kb], and long [>78.4 kb]). As shown in Figure 2B, long genes consistently exhibited the most pronounced transcriptional downregulation.

To elucidate the impact of CDK12/13 degradation on transient RNA synthesis, nascent transcript sequencing (EU-RNA-seq), a 5-ethynyl uridine (EU)-pulse labeling method,[40] was performed on cells treated with YJ9069. Changes in nascent RNA expression across protein-coding gene bodies were determined at different time points. Average meta-gene analysis revealed a notable increase in reads toward the transcription start sites (TSSs) and a distinct reduction toward the transcription end sites (TESs) for long genes in a time-dependent manner (Figure S2A). As time progressed, transcriptional activity diminished in downstream regions of the gene unit, while prominent activity persisted toward the TSS. This trend gradually attenuated from the medium-long to medium-short category and was completely absent in the short category (Figure S2A). Individual gene examples clearly displayed the accumulation of sequence tracks toward the 5′-end of long genes (*ATM* and *ATR*) starting from 2 h, whereas no discernible effects were observed on short genes (*NRAS* and *ERGIC3*), even after 12 h (Figure S2B).

The global effects of YJ9069 on gene expression were subsequently assessed in VCaP cells. Ingenuity pathway analysis revealed that CDK12/CDK13 degradation resulted in significant upregulation of genes associated with DDR, p53, and AKT-mTOR (mammalian target of rapamycin) pathways, while downregulated genes were involved in DNA repair and DNA double-strand break repair pathways (Figures S2C and S2D). A neutral comet assay confirmed the induction of DNA damage by YJ9069 and THZ531, as evidenced by a significant increase in the tail moment (Figures 2C and S2I). Furthermore, real-time qPCR demonstrated a significant decrease in DDR gene expression following YJ9069 treatment in both VCaP and 22Rv1 cells, which was comparable to THZ531 (Figures 2D, S2J, and S2K). Cell-cycle analysis revealed a dose-dependent subG1 arrest in VCaP cells upon treatment with YJ9069 (Figure 2E). Taken together, these results indicate that CDK12/13 degradation by YJ9069 leads to an elongation defect primarily affecting genes within the long-length categories, and CDK12/13-regulated genes play a primary role in several cellular pathways regulating DNA damage and repair.

YJ9069 suppresses tumor growth in multiple *in vivo* prostate cancer models

Given the remarkable sensitivity of prostate cancer cells (IC_{50} < 200 nM) to YJ9069 (Figure 3A), we proceeded to evaluate its therapeutic efficacy in animal models of advanced prostate cancer. The VCaP castration-resistant prostate cancer model (VCaP-CRPC) was first employed to assess the pharmacodynamics on day 5 (PD5) of YJ9069. Following treatment with YJ9069, a significant decrease in CDK12 and CDK13 proteins and an increase in cleaved poly (ADP-ribose) polymerase (PARP) within tumors were observed compared to vehicle controls (Figure 3B). Immunohistochemistry (IHC) assays further demonstrated robust reductions in CDK12 levels and increased cleaved PARP (c-PARP) and TUNEL signals within tumors (Figure 3C). Moreover, YJ9069 treatment resulted in a marked decrease in DDR gene expression, including *ATM*, *ATR*, and *BRCA1*, within treated tumors (Figure S3A). Upon continuous treatment for 18 days, YJ9069 exhibited potent inhibition of

CellPress
OPEN ACCESS

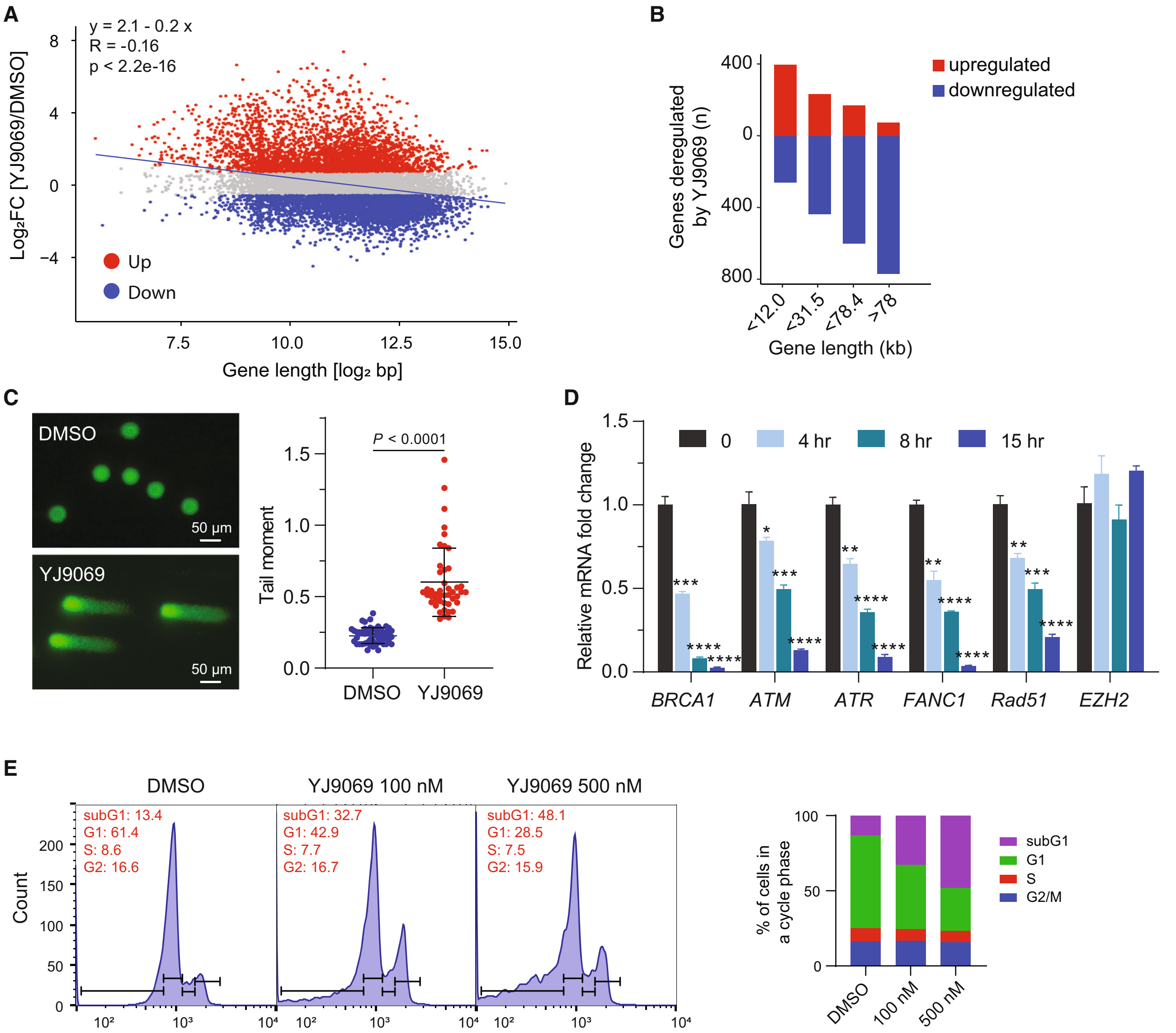

Figure 2. CDK12/13 degradation leads to gene-length-dependent elongation defects in transcription, loss of long gene expression, DNA damage, and cell-cycle arrest

(A) Scatterplot showing Log2 fold changes in gene expression vs. Log2 scale in gene length for each protein-coding gene in VCaP cells following treatment with YJ9069 at 500 nM for 12 h ($p < 2.2e{-16}$, F-test). Differentially expressed genes are indicated (FDR < 0.05 and Log2 FC > 1) from $n = 3$ independent experiments.

(B) Number of genes up- or downregulated relative to gene length. The genome was ranked from smaller to longer genes and fractioned into 4 groups containing the same number of genes.

(C) Representative images of comet assay in VCaP cells after treatment with vehicle or YJ9069 (100 nM) for 12 h (scale, 50 μm) (left panel), and quantification of tail moments (right panel). Boxplots represent interquartile ranges; horizontal bars denote the median. For each condition, 50 cells were analyzed.

(D) Analysis of indicated gene expression by real-time qPCR at 4 h, 8 h, and 15 h with YJ9069 (50 nM) or vehicle in VCaP cells. Data are presented as mean values ± standard deviation of triplicate experiments. $*p < 0.05$, $**p < 0.01$, $***p < 0.001$ by t test.

(E) Cell-cycle analyses by flow cytometry of VCaP cells treated with 100 nM and 500 nM of YJ9069 for 15 h. The bar graph (right) demonstrates the quantification of the cell-cycle phase data.

See also Figure S2.

tumor growth (Figures 3D and 3E), inducing tumor regression exceeding 50% in 73% of treated animals (Figure 3F).

In addition to the cell-derived xenograft (CDX) models, we further evaluated the *in vivo* efficacy of YJ9069 in WA74 and PC310, two AR-positive patient-derived xenograft (PDX) models. While YJ9069 remarkably suppressed tumor growth in both models (Figures 3I–3K and 3N–3P), it induced the most potent anti-tumor effect in the WA74 PDX model, with regression in all animals after 30 mg/kg, 3 times/week treatments for 21 days (Figure 3K). Hematoxylin and eosin (H&E) staining demonstrated

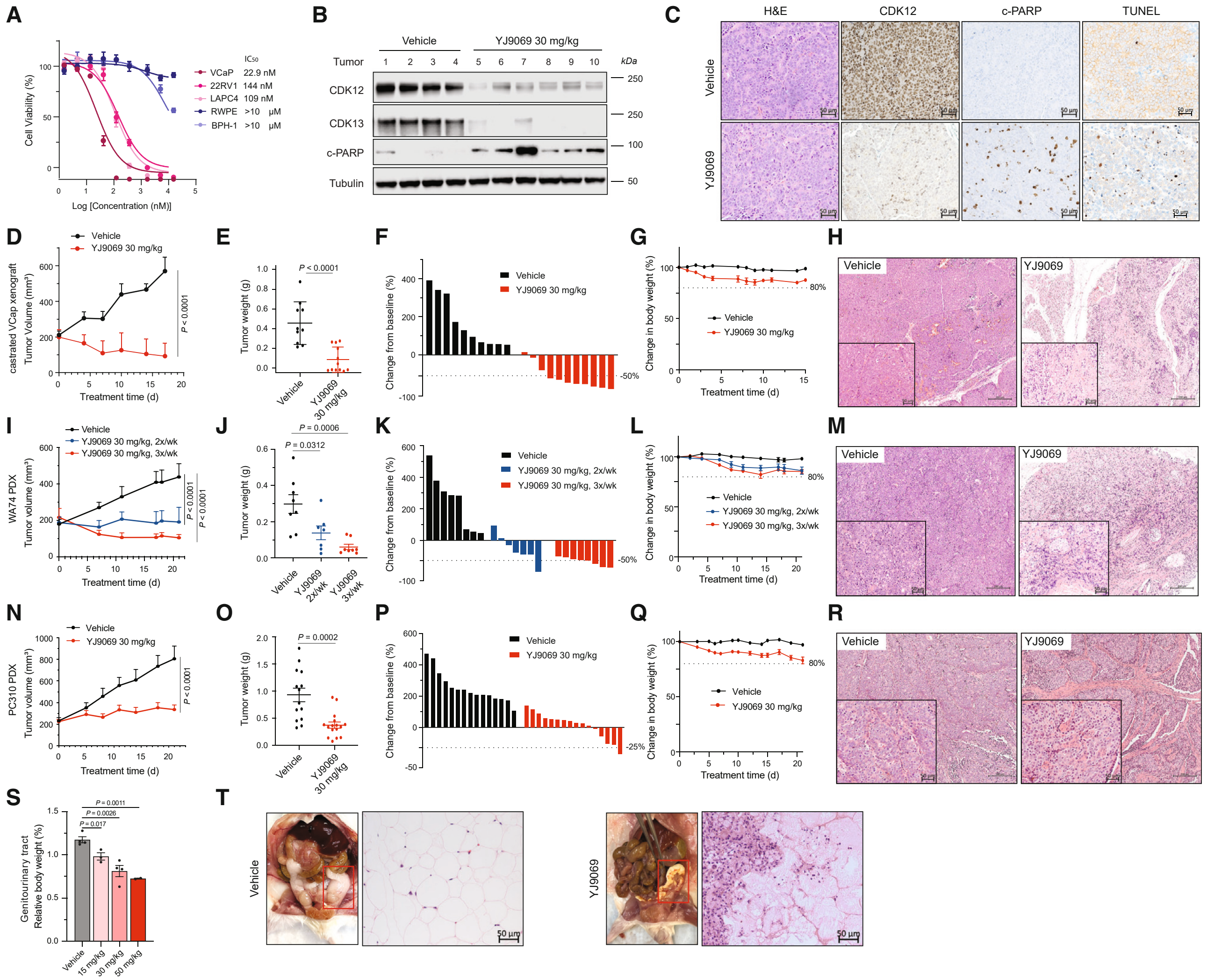

Figure 3. YJ9069 suppresses tumor growth in multiple *in vivo* models of castration-resistant prostate cancer

(A) Dose-response curves and IC_{50} of cells treated with YJ9069. Data are presented as mean ± standard deviation (*n* = 3) from one of three independent experiments.

(B) Immunoblot of CDK12, CDK13, and cleaved PARP for castrated VCaP *in vivo* xenografted tumors after 5 days treatment with YJ9069 (i.v., 30 mg/kg, 3x/week). Tubulin is the loading control.

(C) Representative H&E staining and immunohistochemistry for CDK12, cleaved PARP, and TUNEL from the PD5 study in (B) (scale, 50 μm).

(D) Tumor volume (measured twice weekly using calipers) in the castrated VCaP model treated with YJ9069 (i.v., 30 mg/kg, 3x/week) (two-sided t test). Data are mean ± standard error of the mean (SEM) (vehicle: *n* = 10; YJ9069: *n* = 11).

(E) Tumor weights for vehicle and YJ9069 groups from castrated VCaP study (two-sided t test). Data are presented as mean ± SEM.

(F) Waterfall plot depicting the change in tumor volume after 18 days of treatment. Response evaluation criteria in solid tumors (RECIST) was used to stratify tumors: progressive disease (PD), at least a 20% increase in tumor size; stable disease (SD), an increase of <20% to a decrease of <30%; partial response (PR), at least a 30% decrease. The vehicle group has 100% PD; the YJ9069 group has 18% SD and 82% PR.

(G) Percent body weight measurement showing the effect of vehicle and YJ9069 in the castrated VCaP model throughout the treatment period. Data are presented as mean ± SEM.

(H) Representative H&E staining for vehicle- and YJ9069-treated tumors from castrated VCaP xenograft at the endpoint (scale, 200 μm). The inset scale, 50 μm.

(I–M) as in (D–H), except in the WA74 patient-derived xenograft (PDX) model with YJ9069 treatment (i.v., 30 mg/kg, 2x/week or 3x/week) (*n* = 8 per condition). In the waterfall plot, the YJ9069 2x/week group has 14% PD, 43% SD, and 43% PR; the YJ9069 3x/week group has 100% PR.

(N–R) as in (D–H), except in the PC310 PDX model with YJ9069 (i.v., 30 mg/kg, 3x/week). In the waterfall plot, the YJ9069 group has 63% PD, 31% SD, and 6% PR.

(S) Genitourinary tract measurement for vehicle and YJ9069 groups at noted doses in CD-1 male mouse (two-sided t test). Data are presented as mean ± SEM (*n* = 4, biological replicates).

(T) Representative photographs with matched H&E staining of the genitourinary region from vehicle and YJ9069 (i.v., 30 mg/kg, 3x/week) groups in CD-1 male mouse (scale, 50 μm).

See also Figure S3.

CellPress
OPEN ACCESS

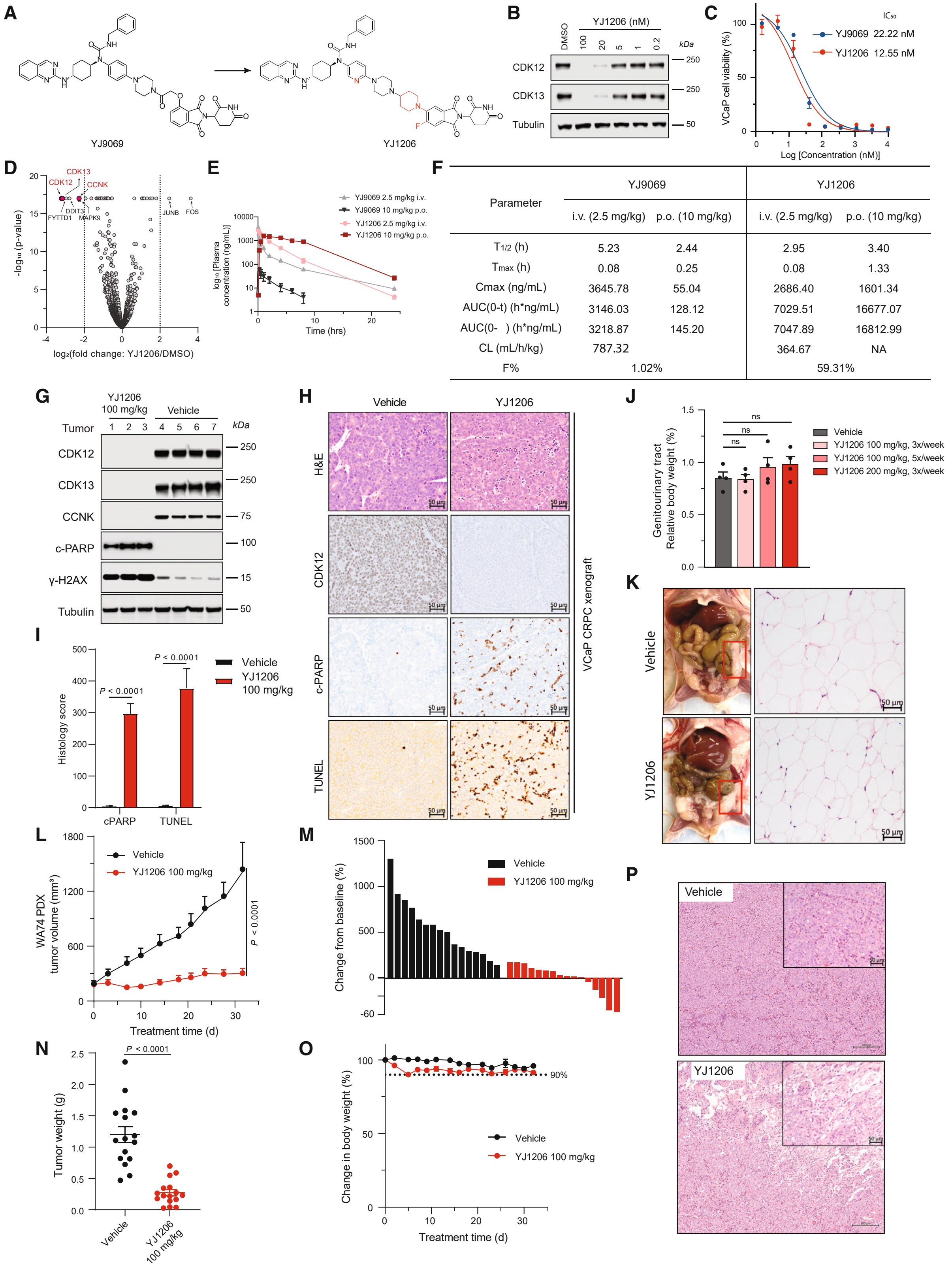

Parameter	YJ9069		YJ1206	
	i.v. (2.5 mg/kg)	p.o. (10 mg/kg)	i.v. (2.5 mg/kg)	p.o. (10 mg/kg)
$T_{1/2}$ (h)	5.23	2.44	2.95	3.40
T_{max} (h)	0.08	0.25	0.08	1.33
Cmax (ng/mL)	3645.78	55.04	2686.40	1601.34
AUC(0-t) (h*ng/mL)	3146.03	128.12	7029.51	16677.07
AUC(0-) (h*ng/mL)	3218.87	145.20	7047.89	16812.99
CL (mL/h/kg)	787.32		364.67	NA
F%	1.02%		59.31%	

(legend on next page)

clear evidence of morphological changes associated with tumor regression in all tumors treated with YJ9069, including varying degrees of hyalinization, remnant tumor nodules in areas of degenerative cells and dystrophic calcification, and bisecting tumor collagenization bands (Figures 3H, 3M, 3R). This *in situ* data confirmed a robust tumor response to YJ9069.

In all models, however, we observed less than 20% body weight loss in the treated animals (Figures 3G, 3L, and 3Q). We, therefore, evaluated the toxicity of YJ9069 in CD-1 male and female immune-competent mice. In the male group, animals treated with doses of 30 mg/kg and 50 mg/kg experienced a 10% body weight loss (Figure S3B). CDK12 degradation in the liver and spleen of these animals indicated on-target effects of YJ9069 (Figure S3C). Histopathological evaluation revealed no tissue-modulatory effects or changes in organ weights in the harvested liver, spleen, kidney, or testes (Figure S3D). However, treated mice exhibited a decrease in genitourinary (GU) weight (Figure 3S) and the presence of gonadal fat overgrowth adjacent to the GU region at both 30 mg/kg and 50 mg/kg doses (Figure 3T). Further examination of these fatty areas revealed focal areas of fat necrosis (Figure 3T). Notably, no significant histological changes were noted in the liver, prostate, or spleen (Figure S3E). Additionally, serum chemistry and complete blood analyses showed no significant changes in the YJ9069-treated male mice (Figure S3F-G). In the female mice, no significant toxicity was observed following treatment with YJ9069 (Figures S3H–S3K). Collectively, these data show that YJ9069 administered intravenously causes mild to intermediate toxicity *in vivo*, highlighting the need for the development of an improved compound to enhance its safety profile.

Development of an orally bioavailable CDK12/13 degrader YJ1206

To mitigate the toxicity associated with YJ9069, we further optimized the linker to improve the pharmacokinetic (PK) properties (Figures S4A–S4C). Moving the linker from the C4 (YJ9069) to C5 position in thalidomide (YJ1090 and YJ1094) retained the CDK12/13 degradation abilities. In contrast, the linear unsaturated, benzene, or pyrrolidine linkers (YJ1114, YJ1130, YJ1096) significantly decreased CDK12/13 degradation and cell viability. Compounds YJ1105, YJ1205, and YJ1206, with a piperidine linker, showed potent degradation effects and inhibited VCaP cell viability, with IC_{50} values below 20 nM. Subsequently, YJ1105, YJ1205, and YJ1206 were selected for PK tests in Sprague-Dawley rats. As shown in Figures S4D and S4E, YJ1206 demonstrated the best PK properties, with a bioavailability over 39% by oral gavage. Thus, we selected YJ1206 (Figure 4A), the orally bioavailable CDK12/13 degrader, for subsequent biological studies.

In vitro, YJ1206 effectively degraded CDK12 and CDK13 in a dose-dependent manner (Figure 4B) and displayed a comparable potency as YJ9069 (IC_{50} = 12.55 nM vs. 22.22 nM for YJ9069) in VCaP cells (Figure 4C). Global proteomic analyses in 22Rv1 affirmed the high selectivity of YJ1206, with CDK12, CDK13, and CCNK emerging as the most significantly degraded proteins (Figure 4D). We also identified a few additional proteins, including FYTTD1, DDIT3, MAPK9, and CKD9, that were decreased with YJ1206 treatment. To evaluate these potential off-target degradative effects, we treated 22Rv1 cells with YJ1206 at different time points. Western blot analysis demonstrated that while YJ1206 significantly degraded CDK12 and CDK13 starting at 2 h, the other proteins were not decreased until 6 h (Figure S4F, suggesting that these proteins may not be direct targets of YJ1206. No discernible alterations in the abundance of other CDK family members were observed (Figures S4G and S4H). Immunoblot analyses also detected no changes in other neo-substrates[41] in VCaP cells following YJ1206 treatment at different time points (Figure S4I).

Similar to YJ9069, a significant correlation between gene length and gene expression was observed from RNA-seq

Figure 4. Development of YJ1206, an orally bioavailable CDK12/13 degrader analog of YJ9069
(A) Chemical design of the oral CDK12/13 degrader YJ1206.
(B) Immunoblots of CDK12 and CDK13 in VCaP cells treated with YJ1206 at increasing concentrations for 4 h. Tubulin is used as a loading control.
(C) Dose-response curves and IC_{50} of VCaP cells treated with YJ9069 and YJ1206 for 5 days. Data are presented as mean ± standard deviation from n = 3 independent experiments.
(D) Effects of YJ1206 (500 nM, 8 h) on the proteome of 22Rv1 cells. Data plotted Log2 of the fold change (FC) versus DMSO against −Log10 of the p value per protein (FDR, false discovery rate) from n = 3 independent experiments. All tests performed were two-tailed t test assuming equal variances. CDK12, CDK13, and CCNK are highlighted in red.
(E) Plasma concentration-time curve of YJ1206 with intravenous (i.v., 2.5 mg/kg) and oral administration (p.o., 10 mg/kg) injection in CD-1 mice (n = 3 per condition).
(F) Pharmacokinetic profile of YJ9069 and YJ1206 following intravenous (i.v., 2.5 mg/kg) and oral (p.o., 10 mg/kg) injection in CD-1 mouse.
(G) Immunoblots of CDK12, CDK13, CCNK, cleaved PARP, and γ-H2AX for the castrated VCaP tumors after 5 days treatment with YJ1206 (p.o., 100 mg/kg, 3x/week). Tubulin is the loading control.
(H) Representative H&E staining and immunohistochemistry of CDK12, cleaved PARP, and TUNEL for PD5 study in panel G (scale, 50 μm).
(I) Histology score for immunohistochemistry of cleaved PARP and TUNEL in (G) (two-sided t test). Data are presented as mean ± standard deviation.
(J) Genitourinary tract measurement of the vehicle and YJ1206 group at noted doses in CD-1 male mouse (two-sided t test). Data are presented as mean ± SEM (n = 4, biological replicates).
(K) Representative photographs with matched H&E staining of the genitourinary region from vehicle and YJ1206 group in CD-1 male mouse (scale, 50 μm).
(L) Tumor volume (measured twice weekly using calipers) in the WA74 PDX model with YJ1206 (p.o., 100 mg/kg, 3x/week) (two-sided t test). Data are mean ± SEM (n = 16 per condition).
(M) Waterfall plot depicting the change in tumor volume. The evaluation criteria are the same as Figure 3F. The YJ1206 group has 56% PD, 25% SD, and 19% PR.
(N) Tumor weights for vehicle and YJ1206 groups from WA74 PDX study (two-sided t test). Data are presented as mean ± SEM.
(O) Percent body weight measurement showing the effect of vehicle and YJ1206 in WA74 PDX study throughout the treatment period. Data are presented as mean ± SEM.
(P) Representative H&E staining for WA74 PDX tumors at the endpoint treatment (scale, 200 μm). The inset scale, 50 μm
See also Figures S4 and S5 and Method S1.

performed in VCaP cells, with longer genes more likely to be downregulated after YJ1206 treatment (Figure S2E). Real-time qPCR on the nascent RNA from VCaP cells also showed that YJ1206 treatment significantly increased the transcripts at the TSSs, while a distinct reduction toward the TESs of the long genes *ATM* and *ATR* was observed in a time-dependent manner (Figure S2H). However, the short genes *NRAS* and *ERGIC3* were not significantly affected by YJ1206 at either site. Enrichment analysis on RNA-seq data also demonstrated that DDR, p53, and AKT-mTOR pathways were upregulated with YJ1206 treatment, while DNA repair and DNA double-strand break repair pathways were downregulated (Figures S2F and S2G). Decreased DDR gene expression by YJ1206 was confirmed in VCaP cells by qPCR (Figure S2L).

Prior to conducting *in vivo* efficacy studies, we first conducted a PK experiment in CD-1 mice to confirm oral bioavailability. Similar to rats, we observed significantly enhanced PK properties compared to YJ9069, including a higher C_{max} of 1601.34 ng/mL, area under the curve (AUC) of 16677.07 h*ng/mL, and an oral bioavailability of 59.31% (Figures 4E and 4F). In a PD5 study of YJ1206 via oral administration in the VCaP-CRPC model, a complete abrogation of CDK12, CDK13, and CCNK was observed in tumors, concomitant with increases of c-PARP and γH2AX (Figure 4G). This effect was further confirmed through histopathological H&E assessments, revealing a noticeable increase in apoptotic bodies, as validated by enhanced c-PARP immunohistochemistry (IHC) and TUNEL staining on spatially corresponding areas in serial sections from the same representative tumor sample (Figures 4H and 4I). Notably, while YJ1206 also degraded CDK12/13 proteins in host organs such as the liver, kidney, and prostate, there was no significant increase in c-PARP or TUNEL staining (Figures S5A and S5B), indicating minimal toxicity in these normal tissues. Prolonged treatment with YJ1206 in both male and female immuno-competent CD-1 mice exhibited no evidence of toxicity (Figures S5C–S5M). Notably, no differences were observed in GU weight (Figure 4J) or the presence of gonadal fat adjacent to the GU region (Figure 4K).

Oral administration of YJ1206 in the WA74 PDX model significantly suppressed tumor growth, resulting in regression in over 31% of treated tumors, while no significant changes were noted in animal body weights (Figures 4L–4O). Additionally, histopathological H&E evaluation of treated samples revealed evidence of tumor response to YJ1206, characterized by areas of hyalinization and remnant tumor nodules, among other indicators (Figure 4P). Taken together, these results highlight the potent anti-tumor efficacy and improved safety profile of YJ1206, suggesting promising therapeutic potential as an orally bioavailable CDK12/13 degrader.

YJ1206 combined with an AKT inhibitor triggers a synergistic effect *in vitro*

To investigate signaling pathways that are altered following CDK12/13 degradation, VCaP and 22Rv1 cells were first treated with YJ1206 for 15 h, and cell extracts were then subjected to a phosphorylation pathway profiling array. Remarkably, the AKT pathway exhibited the most significant elevation in signal in both cell lines, while no discernible changes were observed in the Janus kinase/signal transducers and activators of transcription (JAK/STAT) and nuclear factor κB pathways (Figures 5A, 5B, S6A, and S6B). Subsequently, we assessed the expression levels of pAKT (S473) protein in both VCaP and 22Rv1 cells via western blotting to validate the array data. Knockdown of *CDK12* and/or *CDK13* genes by small interfering RNA (siRNA) resulted in substantial reduction of the target proteins (Figures 5C and S6C). Intriguingly, while knockdown of either CDK12 or CDK13 led to increased pAKT at serine 473, knockdown of both CDK12/13 markedly enhanced pAKT (S473), along with its direct substrate PRAS40 and downstream effector S6 (Figures 5C and S6C). These results were further confirmed by immunoblotting in both VCaP and 22Rv1 cells upon CDK12/13 degradation induced by YJ1206 in a time- and dose-dependent manner. Notably, total AKT and PRAS40 levels remained unchanged (Figures 5C and S6C).

Emerging evidence suggests that phosphorylation-induced activation of oncogenes, such as PI3K/AKT[42] and extracellular signal-regulated kinase (ERK),[43] induces chemoresistance in cancer cells. Interestingly, gene set enrichment analysis on the cell viability profiling of YJ9069 also showed that the PI3K-AKT pathway was enriched in resistant cell lines (Figures S1L and S1M). We, therefore, hypothesized that combined YJ1206 and AKT inhibitor treatment would exert a synthetical lethal effect. Uprosertib was selected since it is an orally bioavailable AKT inhibitor that has been evaluated in several clinical trials.[44,45] While genetic knockdown of both CDK12/13 by siRNA attenuated cell growth as measured by IncuCyte, combinatorial treatment with uprosertib demonstrated enhanced efficacy (Figures 5D, 5E, S6D, and S6E). Knockdown of either CDK12 or CDK13 had no discernible effects on cell proliferation, underscoring the necessity of inhibiting both CDK12/13 function to suppress cancer cell growth.[16] Similarly, a combination of YJ1206 with other AKT inhibitors, including uprosertib, capivasertib, and MK2206, displayed a significant synergistic effect in 22Rv1 and VCaP cells (Figures 5F, 5G, and S6F–S6I). Combination treatment with the CDK12/13 inhibitor THZ531 and AKT inhibitors also resulted in enhanced inhibition of cell growth (Figures S6J–S6K). Notably, significant synergy was also achieved in PC310 PDX organoids upon combinatorial treatment of YJ1206 with uprosertib or capivasertib, yielding a synergy score of 19.664 and 18.755, respectively (Figures 5H and 5I). Representative images in Figure S6L show that YJ1206 or capivasertib alone attenuated the growth of PC310 PDX organoids, while the combination completely inhibited organoid growth. Collectively, these findings demonstrate that CDK12/13 degradation induces synthetic lethality *in vitro* when coupled with AKT pathway inhibition.

Combination oral CDK12/13 degrader YJ1206 and AKT inhibitor treatment suppresses tumor growth *in vivo*

In vivo, a PD5 study demonstrated complete degradation of CDK12/13 targets with both YJ1206 and the combinatorial regimen (YJ1206 + uprosertib) in the VCaP-CRPC model (Figure S7A). Notably, YJ1206 markedly increased pAKT (S473) and p-S6 levels in tumors compared to the vehicle group. Combinatorial treatment with uprosertib led to a reduction in the phosphorylation of downstream protein S6 (Figure S7A). To be noted, uprosertib, an ATP-competitive AKT inhibitor, increased

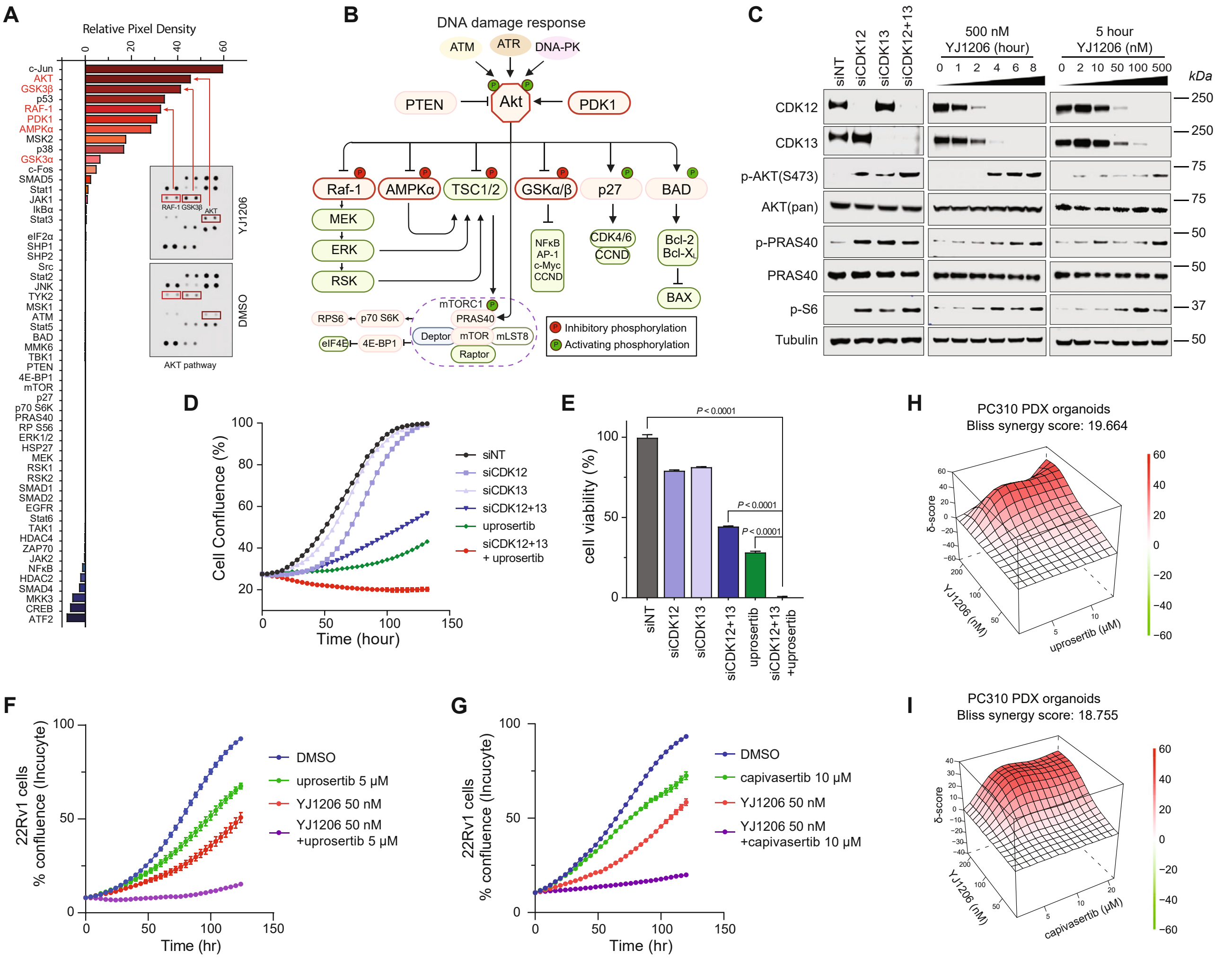

Figure 5. CDK12/13 degradation induces synthetic lethality in conjunction with AKT pathway inhibition *in vitro*

(A) Human phosphorylation pathway profiling array analysis of VCaP cells treated with YJ1206 (500 nM) for 15 h.

(B) The PI3K/AKT signaling pathway. The top phosphorylated proteins identified in Figure 5A are highlighted in red.

(C) Immunoblot of the noted proteins in VCaP cells treated with siRNA targeting CDK12 and/or CDK13 or YJ1206 at increasing concentrations and time durations. Tubulin is the loading control probed on all immunoblots.

(D) Real-time growth curves of VCaP cells upon treatment with siCDK12/13 and/or uprosertib. Data are presented as mean ± standard deviation from three independent experiments.

(E) Cell viability of VCaP cells treated with siCDK12/13 and/or uprosertib by CellTiter-Glo assay (two-sided t test, $n = 3$ independent experiments).

(F and G) Real-time growth curves of 22Rv1 cells upon treatment with YJ1206 and/or uprosertib/capivasertib. Data are presented as mean ± standard deviation from $n = 3$ independent experiments.

(H and I) PC310 PDX organoids were treated with YJ1206 and/or uprosertib/capivasertib at varied concentrations to determine the effect on cell growth and drug synergism, with assessments using the Bliss independence method. Red peaks in the 3D plots denote synergy, and the average synergy scores are noted above the plots.

See also Figure S6.

feedback phosphorylation of AKT (S473) itself as previously reported.[46] Furthermore, DDR gene expression was significantly downregulated in both the YJ1206 and combinatorial treatment groups (Figure S7B). Histological analysis of tumors confirmed decreased CDK12 levels and elevated c-PARP and TUNEL signals in both the YJ1206 and combinatorial treatment groups (Figures S7C and S7D). The increased apoptosis resulting in tumor regression was also evident in the H&E-stained slides, where robust tumor regression was observed in tumor tissues from YJ1206- and YJ1206 + uprosertib-treated mice (Figure S7C).

To assess the clinical potential of YJ1206 combined with uprosertib, we evaluated the anti-tumor efficacy of the combination regimen in both castrated VCaP and 22Rv1 CDX models. While either YJ1206 or uprosertib alone exhibited moderate anti-tumor efficacy in both models, the combination of YJ1206 with uprosertib dramatically suppressed tumor burden, consistent with

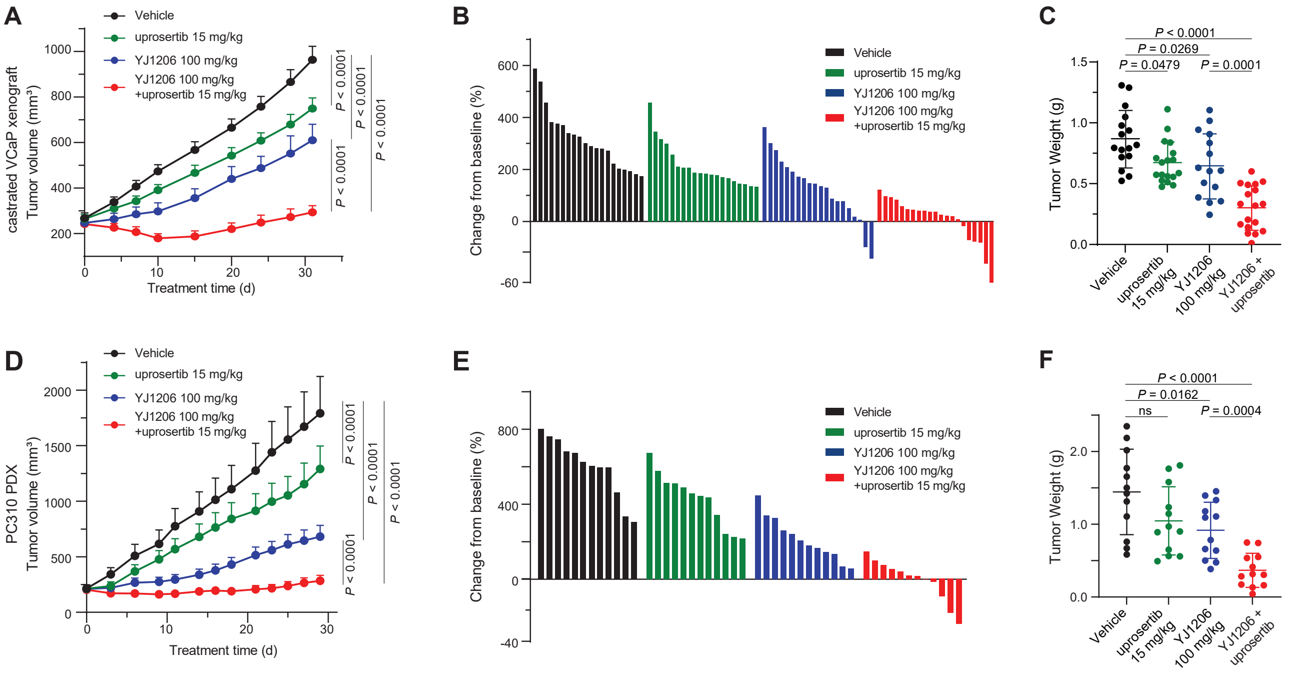

Figure 6. The combination regimen of CDK12/13 degraders with AKT inhibitors suppresses tumor growth *in vivo*

(A) Tumor volume in castrated VCaP xenograft model with YJ1206 (p.o., 100 mg/kg, 3x/week) alone or combined with uprosertib (p.o., 15 mg/kg, 5x/week) treatment (n = 20 per condition; two-sided t test). Data are mean ± SEM.

(B) Waterfall plot depicting the change in tumor volume after 31 days of treatment. The evaluation criteria are the same as Figure 3F. The vehicle and uprosertib groups have 100% PD; the YJ1206 group has 80% PD, 15% SD, and 5% PR; and the YJ1206 + uprosertib group has 62% PD, 24% SD, and 14% PR.

(C) Tumor weights from VCaP-CRPC xenograft study (two-sided t test). Data are presented as mean ± SEM.

(D–F) same as in (A–C), except in PC310 PDX (n = 12 per condition). In the waterfall plot, the vehicle, uprosertib, and YJ1206 groups have 100% PD; the YJ1206 + uprosertib group has 50% PD and 50% SD.

See also Figure S7.

our *in vitro* data (Figures 6A–6C, S7F, S7H–S7J, and S7L). We further investigated the efficacy of YJ1206 and/or uprosertib in the PC310 PDX model. The combinatorial regimen almost completely suppressed tumor growth in this PDX model (Figures 6D–6F and S7M), whereas either YJ1206 or uprosertib alone led to only moderate or mild tumor inhibition. Remarkedly, YJ1206 plus uprosertib treatment was well tolerated, as evidenced by the absence of significant body weight loss compared to the YJ1206-alone cohort in this experimental setting (Figures S7E, S7K, and S7N). No adverse effects in normal tissues were observed upon necropsy during this study (Figure S7G).

YJ1206 demonstrated extensive histopathological regression in both models as a single agent and when combined with uprosertib. Intriguingly, while treatment with uprosertib alone demonstrated mild tissue-modulatory effects, characterized by focal areas of hyalinization and collagen bundles within the tumor tissues, YJ1206 induced massive tumor regression, manifested by broad bands of collagenization, areas of hyalinization, and islands of degenerative cells (Figures S7F, S7L, and S7M). This observation was further accentuated in the combinatorial group, where viable tumor areas regressed but were compensated by the presence of necrosis and collagenization in tumor-free areas, likely contributing to the grossly observed tumor volume. Collectively, these results robustly support the synergistic anti-tumoral effects of YJ1206 and AKT inhibitors, both *in vitro* and *in vivo*, underscoring their potential as a combined therapeutic strategy for advanced prostate cancer management.

DISCUSSION

In this study, we introduce YJ9069, a highly specific PROTAC degrader, targeting the CDK12/13/CCNK complex, demonstrating cytotoxicity at nanomolar concentrations across a spectrum of cancers. Specifically, YJ9069 rapidly triggers gene-length-dependent transcriptional elongation defects leading to profound inhibition of cell proliferation, cell-cycle arrest, and apoptosis. Notably, compared to the CDK12/13 inhibitor THZ531, the CDK12/13 degrader YJ9069 effectively inhibited cell proliferation in subsets of prostate and breast cancer cell lines preferentially over benign immortalized cells. The wider *in vitro* therapeutic window of YJ9069 compared to THZ531 suggests a potential advantage of CDK12/13 degraders for clinical development relative to inhibitors. Pharmacological inhibition of CDK12/13 profoundly attenuates *in vitro* proliferation of prostate cancer cells and reduces tumor burden in subcutaneous mouse xenograft models. The critical dependency of prostate cancer cells on CDK12 and CDK13 for proliferation was further

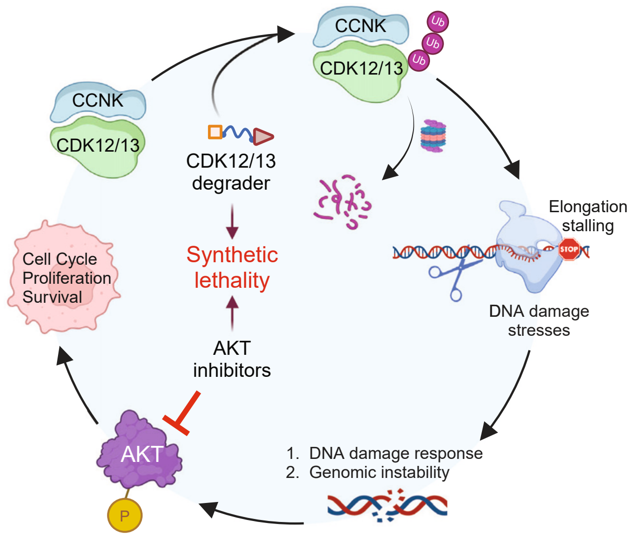

Figure 7. Mechanism of action of CDK12/13 degrader-induced AKT phosphorylation in prostate cancer

The CDK12/13 degrader inhibits Ser2 phosphorylation on RNAPII, disrupting gene expression and leading to DNA damage and instability. This DNA damage triggers AKT phosphorylation, promoting synthetic lethality with AKT inhibition.

recently corroborated by CRISPR-mediated gene editing.[47] *In vivo*, YJ9069 treatment leads to marked tumor regression in various CDX and PDX prostate cancer models. Nevertheless, moderate toxicity following YJ9069 treatment by intravenous injection was observed in multiple mouse models. Hence, based on YJ9069, we further designed and developed an orally bioavailable CDK12/13 degrader, YJ1206, which exhibits a comparable efficacy. The orally available degrader, YJ1206, exhibits significantly improved pharmacokinetic and pharmacodynamic properties, including a more gradual absorption, relatively lower maximum plasma concentration, and greatly extended dose window, which lead to a significant decrease in toxicity. Overall, our results show that YJ1206 is well tolerated in mice with no evident toxicity.

Mechanistically, YJ1206 induces a potent anticancer response by eliciting cellular stress and significantly altering the DDR (Figure 7). Its primary mechanism of action involves inhibition of serine 2 phosphorylation of the RNA polymerase II CTD by CDK12/13, disrupting the transcriptional machinery. This disruption is particularly detrimental in cancers harboring functional inactivating mutations in CDK12, such as ovarian and prostate cancers, where compromised DDR pathways lead to genomic instability.[17–19] In agreement with previous observations, our data find that degradation of CDK12/13 stalls transcriptional elongation, attenuates expression of key DDR genes, and leads to DNA lesion accumulation and genomic instability. Notably, these effects were also manifest in orthotopic PDX models of therapy-resistant prostate cancer, where YJ1206 increases DNA damage, induces apoptosis, and promotes tumor regression.

Interestingly, we found that degradation or genetic knockdown of CDK12/13 led to a significant elevation in AKT phosphorylation at serine 473. The AKT pathway represents one of the most frequently dysregulated pathways found in human cancer,[48,49] and aberrant activation of this pathway is associated with drug resistance and cancer progression.[50–52] This finding suggests a potential synthetic lethality approach, wherein targeting both CDK12/13 and the PI3K/AKT signaling pathway could yield substantial therapeutic benefits for prostate cancer. Indeed, our study demonstrates compelling synergistic efficacy, both *in vitro* and *in vivo*, of YJ1206 in combination with uprosertib or capivasertib, offering a promising avenue for the treatment of advanced CRPC with two orally bioavailable drugs. Notably, a subset of our experimental models achieved complete disease remission without any detectable toxicity, highlighting the clinical significance of this therapeutic approach.

It is well established that AKT is directly involved in regulating DDR signaling.[53] While further investigations are warranted to delineate the precise mechanisms underlying how CDK12/13 loss induces AKT phosphorylation by impairing the DNA damage repair machinery, the translational implications of our study are significant. YJ1206 represents a promising orally bioavailable CDK12/13 degrader developed as a therapeutic agent for cancer treatment. Given the recent FDA approval of the AKT inhibitor capivasertib for breast cancer, the promising outcomes of our research underscore the potential for conducting clinical trials in advanced prostate cancer patients.

Limitations of the study

In this study, we have developed an orally bioavailable CDK12/13 degrader (YJ1206) that is efficacious and exhibits minimal toxicity in preclinical models of advanced prostate cancer. Whether this therapeutic window can be maintained in human clinical studies will be determined in the near future. We also demonstrate that CDK12/13 degradation induces activation of the AKT pathway, the precise mechanism of which has yet to be defined. YJ1026 combined with an AKT inhibitor exhibited a synergistic effect. Potential toxicities of such a combination regimen have not been evaluated in immune-competent models.

RESOURCE AVAILABILITY

Lead contact

Further information and requests for resources should be directed to and will be fulfilled by the lead contact, Arul M. Chinnaiyan (arul@med.umich.edu).

Materials availability

All materials used in this paper are available from the lead contact upon request.

Data and code availability

RNA-seq and EU sequencing data have been deposited in the NCBI GEO repository, accession numbers GEO: GSE262119 and GEO: GSE262120, respectively. This paper does not report original code. Any additional information required to reanalyze the data reported in this work is available from the lead contact upon request.

ACKNOWLEDGMENTS

We thank Lisa McMurry, Amanda Miller, Christine Caldwell-Smith, Victoria Zeng, Grafton Ervine, and Shuqin Li from the Michigan Center for Translational Pathology at the University of Michigan. This work was funded by the Ministry

of Science and Technology of China (2023YFF1205104, to K.D.), a National Cancer Institute (NCI) Prostate Specialized Programs of Research Excellence (SPORE) grant (P50-CA186786, to A.M.C.), an NCI Outstanding Investigator Award (R35-CA231996, to A.M.C.), and the Prostate Cancer Foundation. J.C.-Y.T. is supported by a Department of Defense Prostate Cancer Research Program Idea Development Award (W81XWH-21-1-0458). A.M.C. is a Howard Hughes Medical Institute Investigator, A. Alfred Taubman Scholar, and American Cancer Society Professor.

AUTHOR CONTRIBUTIONS

Y. Chang, X.W., J.Y., K.D., and A.M.C. designed and conceived the project. J.Y. designed and synthesized all CDK12/13 degraders with assistance from L.Z. Y. Chang and X.W. performed all *in vitro* and functional genomic experiments with assistance from C.W., L.X., R.H., and W.R.L. Y. Chang and J.C.-Y.T. performed all animal efficacy studies with help from Y. Cheng. G.C., Y. Zhang, B.M., H.C., J.N.V., and S.M.D. carried out all bioinformatic analyses. R. Mannan, S.M., and R. Mehra carried out all histopathological evaluations of drug toxicity as well as quantified all histology-based data and immunohistochemistry. Z.W. supervised the molecule synthesis. K.Z. and P.Z. expressed and purified CDK12/13 proteins. W.H. and Y. Zhou supervised protein expression and co-crystal structure modeling. F.S., R.W., and X.C. generated next-generation sequencing libraries and performed the sequencing. Y. Chang, X.W., K.D., and A.M.C. wrote the manuscript and organized the final figures. S.J.M. assisted with writing and editing of the manuscript. All authors read, commented, and participated in review and editing of the manuscript.

DECLARATION OF INTERESTS

A.M.C. is a co-founder and serves on the scientific advisory board of the following: Lynx Dx, Flamingo Therapeutics, Medsyn Pharma, Oncopia Therapeutics, and Esanik Therapeutics. A.M.C. serves as an advisor to Aurigene Oncology Limited, Proteovant, Tempus, RAPPTA, and Ascentage. K.D. serves as a scientific advisor of Kinoteck Therapeutics Co. Ltd, Shanghai, and has received financial support from Livzon Pharmaceutical Group, Zhuhai, China. The University of Michigan and the Shanghai Institute of Organic Chemistry have filed patents on the CDK12/13 degraders and inhibitors mentioned in this manuscript. A.M.C., K.D., X.W., J.Y., Y. Chang, and J.C.-Y.T. have been named as co-inventors on these patents.

STAR★METHODS

Detailed methods are provided in the online version of this paper and include the following:

- KEY RESOURCES TABLE
- EXPERIMENTAL MODEL AND STUDY PARTICIPANT DETAILS
  - Cell lines and antibodies
  - *In vivo* models
- METHOD DETAILS
  - Computational modeling
  - Cell viability assay
  - IncuCyte proliferation assays
  - Western blot
  - TMT mass spectrometry
  - RNA isolation and quantitative real-time PCR
  - RNA-seq and analysis
  - EU-RNA-seq
  - Comet assay
  - Cell cycle by flow cytometry
  - Assessment of drug synergism
  - Human phosphorylation pathway profiling array
  - Transfection
  - Immunohistochemistry
- QUANTIFICATION AND STATISTICAL ANALYSIS

SUPPLEMENTAL INFORMATION

Supplemental information can be found online at https://doi.org/10.1016/j.xcrm.2024.101752.

Received: April 8, 2024
Revised: June 29, 2024
Accepted: September 5, 2024
Published: September 30, 2024

REFERENCES

1. Siegel, R.L., Giaquinto, A.N., and Jemal, A. (2024). Cancer statistics, 2024. CA. Cancer J. Clin. *74*, 12–49. https://doi.org/10.3322/caac.21820.
2. Albertsen, P. (2009). Androgen deprivation in prostate cancer–step by step. N. Engl. J. Med. *360*, 2572–2574. https://doi.org/10.1056/NEJMe0901737.
3. Davis, I.D., Martin, A.J., Stockler, M.R., Begbie, S., Chi, K.N., Chowdhury, S., Coskinas, X., Frydenberg, M., Hague, W.E., Horvath, L.G., et al. (2019). Enzalutamide with standard first-line therapy in metastatic prostate cancer. N. Engl. J. Med. *381*, 121–131. https://doi.org/10.1056/NEJMoa1903835.
4. Tran, C., Ouk, S., Clegg, N.J., Chen, Y., Watson, P.A., Arora, V., Wongvipat, J., Smith-Jones, P.M., Yoo, D., Kwon, A., et al. (2009). Development of a second-generation antiandrogen for treatment of advanced prostate cancer. Science *324*, 787–790. https://doi.org/10.1126/science.1168175.
5. Zong, Y., and Goldstein, A.S. (2013). Adaptation or selection—mechanisms of castration-resistant prostate cancer. Nat. Rev. Urol. *10*, 90–98. https://doi.org/10.1038/nrurol.2012.237.
6. Chi, K., Hotte, S.J., Joshua, A.M., North, S., Wyatt, A.W., Collins, L.L., and Saad, F. (2015). Treatment of mCRPC in the AR-axis-targeted therapy-resistant state. Ann. Oncol. *26*, 2044–2056. https://doi.org/10.1093/annonc/mdv267.
7. Watson, P.A., Arora, V.K., and Sawyers, C.L. (2015). Emerging mechanisms of resistance to androgen receptor inhibitors in prostate cancer. Nat. Rev. Cancer *15*, 701–711. https://doi.org/10.1038/nrc4016.
8. Litwin, M.S., and Tan, H.-J. (2017). The diagnosis and treatment of prostate cancer: A review. JAMA *317*, 2532–2542. https://doi.org/10.1001/jama.2017.7248.
9. Fan, Z., Devlin, J.R., Hogg, S.J., Doyle, M.A., Harrison, P.F., Todorovski, I., Cluse, L.A., Knight, D.A., Sandow, J.J., Gregory, G., et al. (2020). CDK13 cooperates with CDK12 to control global RNA polymerase II processivity. Sci. Adv. *6*, eaaz5041. https://doi.org/10.1126/sciadv.aaz5041.
10. Krajewska, M., Dries, R., Grassetti, A.V., Dust, S., Gao, Y., Huang, H., Sharma, B., Day, D.S., Kwiatkowski, N., Pomaville, M., et al. (2019). CDK12 loss in cancer cells affects DNA damage response genes through premature cleavage and polyadenylation. Nat. Commun. *10*, 1757. https://doi.org/10.1038/s41467-019-09703-y.
11. Dubbury, S.J., Boutz, P.L., and Sharp, P.A. (2018). CDK12 regulates DNA repair genes by suppressing intronic polyadenylation. Nature *564*, 141–145. https://doi.org/10.1038/s41586-018-0758-y.
12. Chirackal Manavalan, A.P., Pilarova, K., Kluge, M., Bartholomeeusen, K., Rajecky, M., Oppelt, J., Khirsariya, P., Paruch, K., Krejci, L., Friedel, C.C., and Blazek, D. (2019). CDK12 controls G1/S progression by regulating RNAPII processivity at core DNA replication genes. EMBO Rep. *20*, e47592. https://doi.org/10.15252/embr.201847592.
13. Zhang, T., Kwiatkowski, N., Olson, C.M., Dixon-Clarke, S.E., Abraham, B.J., Greifenberg, A.K., Ficarro, S.B., Elkins, J.M., Liang, Y., Hannett, N.M., et al. (2016). Covalent targeting of remote cysteine residues to develop CDK12 and CDK13 inhibitors. Nat. Chem. Biol. *12*, 876–884. https://doi.org/10.1038/nchembio.2166.
14. Liang, K., Gao, X., Gilmore, J.M., Florens, L., Washburn, M.P., Smith, E., and Shilatifard, A. (2015). Characterization of human cyclin-dependent kinase 12 (CDK12) and CDK13 complexes in C-terminal domain

phosphorylation, gene transcription, and RNA processing. Mol. Cell Biol. *35*, 928–938. https://doi.org/10.1128/MCB.01426-14.

15. Blazek, D., Kohoutek, J., Bartholomeeusen, K., Johansen, E., Hulinkova, P., Luo, Z., Cimermancic, P., Ule, J., and Peterlin, B.M. (2011). The Cyclin K/Cdk12 complex maintains genomic stability via regulation of expression of DNA damage response genes. Genes Dev. *25*, 2158–2172. https://doi.org/10.1101/gad.16962311.
16. Quereda, V., Bayle, S., Vena, F., Frydman, S.M., Monastyrskyi, A., Roush, W.R., and Duckett, D.R. (2019). Therapeutic targeting of CDK12/CDK13 in triple-negative breast cancer. Cancer Cell *36*, 545–558.e7. https://doi.org/10.1016/j.ccell.2019.09.004.
17. Wu, Y.M., Cieślik, M., Lonigro, R.J., Vats, P., Reimers, M.A., Cao, X., Ning, Y., Wang, L., Kunju, L.P., de Sarkar, N., et al. (2018). Inactivation of CDK12 Delineates a Distinct Immunogenic Class of Advanced Prostate Cancer. Cell *173*, 1770–1782.e14. https://doi.org/10.1016/j.cell.2018.04.034.
18. Sokol, E.S., Pavlick, D., Frampton, G.M., Ross, J.S., Miller, V.A., Ali, S.M., Lotan, T.L., Pardoll, D.M., Chung, J.H., and Antonarakis, E.S. (2019). Pan-cancer analysis of CDK12 loss-of-function alterations and their association with the focal tandem-duplicator phenotype. Oncol. *24*, 1526–1533. https://doi.org/10.1634/theoncologist.2019-0214.
19. Marshall, C.H., Imada, E.L., Tang, Z., Marchionni, L., and Antonarakis, E.S. (2019). CDK12 inactivation across solid tumors: an actionable genetic subtype. Oncoscience *6*, 312–316. https://doi.org/10.18632/oncoscience.481.
20. Iniguez, A.B., Stolte, B., Wang, E.J., Conway, A.S., Alexe, G., Dharia, N.V., Kwiatkowski, N., Zhang, T., Abraham, B.J., Mora, J., et al. (2018). EWS/FLI confers tumor cell synthetic lethality to CDK12 inhibition in Ewing sarcoma. Cancer Cell *33*, 202–216.e6. https://doi.org/10.1016/j.ccell.2017.12.009.
21. Ekumi, K.M., Paculova, H., Lenasi, T., Pospichalova, V., Bösken, C.A., Rybarikova, J., Bryja, V., Geyer, M., Blazek, D., and Barboric, M. (2015). Ovarian carcinoma CDK12 mutations misregulate expression of DNA repair genes via deficient formation and function of the Cdk12/CycK complex. Nucleic Acids Res. *43*, 2575–2589. https://doi.org/10.1093/nar/gkv101.
22. Bajrami, I., Frankum, J.R., Konde, A., Miller, R.E., Rehman, F.L., Brough, R., Campbell, J., Sims, D., Rafiq, R., Hooper, S., et al. (2014). Genome-wide profiling of genetic synthetic lethality identifies CDK12 as a novel determinant of PARP1/2 inhibitor sensitivity. Cancer Res. *74*, 287–297. https://doi.org/10.1158/0008-5472.CAN-13-2541.
23. Manning, B.D., and Toker, A. (2017). AKT/PKB signaling: Navigating the network. Cell *169*, 381–405. https://doi.org/10.1016/j.cell.2017.04.001.
24. Davies, M.A. (2011). Regulation, role, and targeting of Akt in cancer. J. Clin. Oncol. *29*, 4715–4717. https://doi.org/10.1200/JCO.2011.37.4751.
25. Grasso, C.S., Wu, Y.-M., Robinson, D.R., Cao, X., Dhanasekaran, S.M., Khan, A.P., Quist, M.J., Jing, X., Lonigro, R.J., Brenner, J.C., et al. (2012). The mutational landscape of lethal castration-resistant prostate cancer. Nature *487*, 239–243. https://doi.org/10.1038/nature11125.
26. Robinson, D., Van Allen, E.M., Wu, Y.-M., Schultz, N., Lonigro, R.J., Mosquera, J.-M., Montgomery, B., Taplin, M.-E., Pritchard, C.C., Attard, G., et al. (2015). Integrative clinical genomics of advanced prostate cancer. Cell *161*, 1215–1228. https://doi.org/10.1016/j.cell.2015.05.001.
27. Carver, B.S., Chapinski, C., Wongvipat, J., Hieronymus, H., Chen, Y., Chandarlapaty, S., Arora, V.K., Le, C., Koutcher, J., Scher, H., et al. (2011). Reciprocal feedback regulation of PI3K and androgen receptor signaling in PTEN-deficient prostate cancer. Cancer Cell *19*, 575–586. https://doi.org/10.1016/j.ccr.2011.04.008.
28. Shariati, M., and Meric-Bernstam, F. (2019). Targeting AKT for cancer therapy. Expet Opin. Invest. Drugs *28*, 977–988. https://doi.org/10.1080/13543784.2019.1676726.
29. Mullard, A. (2024). FDA approves first-in-class AKT inhibitor. Nat. Rev. Drug Discov. *23*, 9. https://doi.org/10.1038/d41573-023-00202-w.
30. Tortorella, E., Giantulli, S., Sciarra, A., and Silvestri, I. (2023). AR and PI3K/AKT in prostate cancer: A tale of two interconnected pathways. Int. J. Mol. Sci. *24*, 2046. https://doi.org/10.3390/ijms24032046.
31. Sweeney, C., Bracarda, S., Sternberg, C.N., Chi, K.N., Olmos, D., Sandhu, S., Massard, C., Matsubara, N., Alekseev, B., Parnis, F., et al. (2021). Ipatasertib plus abiraterone and prednisolone in metastatic castration-resistant prostate cancer (IPATential150): a multicentre, randomised, double-blind, phase 3 trial. Lancet *398*, 131–142. https://doi.org/10.1016/S0140-6736(21)00580-8.
32. Houles, T., Lavoie, G., Nourreddine, S., Cheung, W., Vaillancourt-Jean, É., Guérin, C.M., Bouttier, M., Grondin, B., Lin, S., Saba-El-Leil, M.K., et al. (2022). CDK12 is hyperactivated and a synthetic-lethal target in BRAF-mutated melanoma. Nat. Commun. *13*, 6457. https://doi.org/10.1038/s41467-022-34179-8.
33. Wang, C., Wang, H., Lieftink, C., du Chatinier, A., Gao, D., Jin, G., Jin, H., Beijersbergen, R.L., Qin, W., and Bernards, R. (2020). CDK12 inhibition mediates DNA damage and is synergistic with sorafenib treatment in hepatocellular carcinoma. Gut *69*, 727–736. https://doi.org/10.1136/gutjnl-2019-318506.
34. Niu, T., Li, K., Jiang, L., Zhou, Z., Hong, J., Chen, X., Dong, X., He, Q., Cao, J., Yang, B., and Zhu, C.L. (2022). Noncovalent CDK12/13 dual inhibitors-based PROTACs degrade CDK12-Cyclin K complex and induce synthetic lethality with PARP inhibitor. Eur. J. Med. Chem. *228*, 114012. https://doi.org/10.1016/j.ejmech.2021.114012.
35. Tadesse, S., Duckett, D.R., and Monastyrskyi, A. (2021). The promise and current status of CDK12/13 inhibition for the treatment of cancer. Future Med. Chem. *13*, 117–141. https://doi.org/10.4155/fmc-2020-0240.
36. Mullard, A. (2021). Targeted protein degraders crowd into the clinic. Nat. Rev. Drug Discov. *20*, 247–250. https://doi.org/10.1038/d41573-021-00052-4.
37. Békés, M., Langley, D.R., and Crews, C.M. (2022). PROTAC targeted protein degraders: the past is prologue. Nat. Rev. Drug Discov. *21*, 181–200. https://doi.org/10.1038/s41573-021-00371-6.
38. Jiang, B., Gao, Y., Che, J., Lu, W., Kaltheuner, I.H., Dries, R., Kalocsay, M., Berberich, M.J., Jiang, J., You, I., et al. (2021). Discovery and resistance mechanism of a selective CDK12 degrader. Nat. Chem. Biol. *17*, 675–683. https://doi.org/10.1038/s41589-021-00765-y.
39. Yang, J., Chang, Y., Tien, J.C.-Y., Wang, Z., Zhou, Y., Zhang, P., Huang, W., Vo, J., Apel, I.J., Wang, C., et al. (2022). Discovery of a highly potent and selective dual PROTAC degrader of CDK12 and CDK13. J. Med. Chem. *65*, 11066–11083. https://doi.org/10.1021/acs.jmedchem.2c00384.
40. Palozola, K.C., Donahue, G., and Zaret, K.S. (2021). EU-RNA-seq for in vivo labeling and high throughput sequencing of nascent transcripts. STAR Protoc. *2*, 100651. https://doi.org/10.1016/j.xpro.2021.100651.
41. Ito, T., Yamaguchi, Y., and Handa, H. (2021). Exploiting ubiquitin ligase cereblon as a target for small-molecule compounds in medicine and chemical biology. Cell Chem. Biol. *28*, 987–999. https://doi.org/10.1016/j.chembiol.2021.04.012.
42. Zheng, H.-C. (2017). The molecular mechanisms of chemoresistance in cancers. Oncotarget *8*, 59950–59964. https://doi.org/10.18632/oncotarget.19048.
43. Liu, W., Zhang, Z., Zhang, Y., Chen, X., Guo, S., Lei, Y., Xu, Y., Ji, C., Bi, Z., and Wang, K. (2015). HMGB1-mediated autophagy modulates sensitivity of colorectal cancer cells to oxaliplatin via MEK/ERK signaling pathway. Cancer Biol. Ther. *16*, 511–517. https://doi.org/10.1080/15384047.2015.1017691.
44. Cheraghchi-Bashi, A., Parker, C.A., Curry, E., Salazar, J.-F., Gungor, H., Saleem, A., Cunnea, P., Rama, N., Salinas, C., Mills, G.B., et al. (2015). A putative biomarker signature for clinically effective AKT inhibition: correlation of in vitro, in vivo and clinical data identifies the importance of modulation of the mTORC1 pathway. Oncotarget *6*, 41736–41749. https://doi.org/10.18632/oncotarget.6153.

45. Coleman, N., Moyers, J.T., Harbery, A., Vivanco, I., and Yap, T.A. (2021). Clinical development of AKT inhibitors and associated predictive biomarkers to guide patient treatment in cancer medicine. Pharmgenomics. Pers. Med *14*, 1517–1535. https://doi.org/10.2147/PGPM.S305068.

46. Dumble, M., Crouthamel, M.-C., Zhang, S.-Y., Schaber, M., Levy, D., Robell, K., Liu, Q., Figueroa, D.J., Minthorn, E.A., Seefeld, M.A., et al. (2014). Discovery of novel AKT inhibitors with enhanced anti-tumor effects in combination with the MEK inhibitor. PLoS One *9*, e100880. https://doi.org/10.1371/journal.pone.0100880.

47. Tien, J.C.Y., Chang, Y., Zhang, Y., Chou, J., Cheng, Y., Wang, X., Yang, J., Mannan, R., Shah, P., Wang, X.M., et al. (2024). CDK12 Loss Promotes Prostate Cancer Development While Exposing Vulnerabilities to Paralog-Based Synthetic Lethality. Preprint at bioRxiv, 585990, 2024.03.20. https://doi.org/10.1101/2024.03.20.585990.

48. Altomare, D.A., and Testa, J.R. (2005). Perturbations of the AKT signaling pathway in human cancer. Oncogene *24*, 7455–7464. https://doi.org/10.1038/sj.onc.1209085.

49. Cheng, J.Q., Lindsley, C.W., Cheng, G.Z., Yang, H., and Nicosia, S.V. (2005). The Akt/PKB pathway: molecular target for cancer drug discovery. Oncogene *24*, 7482–7492. https://doi.org/10.1038/sj.onc.1209088.

50. Braglia, L., Zavatti, M., Vinceti, M., Martelli, A.M., and Marmiroli, S. (2020). Deregulated PTEN/PI3K/AKT/mTOR signaling in prostate cancer: Still a potential druggable target? Biochim. Biophys. Acta Mol. Cell Res. *1867*, 118731. https://doi.org/10.1016/j.bbamcr.2020.118731.

51. Toren, P., and Zoubeidi, A. (2014). Targeting the PI3K/Akt pathway in prostate cancer: challenges and opportunities (review). Int. J. Oncol. *45*, 1793–1801. https://doi.org/10.3892/ijo.2014.2601.

52. Dong, C., Wu, J., Chen, Y., Nie, J., and Chen, C. (2021). Activation of PI3K/AKT/mTOR pathway causes drug resistance in breast cancer. Front. Pharmacol. *12*, 628690. https://doi.org/10.3389/fphar.2021.628690.

53. Surucu, B., Bozulic, L., Hynx, D., Parcellier, A., and Hemmings, B.A. (2008). In vivo analysis of protein kinase B (PKB)/Akt regulation in DNA-PKcs-null mice reveals a role for PKB/Akt in DNA damage response and tumorigenesis. J. Biol. Chem. *283*, 30025–30033. https://doi.org/10.1074/jbc.M803053200.

54. Xiao, L., Parolia, A., Qiao, Y., Bawa, P., Eyunni, S., Mannan, R., Carson, S.E., Chang, Y., Wang, X., Zhang, Y., et al. (2022). Targeting SWI/SNF ATPases in enhancer-addicted prostate cancer. Nature *601*, 434–439. https://doi.org/10.1038/s41586-021-04246-z.

STAR★METHODS

KEY RESOURCES TABLE

REAGENT or RESOURCE	SOURCE	IDENTIFIER
Antibodies		
CDK12	Proteintech	Cat# 26816-1-AP; RRID:AB_2880645
CDK13	Millipore	Cat# ABE1860
RNA pol II CTD phospho Ser2	Cell Signaling Technology	Cat# 13499S; RRID: RRID: AB_2798238
c-PARP	Cell Signaling Technology	Cat# 5625S; RRID:AB_10699459
γH2AX	Abcam	Cat# ab11174; RRID:AB_297813
CCNK	Bethyl Laboratories	Cat# A301-939A; RRID:AB_1547934
p-AKT (ser473)	Cell Signaling Technology	Cat# 4060S; RRID:AB_2315049
p-PRAS40	Cell Signaling Technology	Cat# 2997S; RRID:AB_2258110
AKT	Cell Signaling Technology	Cat# 4691S; RRID:AB_915783
PRAS40	Cell Signaling Technology	Cat# 2610S; RRID:AB_916206
p-S6 (Ser235/236)	Cell Signaling Technology	Cat# 2211S; RRID:AB_331679
GSPT1	Proteintech	Cat# 10763-1-AP; RRID:AB_2115506
IKZF1	Cell Signaling Technology	Cat# 14859S; RRID:AB_2744523
IKZF3	Cell Signaling Technology	Cat# 15103S; RRID:AB_2744524
CDK12 (IHC)	Sigma-Aldrich	Cat# HPA008038-25; RRID:AB_1078570
p-AKT (T308)	Cell Signaling Technology	Cat# 9275S; RRID:AB_329828
phoGSK2β (s9)	Cell Signaling Technology	Cat# 9336S; RRID:AB_331405
GSK2β	Proteintech	Cat# 22104-1-AP; RRID:AB_2878997
FYTTD1	Invitrogen	Cat# PA5-98705; RRID:AB_2813318
DDIT3	Proteintech	Cat# 15204-1-AP; RRID:AB_2292610
MAPK9	Proteintech	Cat# 51153-1-AP; RRID:AB_10898168
CDK9	Cell Signaling Technology	Cat# 2316S; RRID:AB_2291505
Vinculin	Proteintech	Cat# 66305-1-Ig; RRID:AB_2810300
GAPDH	Cell Signaling Technology	Cat# 3683; RRID:AB_1642205
Tubulin	Abcam	Cat# ab184577; RRID:AB_3661661
Biological samples		
WA74 Patient-derived xenografts (PDX)	University of Michigan	N/A
PC310 Patient-derived xenografts (PDX)	Erasmus Medical Center, Rotterdam, the Netherlands	N/A
Chemicals, peptides, and recombinant proteins		
Uprosertib	TargetMol	Cat# T6849
MK2206	Selleck Chemicals	Cat# S1078
Capivasertib	Selleck Chemicals	Cat# S8019
Thalidomide	Selleck Chemicals	Cat# S1193
Carfilzomib	Selleck Chemicals	Cat# S2853
Bafilomycin	Selleck Chemicals	Cat# S1413
YJ9069	This paper	N/A
YJ1206	This paper	N/A
YJ9068	This paper	N/A
YJ1078	This paper	N/A
YJ1090	This paper	N/A
YJ1094	This paper	N/A
YJ1114	This paper	N/A

(*Continued on next page*)

Continued

REAGENT or RESOURCE	SOURCE	IDENTIFIER
YJ1130	This paper	N/A
YJ1096	This paper	N/A
YJ1105	This paper	N/A
YJ1205	This paper	N/A
Lipofectamine™ RNAiMAX Transfection Reagent	Thermo Scientific	Cat# 13778075
Discovery CC1	Roche-Ventana Medical System	Cat# 06414575001
Discovery Inhibitor	Roche-Ventana Medical System	Cat# 760-4840
Discovery OmniMap anti-rabbit HRP	Roche-Ventana Medical System	Cat# 760-4311
Discovery OmniMap anti-mouse HRP	Roche-Ventana Medical System	Cat# 760-4310
**Critical commercial assays**		
CellTiter-Glo® Luminescent Cell Viability Assay	Promega	Cat# G7572
TMTpro™ 16plex Label Reagent Set	Thermo Scientific	Cat# A44521
RNeasy Kits for RNA Purification	Qiagen	Cat# 74104
SuperScript™ III One-Step RT-PCR System with Platinum™ Taq DNA Polymerase	Thermo Scientific	Cat# 12574026
Fast SYBR™ Green Master Mix	Thermo Scientific	Cat# 4385612
Click-iT™ Nascent RNA Capture Kit, for gene expression analysis	Thermo Scientific	Cat# C10365
OxiSelect™ Comet Assay Kit	Cell Biolabs	Cat# STA-351
Human Phosphorylation Pathway Profiling Array C55	Ray Biotech	AAH-PPP-1-4/501948639
**Deposited data**		
Raw and analyzed data	This paper	N/A
RNA-seq data in VCaP	This paper	GEO: GSE262119
EU-RNA-seq data in VCaP	This paper	GEO: GSE262120
**Experimental models: Cell lines**		
VCaP	ATCC	RRID: CVCL_2235
22Rv1	ATCC	RRID: CVCL_1045
RWPE-1	ATCC	RRID: CVCL_3791
BPH-1	Sigma-Aldrich	RRID: CVCL_1091
LAPC4	ATCC	RRID: CVCL_4744
PC3	ATCC	RRID: CVCL_0035
DU145	ATCC	RRID: CVCL_0105
WPMY-1	ATCC	RRID: CVCL_3814
PrEC	ATCC	PCS-440-010
PNT2	Sigma-Aldrich	RRID: CVCL_2164
MFM-223	DSMZ	RRID: CVCL_1408
MDA-MB-468	ATCC	RRID: CVCL_0419
SK-BR-3	ATCC	RRID: CVCL_0033
MCF7	ATCC	RRID: CVCL_0031
MCF10A	ATCC	RRID: CVCL_0598
MCF12A	ATCC	RRID: CVCL_3744
**Experimental models: Organisms/strains**		
Mouse: CB17SCID	Charles River Laboratories	Stock number 236
**Oligonucleotides**		
*ATM*_Fwd: GCTGACAATCATCACCAAGT	Quereda, V. et al.[16]	N/A
*ATM*_Rev: GGTTCTCAGCACTATGGGACA	Quereda, V. et al.[16]	N/A
*ATR*_Fwd: CGCTGAACTGTACGTGGAAA	Quereda, V. et al.[16]	N/A

(*Continued on next page*)

Continued		
REAGENT or RESOURCE	SOURCE	IDENTIFIER
*ATR*_Rev: CAATTAGTGCCTGGTGAACATC	Quereda, V. et al.[16]	N/A
*BRCA1*_Fwd: CTGCTCAGGGCTATCCTCTCA	Quereda, V. et al.[16]	N/A
*BRCA1*_Rev: GCTTCTAGTTCAGCCATTTCCTG	Quereda, V. et al.[16]	N/A
*CDK12*_Fwd: CCAATCTGGAACTGGCTCAG	Quereda, V. et al.[16]	N/A
*CDK12*_Rev: CAAGTGCTGCAGAAGGAATG	Quereda, V. et al.[16]	N/A
*CDK13*_Fwd: GGTGTTTGAATATATGGACC	Quereda, V. et al.[16]	N/A
*CDK13*_Rev: CAAGTCCAAAGTCTGCAAGTT	Quereda, V. et al.[16]	N/A
Software and algorithms		
PRISM	GraphPad Software	Version 10
ImageJ	NIH	https://imagej.nih.gov/ij/
ImageStudio Lite	Li-Cor	Ver5.2
Pymol	Schrödinger	https://www.schrodinger.com/products/pymol
Maestro	Schrödinger	Version 9.9
FlowJo	FlowJo Software	Version 10.8.2
SynergyFinder	Oxford Academic	https://synergyfinder.fimm.fi/synergy

EXPERIMENTAL MODEL AND STUDY PARTICIPANT DETAILS

Cell lines and antibodies

Cell lines in this paper were acquired from ATCC or our internal stock. All cell lines were tested with genotyping verification at the University of Michigan Sequencing Core and were routinely tested for Mycoplasma contamination. VCaP cells were cultured in DMEM+GlutaMAX (Gibco) with 10% fetal bovine serum (FBS, ThermoFisher Scientific). 22Rv1 and BPH-1 cells were maintained in RPMI-1640 (Gibco) with 10% FBS. RWPE cells were cultured in Keratinocyte medium (Gibco) with bovine pituitary extract (BPE) and human recombinant epidermal growth factor (EGF). Detailed antibody information is listed in the key resources table.

In vivo models

Six-week-old male CB17SCID mice obtained from Charles River (Stock # 236), were used to establish subcutaneous tumors on both dorsal flanks. Tumor dimensions were measured biweekly using digital calipers, applying the formula $(\pi/6)\ (L \times W^2)$, where L and W represent tumor length and width, respectively. At the study conclusion, mice were euthanized, and tumors were excised and weighed. The University of Michigan Institutional Animal Care and Use Committee (IACUC) approved all *in vivo* studies.

For the VCaP castration-resistant tumor model, 3×10^6 VCaP cells were subcutaneously injected into both dorsal flanks of mice using a serum-free medium mixed with 50% Matrigel (BD Biosciences). Upon tumors reaching palpable size (~200 mm^3), animals underwent castration. When tumors regrew to their pre-castration size, mice were randomized for treatment with 30 mg/kg YJ9069 or vehicle via intravenous injection or 100 mg/kg YJ1206 or vehicle, with or without 15 mg/kg uprosertib by oral gavage. Treatments were administered three times weekly for three to four weeks.

For the 22Rv1 castration-resistant tumor model, mice underwent castration and then 12 days of recovery. Subsequently, 1×10^6 22Rv1 cells were subcutaneously injected into both dorsal flanks using a serum-free medium with 50% Matrigel (BD Biosciences). Once tumors reached a palpable size (~200 mm^3), mice were randomized to receive either 100 mg/kg YJ1206 or vehicle orally 3 times weekly, with or without 15 mg/kg uprosertib orally five times weekly, for a duration of four weeks.

WA74 PDX model was developed at the University of Michigan during collection of a rapid autopsy case as part of the Michigan Legacy Tissue Program (MLTP). PC310 PDX model was obtained from Erasmus Medical Center, Rotterdam, the Netherlands. Both PDX lines were propagated in male CB17SCID mice by surgically implanting 2 mm^3 tumor fragments, coated with 100% Matrigel, into both flanks of mice. When tumors reached approximately 200 mm^3, mice were randomized into treatment groups. These groups received either 30 mg/kg YJ9069 via intravenous injection or 100 mg/kg YJ1206 by oral gavage, both administered three times per week, with or without 15 mg/kg uprosertib by oral gavage five days per week for 3–4 weeks. In accordance with IACUC guidelines, the maximum tumor size was limited to 2.0 cm in any dimension, and mice were euthanized if xenografts reached this endpoint.

METHOD DETAILS

Computational modeling

Molecular modeling procedures were conducted using Maestro (version 9.9, Schrödinger, LLC, New York, NY, 2014) within the Schrödinger suite (öhttp://www.schrödinger.com). Crystal structures of CDK12 and CDK13 with the inhibitors (PDB codes: 7NXK and 7NXJ, respectively) were sourced from the Protein DataBank (http://www.pdb.org). The Protein Preparation Wizard in Maestro

was used to prepare the proteins, which included adding bond orders and hydrogens and removing water molecules. Degrader structures were prepared using LigPrep (version 3.1, Schrödinger, LLC) with the OPLS-2005 force field. The docking grid was centered on the ligand binding site with a bounding box of 18 Å, and Glide docked flexible ligands into a rigid receptor structure. Molecular docking was carried out using the Glide program (version 6.4, Schrödinger, LLC) in Standard Precision (SP) mode. Visualization of the results was done using PyMol (https://www.schrodinger.com/products/pymol).

Cell viability assay

Cells were seeded in 96-well plates and incubated at 37°C with 5% CO_2 overnight. A series of compound dilutions were added into the cells. After 5 days incubation, cell proliferation was tested using the CellTiter-Glo assay (Promega) according to the manufacturer's protocol. Luminescence was measured using the Infinite M1000 Pro plate reader (Tecan). Data analysis was conducted using GraphPad Prism software.

IncuCyte proliferation assays

Cell proliferation was quantitatively assessed using the IncuCyte Live-Cell Analysis System (Essen Bioscience). VCaP, 22Rv1, RWPE, and BPH-1 cells were plated in 96-well plates. Following overnight incubation at 37°C and 5% CO_2, the cells were treated with varying concentrations of YJ1206, YJ9069, or siRNA, with or without AKT inhibitors. Real-time cell proliferation was monitored by capturing phase-contrast images every 4 h using a 10× objective. The IncuCyte software (version 2022A Rev1) was utilized to measure cell confluence continuously as a proxy for proliferation. Data analysis was performed using the software's built-in analytical tools, focusing on growth curves and confluence metrics (percentage area). Figures were generated using GraphPad Prism software.

Western blot

After treatment of varying conditions, cell lysates were prepared using RIPA buffer (ThermoFisher Scientific) supplemented with cOmplete protease inhibitor cocktail tablets (Sigma-Aldrich). The Pierce BCA Protein Assay Kit (Bio-Rad) was used to determine the protein concentrations. Proteins with an equal amount from each sample were loaded on NuPAGE 3–8% Tris-Acetate or 4–12% Bis-Tris protein gels (ThermoFisher Scientific) and subsequently transferred to membranes, which were blocked using 5% nonfat milk. Following blocking at room temperature for 1 h, the membrane was further incubated with varying primary antibodies at 4°C overnight. HRP-conjugated secondary antibodies were used for detection, and membrane imaging was conducted using an Odyssey Fc Imager (LI-COR Biosciences).

TMT mass spectrometry

VCaP or 22Rv1 cells were seeded at a density of 1×10^7 or 5×10^6 cells on 100 mm plates 24 h before treatment. Cells were treated in triplicate with YJ9069 or YJ1206. After 6 h incubation, cells were harvested and lysed using RIPA buffer (Thermo Fisher Scientific). The Pierce BCA Protein Assay Kit (Bio-Rad) was used to determine the protein concentration. Lysates were then proteolyzed and labeled with TMT 10-plex Isobaric Label Reagent (Thermo Fisher Scientific, 90110), following the manufacturer's protocol. This involved reduction, alkylation, precipitation in cold acetone, and overnight incubation at −20°C. The pellet was air-dried, resuspended in 0.1M TEAB, and digested with trypsin at a 1:50 enzyme-to-protein ratio at 37°C overnight. The TMT 10-plex reagents, dissolved in anhydrous acetonitrile, were added to the digests for labeling, followed by quenching with hydroxylamine. The labeled samples were combined, dried, and fractionated into 10 parts using a high pH reversed-phase peptide fractionation kit (Pierce, 84868).

For LC-MS/MS analysis, fractions were reconstituted in 0.1% formic acid/2% acetonitrile. Using multinotch-MS3 on an Orbitrap Fusion (ThermoFisher Scientific) coupled with an RSLC Ultimate 3000 nano-UPLC (Dionex), samples (2 mL) were resolved on a PepMap RSLC C18 column (75 μm i.d. × 50 cm; Thermo Scientific) setting the flow rate of 300 nL/min using a 0.1% formic acid/acetonitrile gradient system (2–22% acetonitrile for 150 min; 22–32% acetonitrile for 40 min; washing for 20 min at 90% followed by re-equilibration for 50 min) and direct spray into the mass spectrometer using EasySpray source (ThermoFisher Scientific). The mass spectrometer captured one MS1 scan at a resolution of 60000 with a AGC target of 2×10^5 (Orbitrap; MAX injection 100 ms). The following data-dependent Top Speed (3 s) MS2 scans with a AGC target of 5×10^3 (collision-induced dissociation; ion trap; NCD 35; MAX injection 100 ms) and multinotch-MS3 for the top 10 precursors. Proteome Discoverer (ThermoFisher, version 2.1) was used for data analysis against the SwissProt human protein database (release 11 November 2015; 42,084 sequences), with defined MS tolerances, modifications, and FDR threshold for filtering proteins and peptides. Quantitation relied on high-quality MS3 spectra (Average signal-to-noise ratio of 20 and <30% isolation interference).

RNA isolation and quantitative real-time PCR

Total RNA was extracted from VCaP or 22Rv1 cells using the RNeasy Mini Kit (Qiagen, 74104) following the manufacturer's protocol. The RNA concentration was determined by NanoDrop. 1000 ng total RNA was used for cDNA synthesis with the Maxima First Strand cDNA Synthesis Kit for RT-PCR (ThermoFisher Scientific, 12574026). Quantitative real-time PCR (qPCR) was conducted in triplicate using SYBR green reagents on a QuantStudio 6 Real-Time PCR system (Applied Biosystems). mRNA expression levels were quantified by the ΔCt method and normalized to *ACTB* expression. Primers were designed with Primer 3 (http://frodo.wi.mit.edu/primer3/) and obtained from Integrated DNA Technologies, with sequences detailed in the key resources table and Table S1.

RNA-seq and analysis

VCaP cells were treated with YJ9069 at the concentration of 500 nM. Following 5 h incubation, total RNA was extracted using RNeasy Mini Kit (Qiagen) and quantified by NanoDrop 2000 Spectrophotometers (Thermo Scientific). RNA-seq libraries were prepared using KAPA RNA Hyper+RiboErase HMR (Roche Cat#: 08098140702). Ribosomal RNA was removed by enzymatic digestion from 800 ng total RNA, following with cDNA and double strand cDNA synthesis, end repair, A-tailing, and ligation with NEB adapters. cDNA fragments with sizes between 250 and 300 bp were separated using double AMPure beads and PCR amplified using 2x KAPA HiFi HotStart mix and NEB dual indexes (Roche Cat#: E6440L). Library quality was measured on an Agilent 2100 Bioanalyzer for concentration and product size. Paired-end libraries were sequenced with the Illumina NovaSeq 6000 (2 × 150 nucleotide read length) with sequence coverage to 20–30 million paired reads. Data analysis was conducted as previously reported.[54]

EU-RNA-seq

VCaP cells were seeded at a density of 2×10^7 cells per 10 cm dish 24 h prior to treatment. The cells were treated in triplicate with YJ9069 at a concentration of 500 nM. 5-ethynyl Uridine (EU, Thermo Fisher Scientific, C10365) was added to a final concentration of 0.5 mM. After incubation for an additional 1 h, the cells were harvested, and total RNA was extracted using the RNeasy Mini Kit (Qiagen) and then quantified using a Qubit fluorometer. For each sample, 5 μg of the total RNA was diluted and incubated in a Click-iT reaction cocktail for 30 min with gentle vortexing. Chilled 100% ethanol was added, and the samples were incubated at −80°C overnight. Following this, the tubes were centrifuged, and the RNA pellets were dried at room temperature and then resuspended in 50 μL of RNase-free distilled water. For each sample, 1 μg of total biotinylated RNA was isolated, and three different spike-in controls (100 pg each) were added. The prepared RNA binding reaction mix was then introduced to the samples. This was followed by co-heating at 68°C for 5 min. The bead suspension was added, and the samples were incubated at room temperature for 30 min with gentle vortexing. After washing, RNA captured on the beads was processed for cDNA synthesis using the KAPA RNA Hyper Kit (Roche Cat#: 08098107702). The remaining steps were the same as those described above under the "RNA-seq and analysis" section.

Comet assay

VCaP cells were seeded in 6-well plates one day prior to treatment. YJ9069 was added into the medium at a final concentration of 200 nM. After 12 h incubation, the cells were trypsinized and resuspended at a concentration of 1×10^5 cells/mL in ice-cold PBS. The OxiSelect Comet Assay Kit (Cell Biolabs, STA-351) was used to assess DNA damage. Following the manufacturer's protocol, cells were combined with pre-heated comet agarose at a 1:10 ratio, mixed thoroughly, and then transferred onto slides. The slides were incubated horizontally in the dark at 4°C for 20 min. Afterward, they were immersed in pre-chilled lysis buffer for 40 min at 4°C in the dark, followed by a 30-min incubation in pre-chilled alkaline solution at 4°C in the dark. Electrophoresis was then performed using alkaline solution at 30 V for 20 min. After electrophoresis, the slides were rinsed three times with pre-chilled deionized water, following with 70% ethanol for 5 min and completely dried at 37°C for 30 min. Subsequently, 100 μL of diluted Vista Green DNA dye was applied to each well. After 15 min incubation at room temperature, the comets were observed using a fluorescence microscope and quantified using ImageJ. The quantification figure was generated using GraphPad Prism software.

Cell cycle by flow cytometry

VCaP cells were seeded in 6-well plates one day before treatment. Cells were treated with YJ9069 at a concentration of 100 nM or 500 nM. After treatment, cells were harvested and fixed with 70% ethanol at −20°C overnight. Cells were washed with PBS and stained with a Propidium Iodide solution at 50 μg/mL (PI, ThermoFisher Scientific, P3566) and RNase A at 100 μg/mL for 30 min in the dark at room temperature. Stained cells were then analyzed using SH800S cell sorter (Sony Biotechnology), with data collected on more than 10,000 cells to ensure a comprehensive profile. Using FlowJo software (version 10.8.2), the cell cycle phases (G0/G1, S, and G2/M) were distinguished and quantified by analyzing the PI fluorescence.

Assessment of drug synergism

To assess the synergy between the two drug treatments, cells were exposed to escalating concentrations of each drug for 4 days. Cell viability was determined post-treatment using the CellTiter-Glo Luminescent Cell Viability Assay (Promega) with three biological replicates. Results were calculated as percentage inhibition relative to control. The assessment of synergy was conducted using the Bliss method in SynergyFinder (Version 3.0, https://synergyfinder.fimm.fi/synergy).

Human phosphorylation pathway profiling array

VCaP and 22Rv1 cells were seeded in 10 cm dishes and incubated at 37°C with 5% CO_2 overnight. Cells were treated with YJ1206 at a concentration of 500 nM for 15 h. Human phosphorylation pathway profiling was conducted using a kit from Ray Biotech (AAH-PPP-1-8) according to the manufacturer's protocol. After drug treatment, cells were collected and lysed using lysis buffer containing protease and phosphatase inhibitor cocktails. Protein concentration was measured, and 1 mL of 1 mg/mL total protein from each sample was diluted to 5 mL. 1 mL of the diluted protein was added to each blocked membrane (total 5 different membrane relative to 5 pathways) and incubated at 4°C overnight. Subsequently, the membranes were washed and incubated with HRP-conjugated anti-rabbit IgG for 2 h at room temperature. This was followed by further washing, after which the membranes were incubated with a detection buffer mixture for 2 min at room temperature and immediately imaged using the Odyssey

Fc Imager (LI-COR Biosciences). Images were further quantified using ImageJ software. The bar graph was generated using GraphPad Prism software.

Transfection

VCaP or 22Rv1 cells were seeded in 6-well plates and incubated at 37°C with 5% CO_2 overnight. 3.5 µL RNAiMAX (Thermo Fisher Scientific, 13778075) was diluted in 125 µL of Opti-MEM (Thermo Fisher Scientific, 31985062). This solution was then combined with 125 µL of OptiMEM containing 100 nM siCDK12/13 (siCDK12, Horizon Discovery, J-004031-10-0050; siCDK13, Horizon Discovery, J-004688-06-0050). The mixtures were gently homogenized using a pipette and allowed to incubate at room temperature for 5 min. Subsequently, 250 µL mixture was added directly to the cells. Following an incubation of 15 h to permit gene silencing, the transfection medium was replaced with fresh culture medium.

Immunohistochemistry

Immunohistochemistry (IHC) assays were carried out on 4-micron formalin-fixed, paraffin-embedded (FFPE) tissue sections using the Ventana ULTRA automated slide stainer platform. Heat-induced epitope retrieval was performed using cell conditioning media 1, followed by primary antibody incubation for either 16 or 32 min at 37°C. The following antibodies were utilized: CDK12 (Sigma-Aldrich: HPA008038-25, rabbit polyclonal) and cPARP (Cell Signaling Technology: 5625s, rabbit monoclonal). Anti-rabbit or anti-mouse secondary antibodies were employed where applicable to develop the immune complexes. The OmniMap Universal DAB RTU detection kit was utilized to develop the complexes. The key resources table contains data on the essential reagents.

QUANTIFICATION AND STATISTICAL ANALYSIS

Experimental quantifications were conducted using the software specified in the respective methods sections. Statistical analyses predominantly employed the two-tailed, unpaired t-test, unless otherwise noted in the figure legends. p values are indicated as follows: ns (not significant), $*p < 0.05$, $**p < 0.01$, $***p < 0.001$, $****p < 0.0001$. Unless otherwise specified, samples were independent biological replicates.

Cell Reports Medicine, Volume *5*

Supplemental information

Development of an orally bioavailable CDK12/13 degrader and induction of synthetic lethality with AKT pathway inhibition

Yu Chang, Xiaoju Wang, Jianzhang Yang, Jean Ching-Yi Tien, Rahul Mannan, Gabriel Cruz, Yuping Zhang, Josh N. Vo, Brian Magnuson, Somnath Mahapatra, Hanbyul Cho, Saravana Mohan Dhanasekaran, Cynthia Wang, Zhen Wang, Licheng Zhou, Kaijie Zhou, Yang Zhou, Pujuan Zhang, Weixue Huang, Lanbo Xiao, Weihuang Raymond Liu, Rudana Hamadeh, Fengyun Su, Rui Wang, Stephanie J. Miner, Xuhong Cao, Yunhui Cheng, Rohit Mehra, Ke Ding, and Arul M. Chinnaiyan

Supplemental information

Development of an orally bioavailable CDK12/13 degrader and induction of synthetic lethality with AKT pathway inhibition

Yu Chang[1,2,8], Xiaoju Wang[1,2,3,8], Jianzhang Yang[4,5,8], Jean Ching-Yi Tien[1,2], Rahul Mannan[1,2], Gabriel Cruz[1,2], Yuping Zhang[1,2], Josh N. Vo[1,2], Brian Magnuson[1,2], Somnath Mahapatra[1,2], Hanbyul Cho[1,2], Saravana Mohan Dhanasekaran[1,2,3], Cynthia Wang[1,2], Zhen Wang[4], Licheng Zhou[4,5], Kaijie Zhou[4], Yang Zhou[5], Pujuan Zhang[4], Weixue Huang[4], Lanbo Xiao[1,2], Weihuang Raymond Liu[1], Rudana Hamadeh[1], Fengyun Su[1,2], Rui Wang[1,2], Stephanie J. Miner[1,2], Xuhong Cao[1,2,3,6], Yunhui Cheng[1,2], Rohit Mehra[1,2,3,7], Ke Ding[4,*], and Arul M. Chinnaiyan[1,2,3,6,7,9,*]

[1] Michigan Center for Translational Pathology, University of Michigan, Ann Arbor, MI, 48109, USA

[2] Department of Pathology, University of Michigan, Ann Arbor, MI, 48109, USA

[3] Rogel Cancer Center, University of Michigan, Ann Arbor, MI, 48109, USA

[4] State Key Laboratory of Chemical Biology, Shanghai Institute of Organic Chemistry, Chinese Academy of Sciences, Shanghai 200032, People's Republic of China

[5] School of Pharmaceutical Sciences, Jinan University, Guangzhou 511436, People's Republic of China

[6] Howard Hughes Medical Institute, University of Michigan, Ann Arbor, MI, 48109, USA

[7] Department of Urology, University of Michigan, Ann Arbor, MI, 48109, USA

[8]These authors contributed equally

[9]Lead contact

[*]Correspondence: arul@umich.edu (A.M.C.) and dingk@sioc.ac.cn (K.D.)

Figure S1

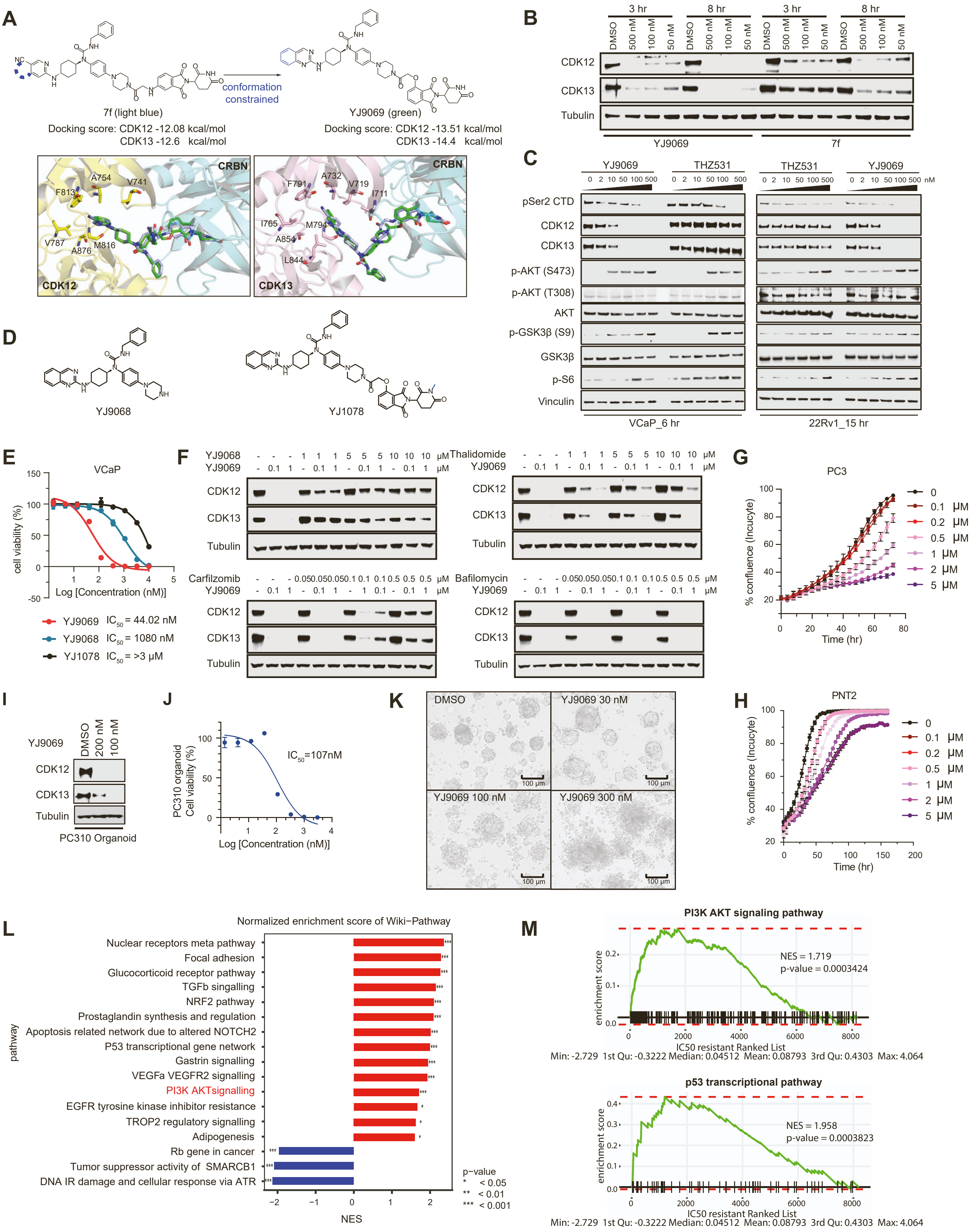
A
7f (light blue)
Docking score: CDK12 -12.08 kcal/mol
CDK13 -12.6 kcal/mol
conformation constrained
YJ9069 (green)
Docking score: CDK12 -13.51 kcal/mol
CDK13 -14.4 kcal/mol
CRBN
CDK12
CDK13
B
3 hr
8 hr
DMSO
500 nM
100 nM
50 nM
CDK12
CDK13
Tubulin
YJ9069
7f
C
YJ9069
THZ531
0 2 10 50 100 500 nM
pSer2 CTD
CDK12
CDK13
p-AKT (S473)
p-AKT (T308)
AKT
p-GSK3β (S9)
GSK3β
p-S6
Vinculin
VCaP_6 hr
22Rv1_15 hr
D
YJ9068
YJ1078
E
VCaP
cell viability (%)
Log [Concentration (nM)]
YJ9069 IC50 = 44.02 nM
YJ9068 IC50 = 1080 nM
YJ1078 IC50 = >3 μM
F
YJ9068
YJ9069
Thalidomide
Carfilzomib
Bafilomycin
CDK12
CDK13
Tubulin
G
PC3
% confluence (Incucyte)
Time (hr)
0
0.1 μM
0.2 μM
0.5 μM
1 μM
2 μM
5 μM
H
PNT2
I
YJ9069
DMSO
200 nM
100 nM
CDK12
CDK13
Tubulin
PC310 Organoid
J
PC310 organoid Cell viability (%)
IC50=107nM
Log [Concentration (nM)]
K
DMSO
YJ9069 30 nM
YJ9069 100 nM
YJ9069 300 nM
100 μm
L
Normalized enrichment score of Wiki−Pathway
Nuclear receptors meta pathway
Focal adhesion
Glucocorticoid receptor pathway
TGFb singalling
NRF2 pathway
Prostaglandin synthesis and regulation
Apoptosis related network due to altered NOTCH2
P53 transcriptional gene network
Gastrin signalling
VEGFa VEGFR2 signalling
PI3K AKTsignalling
EGFR tyrosine kinase inhibitor resistance
TROP2 regulatory signalling
Adipogenesis
Rb gene in cancer
Tumor suppressor activity of SMARCB1
DNA IR damage and cellular response via ATR
pathway
NES
p−value
* < 0.05
** < 0.01
*** < 0.001
M
PI3K AKT signaling pathway
enrichment score
NES = 1.719
p-value = 0.0003424
IC50 resistant Ranked List
Min: -2.729 1st Qu: -0.3222 Median: 0.04512 Mean: 0.08793 3rd Qu: 0.4303 Max: 4.064
p53 transcriptional pathway
NES = 1.958
p-value = 0.0003823

Figure S1. Design and characterization of YJ9069 in multiple cancer cell lines. Related to Figure 1.
(A) Design and modeling of a CDK12/13 degrader YJ9069 derived from 7f (*J Med Chem. 2022, 11066-11083*).
(B) Immunoblots of CDK12 and CDK13 in VCaP cells upon treatment with YJ9069 or 7f for 3 and 8 hours at the indicated concentrations. Tubulin is used as a loading control.
(C) Immunoblots of CDK12 and CDK13 in VCaP and 22Rv1 cells upon treatment with YJ9069 or THZ531 at increasing concentrations. Vinculin is used as a loading control.
(D) Chemical structures of YJ9068 (warhead of YJ9069) and YJ1078 (inactive degrader of YJ9069).
(E) Dose-response curves and IC_{50} of VCaP cells treated with YJ9069, YJ9068, and YJ1078 for 5 days. Data are presented as mean +/− SD from n=3 independent experiments.
(F) Immunoblots of CDK12 and CDK13 in VCaP cells pre-treated with YJ9068, thalidomide, carfilzomib, or bafilomycin for 1 hour, then treated with YJ9069 at noted concentrations for 4 hours. Tubulin is the loading control probed on all immunoblots.
(G-H) Growth curves of PC3 and PNT2 cells upon treatment with increasing concentrations of YJ9069. Data are presented as mean +/− SD from n=3 independent experiments.
(I) Immunoblots of CDK12 and CDK13 levels upon treatment of PC310 organoids with YJ9069 at 100 nM and 200 nM for 15 hours. Tubulin is used as a loading control.
(J) Dose-response curves and IC_{50} of PC310 organoids treated with YJ9069. Data are presented as mean +/− SD from n=3 independent experiments.
(K) Images of PC310 organoids after treatment with vehicle or YJ9069 at 30, 100, and 300 nM for 5 days (scale=100 μm).
(L-M) Gene Set Enrichment Analysis (GSEA) of cell viability in 124 cell lines (Fig. 1G). NES, normalized enrichment score. RNA-sequencing data from 124 cell lines were obtained from the Cancer Cell Line Encyclopedia (CCLE). Cell lines were categorized based on IC_{50} values from Figure 1G, with 30 annotated as "Sensitive" (< 500 nM) and the remaining 94 as "Resistant" (≥ 500 nM). Gene rankings were based on log fold change (logFC), and enrichment analysis was performed against curated pathways from the Molecular Signatures Database (MSigDB 3.0).

Figure S2

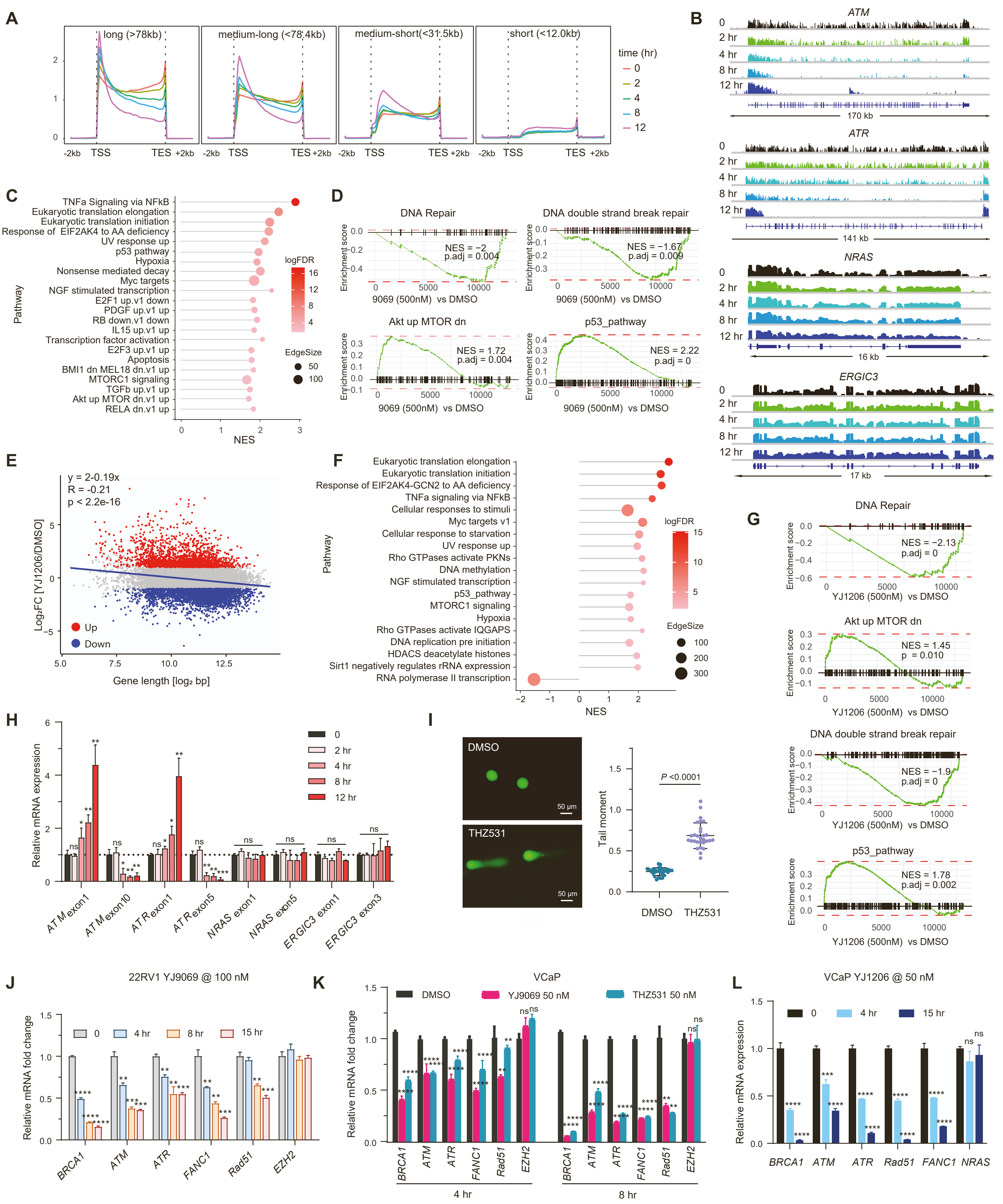

A
long (>78kb)
medium-long (<78.4kb)
medium-short(<31.5kb)
short (<12.0kb)
time (hr)
0
2
4
8
12
-2kb TSS TES +2kb
B
ATM
170 kb
ATR
141 kb
NRAS
16 kb
ERGIC3
17 kb
0
2 hr
4 hr
8 hr
12 hr
C
TNFa Signaling via NFkB
Eukaryotic translation elongation
Eukaryotic translation initiation
Response of EIF2AK4 to AA deficiency
UV response up
p53 pathway
Hypoxia
Nonsense mediated decay
Myc targets
NGF stimulated transcription
E2F1 up.v1 down
PDGF up.v1 up
RB down.v1 down
IL15 up.v1 up
Transcription factor activation
E2F3 up.v1 up
Apoptosis
BMI1 dn MEL18 dn.v1 up
MTORC1 signaling
TGFb up.v1 up
Akt up MTOR dn.v1 up
RELA dn.v1 up
Pathway
NES
logFDR
EdgeSize
D
DNA Repair
NES = −2
p.adj = 0.004
DNA double strand break repair
NES = −1.67
p.adj = 0.009
Akt up MTOR dn
NES = 1.72
p.adj = 0.004
p53_pathway
NES = 2.22
p.adj = 0
Enrichment score
9069 (500nM) vs DMSO
E
y = 2-0.19x
R = -0.21
p < 2.2e-16
Log₂FC [YJ1206/DMSO]
Gene length [log₂ bp]
Up
Down
F
Eukaryotic translation elongation
Eukaryotic translation initiation
Response of EIF2AK4-GCN2 to AA deficiency
TNFa signaling via NFkB
Cellular responses to stimuli
Myc targets v1
Cellular response to starvation
UV response up
Rho GTPases activate PKNs
DNA methylation
NGF stimulated transcription
p53_pathway
MTORC1 signaling
Hypoxia
Rho GTPases activate IQGAPS
DNA replication pre initiation
HDACS deacetylate histones
Sirt1 negatively regulates rRNA expression
RNA polymerase II transcription
Pathway
NES
logFDR
EdgeSize
G
DNA Repair
NES = −2.13
p.adj = 0
Akt up MTOR dn
NES = 1.45
p = 0.010
DNA double strand break repair
NES = −1.9
p.adj = 0
p53_pathway
NES = 1.78
p.adj = 0.002
YJ1206 (500nM) vs DMSO
H
Relative mRNA expression
0
2 hr
4 hr
8 hr
12 hr
ATM exon1
ATM exon10
ATR exon1
ATR exon5
NRAS exon1
NRAS exon5
ERGIC3 exon1
ERGIC3 exon3
I
DMSO
THZ531
50 μm
Tail moment
P <0.0001
J
22RV1 YJ9069 @ 100 nM
Relative mRNA fold change
0
4 hr
8 hr
15 hr
BRCA1
ATM
ATR
FANC1
Rad51
EZH2
K
VCaP
DMSO
YJ9069 50 nM
THZ531 50 nM
Relative mRNA fold change
4 hr
8 hr
L
VCaP YJ1206 @ 50 nM
Relative mRNA expression
0
4 hr
15 hr
BRCA1
ATM
ATR
Rad51
FANC1
NRAS

Figure S2. DNA repair and DNA double strand break repair pathways are downregulated upon CDK12/13 degradation. Related to Figure 2.
(A) Average metagene profiles for protein-coding genes stratified according to quartiles of gene length distribution in VCaP cells treated with YJ9069 (500 nM) at different time points.
(B) Sequence tracks of nascent transcript expression for long genes (top panel, *ATM* and *ATR*) and short genes (bottom panel, *NRAS* and *ERGIC3*) in VCaP cells treated with YJ9069 at different time points.
(C) Top KEGG pathway analyses of RNA-seq in VCaP cells upon CDK12/13 degradation by YJ9069 (500 nM) for 12 hours.
(D) Gene set enrichment analysis (GSEA) plots for the top rank-ordered dysregulated gene pathways in VCaP cells after YJ9069 treatment. NES, normalized enrichment score; p. adj, adjusted p-value.
(E) Scatter plot showing Log_2 fold changes in gene expression vs. Log_2 scale in gene length for each protein-coding gene in VCaP cells following treatment with YJ1206 at 500 nM for 12 hours ($p < 2.2e{-16}$, F-test). Differentially expressed genes are indicated (FDR < 0.05 and Log_2 FC > 1).
(F) Top KEGG pathway analyses of RNA-seq in VCaP cells upon CDK12/13 degradation by YJ1206 (500 nM) for 12 hours.
(G) Gene set enrichment analysis (GSEA) plots for the top rank-ordered dysregulated gene pathways in VCaP cells after YJ1206 treatment.
(H) Analysis of indicated gene expression by qPCR for nascent RNA after treatment with YJ1206 at different time points. Data are presented as mean +/− SD from n=3 independent experiments.
(I) Representative images of comet assay in VCaP cells after treatment with vehicle or THZ531 (100 nM) for 12 hours (scale=50 μm) (left panel) and quantification of tail moments (right panel). Boxplots represent interquartile ranges; horizontal bars denote the median. For each condition, 30 cells were analyzed.
(J-L) Analysis of indicated gene expression by qPCR at 4 hours, 8 hours, and/or 15 hours (as indicated in the figure) with YJ9069, THZ531, or YJ1206 in 22Rv1 or VCaP cells. Data are presented as mean +/− SD from n=3 independent experiments per condition.

Figure S3

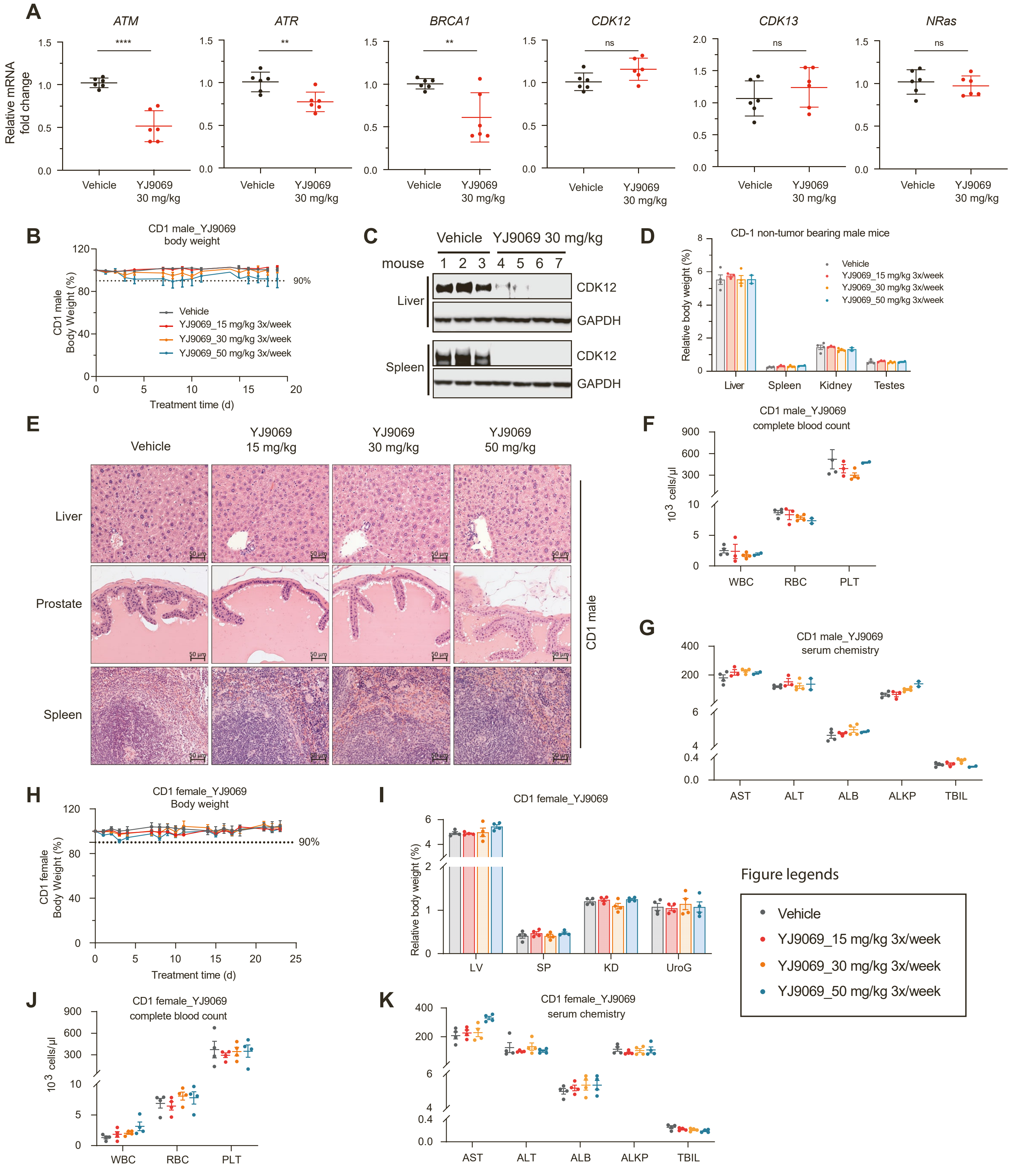
A
ATM
ATR
BRCA1
CDK12
CDK13
NRas
Relative mRNA fold change
****
**
**
ns
ns
ns
Vehicle
YJ9069 30 mg/kg
B
CD1 male_YJ9069 body weight
CD1 male Body Weight (%)
90%
Vehicle
YJ9069_15 mg/kg 3x/week
YJ9069_30 mg/kg 3x/week
YJ9069_50 mg/kg 3x/week
Treatment time (d)
C
Vehicle
YJ9069 30 mg/kg
mouse 1 2 3 4 5 6 7
Liver
Spleen
CDK12
GAPDH
D
CD-1 non-tumor bearing male mice
Relative body weight (%)
Liver
Spleen
Kidney
Testes
E
Vehicle
YJ9069 15 mg/kg
YJ9069 30 mg/kg
YJ9069 50 mg/kg
Liver
Prostate
Spleen
CD1 male
F
CD1 male_YJ9069 complete blood count
10^3 cells/μl
WBC
RBC
PLT
G
CD1 male_YJ9069 serum chemistry
AST
ALT
ALB
ALKP
TBIL
H
CD1 female_YJ9069 Body weight
CD1 female Body Weight (%)
90%
Treatment time (d)
I
CD1 female_YJ9069
Relative body weight (%)
LV
SP
KD
UroG
Figure legends
Vehicle
YJ9069_15 mg/kg 3x/week
YJ9069_30 mg/kg 3x/week
YJ9069_50 mg/kg 3x/week
J
CD1 female_YJ9069 complete blood count
10^3 cells/μl
WBC
RBC
PLT
K
CD1 female_YJ9069 serum chemistry
AST
ALT
ALB
ALKP
TBIL

Figure S3. YJ9069 demonstrates mild toxicity in CD1 male immune-competent mice. Related to Figure 3.
(A) Expression of indicated genes (qPCR) in tumors after treatment with YJ9069 (i.v., 30 mg/kg, 3x/week) for 5 days in castrated VCaP xenografts. Data are presented as mean values ± SD of triplicate points (n = 6 per condition). $*p < 0.05$, $**p < 0.01$, $***p < 0.001$, $****p < 0.0001$ by t-test.
(B) Body weight percentage of the vehicle and YJ9069 groups at noted doses in CD-1 male mice throughout the treatment period (two-sided t-test). Data are presented as mean ± SEM (n = 4, biological replicates).
(C) Immunoblot of CDK12 from host organs in the CD1 male mouse after 5 days treatment with YJ9069 (i.v., 30 mg/kg, 3x/week). GAPDH is the loading control.
(D) Major organ weight measurement for vehicle and YJ9069 groups at noted doses in CD-1 male mouse. Data are presented as mean ± SEM (n = 4, biological replicates).
(E) Representative H&E staining for liver, spleen, and prostate from different groups of the CD-1 male toxicity study (scale=50 μm).
(F-G) Complete blood counts (F) and serum chemistry (G) for the vehicle and YJ9069 groups at noted doses in CD-1 male mice. WBC, white blood cells; RBC, red blood cells; PLT, platelets. Data are presented as mean ± SD (n = 4, biological replicates).
(H-K) is the same as Fig. S3B, S3D, and S3F-G, except in CD-1 female mice (n = 4 per condition).

Figure S4

A

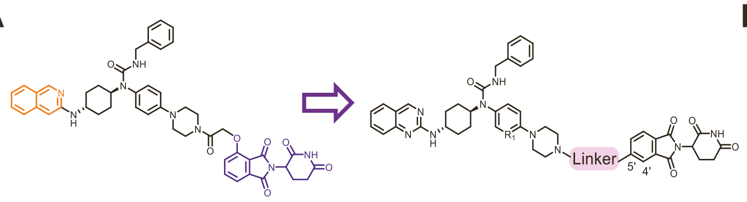

YJ9069

B

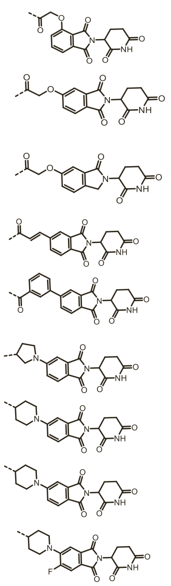

Compds	Linker + E3 ligand	R_1	VCaP IC_{50} (nM)
YJ9069		CH	22.90
YJ1090		CH	93.85
YJ1094		CH	25.21
YJ1114		CH	2025
YJ1130		CH	125.5
YJ1096		CH	97.75
YJ1105		CH	15.11
YJ1205		N	3.93
YJ1206		N	13.50

C

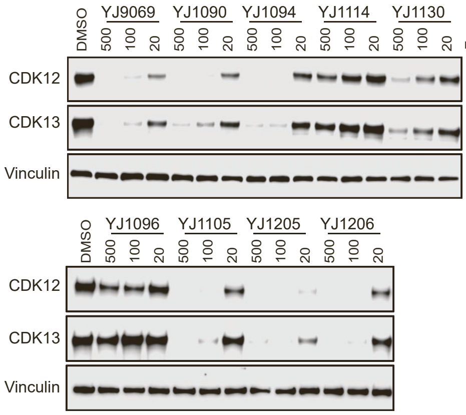

D

Parameter	YJ9069 (mg/kg)		YJ1105		YJ1205		YJ1206	
	i.v. (2.5)	Oral (10)	i.v. (2.5)	Oral (10)	i.v. (2.5)	Oral (10)	i.v. (2.5)	Oral (10)
$T_{1/2}$ (h)	4.39	NA	7.05	7.2	3.81	4.2	5.1 5	6. 24
T_{max} (h)	0.08	1.56	0.08	6	0.08	4.7	0.08	6.0
C_{max} (μM)	4.13	0.006	1.58	0.715	4.14	0.66	3.49	1.26
AUC(0 -t)(h *ng/mL)	2929.22	5.06	8039.69	8567.43	10519.6	6679.0	9463.33	14824.81
AUC(0 -∞)(h*ng/mL)	2968.30	NA	87 41 .34	9805.4	10615.68	6842.8	9763.68	16205.27
CL(mL/h/kg)	848.10	NA	288.16	NA	235.84	NA	257.41	NA
F(%)		0.04%		26.64%		15.87	39.16%	

E

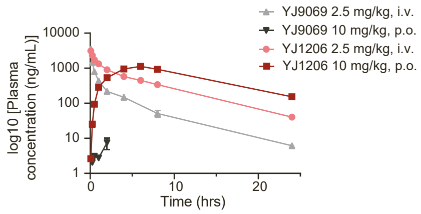

F

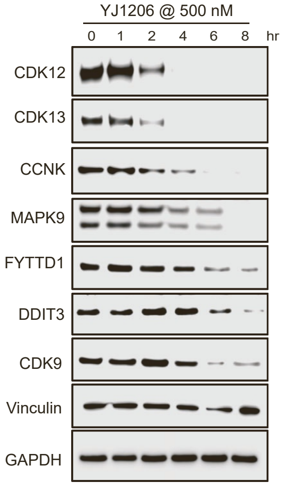

G

Gene name	Log₂(fold change: YJ1206/DMSO)	*P*-value
CDK12	-3.18	1E-17
CDK13	-3.17	1E-17
CDK1	-0.14	0.0070945
CDK2	0.02	0.7635315
CDK4	-0.25	0.0096923
CDK5	0.03	0.702421
CDK6	0.12	0.1139111
CDK7	-0.18	0.286255
CDK8	0.04	0.6896741
CDK9	-1.63	1E-17
CDK10	-0.18	0.3721337
CDK14	-0.16	0.5010827
CDK16	0.1	0.2626574
CDK17	-0.22	0.1593374
CDK18	0.01	0.8142111

H

Gene name	Log₂(fold change: YJ1206/DMSO)	*P*-value
CDK12	-3.18	1E-17
CDK13	-3.17	1E-17
FYTTD1	-2.96	1E-17
DDIT3	-2.78	1E-17
MAPK9	-2.29	1E-17
CCNK	-2.28	1E-17
FOS	3.62	1E-17
JUNB	2.48	1E-17

I

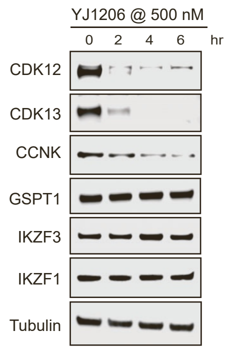

Figure S4. An oral available degrader, YJ1206, is developed and optimized with high specificity towards CDK12/13. Related to Figure 4.
(A-B) Chemical optimization of the linker from YJ9069 to oral CDK12/13 degraders (IC_{50} values are calculated from n = 3 independent experiments).
(C) Immunoblots of CDK12 and CDK13 in VCaP cells treated with synthesized CDK12/13 degraders for 4 hours.
(D) Pharmacokinetic profiles of YJ9069, YJ1105, YJ1205, and YJ1206 following intravenous (i.v., 2.5 mg/kg) and oral (p.o., 10 mg/kg) injection in Sprague Dawley (SD) rats.
(E) Plasma concentration time curve of YJ1206 and YJ9069 with intravenous (i.v., 2.5 mg/kg) and oral (p.o., 10 mg/kg) injection in SD rats (n = 3 per condition).
(F) Immunoblots of the down-regulated proteins shown in Fig. S4H in 22Rv1 cells treated with YJ1206 at different time points.
(G) Proteomic analysis of CDK family proteins in 22Rv1 cells treated with YJ1206 (500 nM) versus DMSO.
(H) The top up- and down-regulated proteins from the global proteomic profile study.
(I) Immunoblots of CDK12, CDK13, CCNK, and neo-substrates in 22Rv1 cells treated with YJ1206 (500 nM) at increasing time durations. Tubulin is used as a loading control.

Figure S5

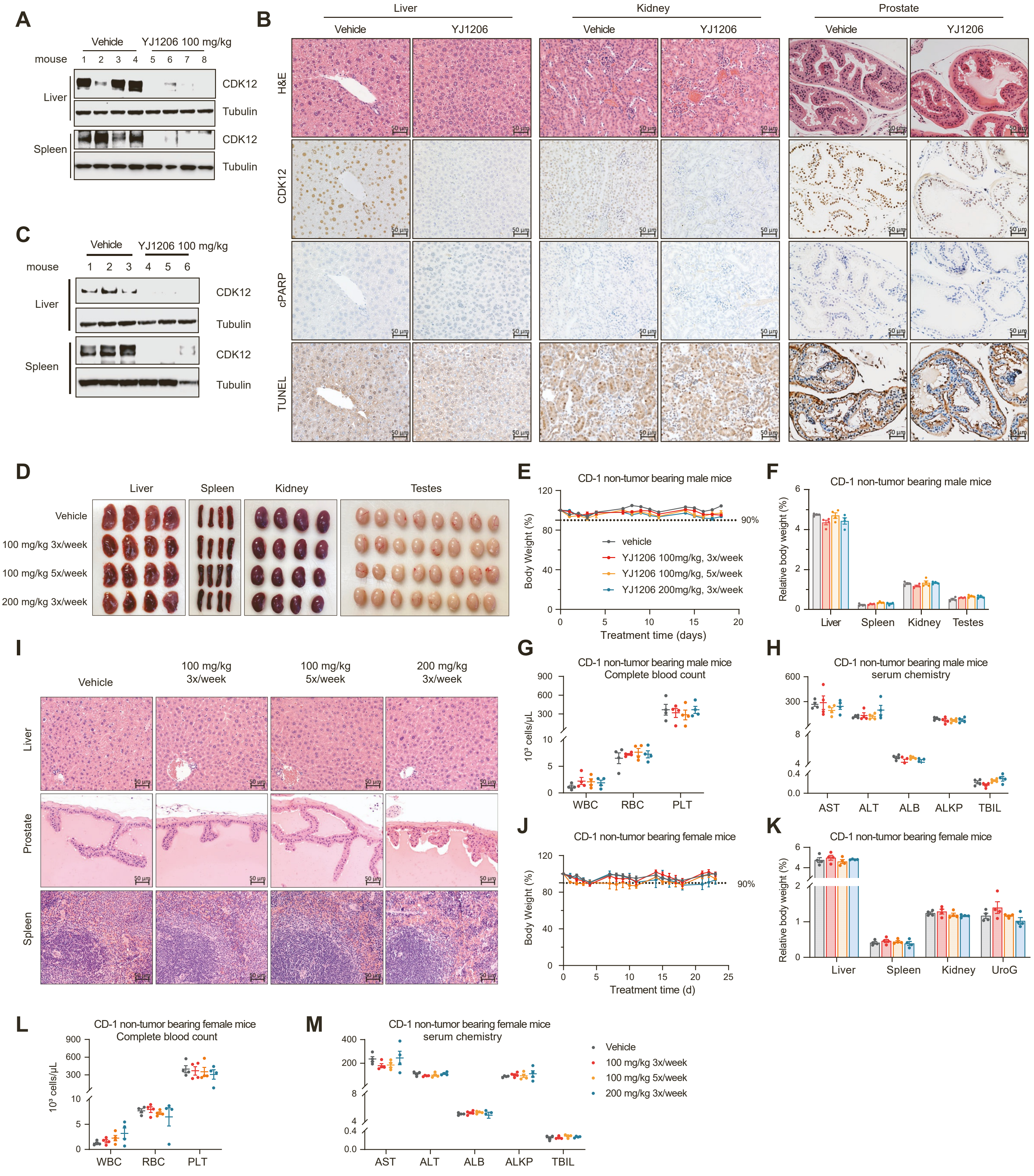
A
Vehicle
YJ1206 100 mg/kg
mouse
1 2 3 4 5 6 7 8
Liver
Spleen
CDK12
Tubulin
B
Liver
Kidney
Prostate
Vehicle
YJ1206
H&E
CDK12
cPARP
TUNEL
C
mouse
1 2 3 4 5 6
D
Liver
Spleen
Kidney
Testes
Vehicle
100 mg/kg 3x/week
100 mg/kg 5x/week
200 mg/kg 3x/week
E
CD-1 non-tumor bearing male mice
Body Weight (%)
Treatment time (days)
90%
vehicle
YJ1206 100mg/kg, 3x/week
YJ1206 100mg/kg, 5x/week
YJ1206 200mg/kg, 3x/week
F
Relative body weight (%)
Liver
Spleen
Kidney
Testes
G
Complete blood count
10³ cells/μL
WBC
RBC
PLT
H
serum chemistry
AST
ALT
ALB
ALKP
TBIL
I
100 mg/kg 3x/week
100 mg/kg 5x/week
200 mg/kg 3x/week
Liver
Prostate
Spleen
J
CD-1 non-tumor bearing female mice
Treatment time (d)
K
UroG
L
M
Vehicle
100 mg/kg 3x/week
100 mg/kg 5x/week
200 mg/kg 3x/week

Figure S5. YJ1206 shows no evidence of toxicity in both male and female immuno-competent CD1 mice. Related to Figure 4.

(A) Immunoblot of CDK12 from host organs in the VCaP-CRPC xenograft model after 5 days treatment with YJ1206 (p.o., 100 mg/kg, 3x/week). Tubulin is the loading control.

(B) Representative H&E staining and immunohistochemistry of CDK12, cleaved PARP, and TUNEL for host organs (scale=50 μm).

(C) Immunoblot of CDK12 from host organs in CD1 male model after 5 days treatment with YJ1206 (p.o., 100 mg/kg, 3x/week). Tubulin is the loading control.

(D) Images for liver, spleen, kidney, and testes from CD1 male model treated with YJ1206 at different doses.

(E) Percent body weight of the vehicle and YJ1206 groups from CD1 male mice throughout the treatment period. Data are presented as mean ± SEM (n = 4 per condition).

(F) Major organ weight of the vehicle and YJ1206 groups at noted doses in CD-1 male mouse. Data are presented as mean ± SEM (n = 4, biological replicates).

(G-H) Complete blood counts and serum chemistry of the vehicle and YJ1206 groups at noted doses in CD-1 male mice. WBC, white blood cells; RBC, red blood cells; PLT, platelets. Data are presented as mean ± SD (n = 4, biological replicates).

(I) Representative H&E staining for liver, spleen, and prostate of the CD-1 male toxicity study of YJ1206 (scale=50 μm).

(J-M) same as in panels E-H, except in CD1 female mice (n = 4 per condition).

Figure S6

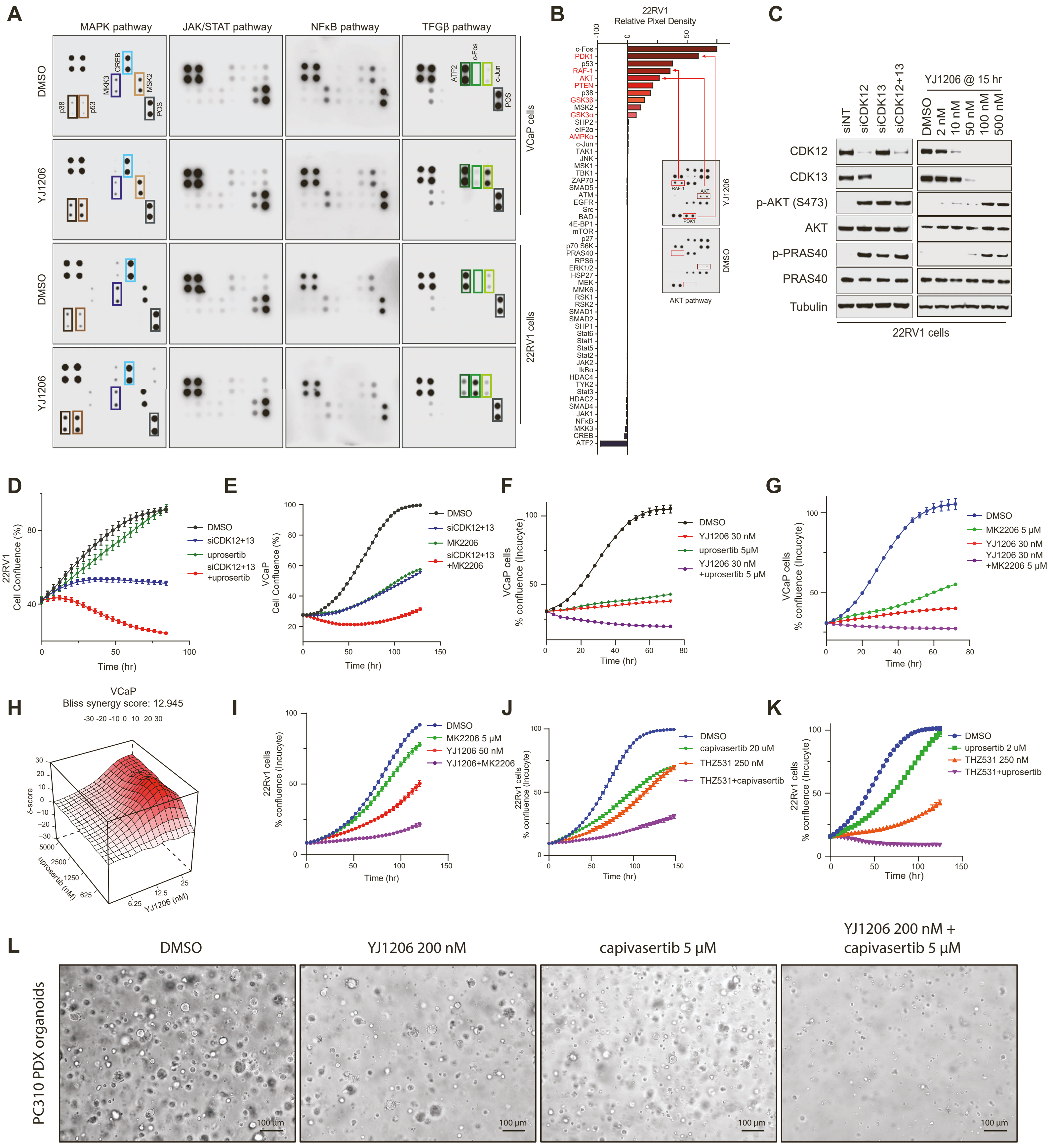
A
MAPK pathway
JAK/STAT pathway
NFκB pathway
TFGβ pathway
DMSO
YJ1206
VCaP cells
22RV1 cells
p38
p53
MKK3
CREB
MSK2
POS
ATF2
c-Fos
c-Jun
B
22RV1
Relative Pixel Density
c-Fos
PDK1
p53
RAF-1
AKT
PTEN
p38
GSK3β
MSK2
GSK3α
SHP2
eIF2α
AMPKα
c-Jun
TAK1
JNK
MSK1
TBK1
ZAP70
SMAD5
ATM
EGFR
Src
BAD
4E-BP1
mTOR
p27
p70 S6K
PRAS40
RPS6
ERK1/2
HSP27
MEK
MMK6
RSK1
RSK2
SMAD1
SMAD2
SHP1
Stat6
Stat1
Stat5
Stat2
JAK2
IkBα
HDAC4
TYK2
Stat3
HDAC2
SMAD4
JAK1
NFκB
MKK3
CREB
ATF2
AKT pathway
C
siNT
siCDK12
siCDK13
siCDK12+13
YJ1206 @ 15 hr
DMSO
2 nM
10 nM
50 nM
100 nM
500 nM
CDK12
CDK13
p-AKT (S473)
AKT
p-PRAS40
PRAS40
Tubulin
22RV1 cells
D
22RV1 Cell Confluence (%)
Time (hr)
DMSO
siCDK12+13
uprosertib
siCDK12+13 +uprosertib
E
VCaP Cell Confluence (%)
Time (hr)
DMSO
siCDK12+13
MK2206
siCDK12+13 +MK2206
F
VCaP cells % confluence (Incucyte)
Time (hr)
DMSO
YJ1206 30 nM
uprosertib 5μM
YJ1206 30 nM +uprosertib 5 μM
G
VCaP cells % confluence (Incucyte)
Time (hr)
DMSO
MK2206 5 μM
YJ1206 30 nM
YJ1206 30 nM +MK2206 5 μM
H
VCaP
Bliss synergy score: 12.945
δ-score
uprosertib (nM)
YJ1206 (nM)
I
22Rv1 cells % confluence (Incucyte)
Time (hr)
DMSO
MK2206 5 μM
YJ1206 50 nM
YJ1206+MK2206
J
22Rv1 cells % confluence (Incucyte)
Time (hr)
DMSO
capivasertib 20 uM
THZ531 250 nM
THZ531+capivasertib
K
22Rv1 cells % confluence (Incucyte)
Time (hr)
DMSO
uprosertib 2 uM
THZ531 250 nM
THZ531+uprosertib
L
DMSO
YJ1206 200 nM
capivasertib 5 μM
YJ1206 200 nM + capivasertib 5 μM
PC310 PDX organoids
100 μm

Figure S6. YJ1206 induces phosphorylation of AKT and exhibits synthetic lethality with AKT inhibitors *in vitro*. Related to Figure 5.

(A) Representative blots of human phosphorylation pathway profiling array for MAPK, JAK/STAT, NFκB, and TGFβ pathways in VCaP or 22Rv1 cells treated with or without YJ1206 for 15 hours at a concentration of 500 nM.

(B) Human phosphorylation pathway profiling array analysis of 22Rv1 cells treated with YJ1206 (500 nM) for 15 hours.

(C) Immunoblot of the noted proteins in 22Rv1 cells treated with YJ1206 at increasing concentrations, or with siRNA targeting CDK12 and/or CDK13. Tubulin is the loading control.

(D-E) Real-time growth curves of 22Rv1 or VCaP cells upon treatment with siCDK12/13 and/or AKT inhibitor, uprosertib or MK2206. Data are presented as mean +/− SD from n = 3 independent experiments.

(F-G) Real-time growth curves of VCaP cells upon treatment with YJ1206 and/or uprosertib or MK2206. Data are presented as mean +/− SD from n = 3 independent experiments.

(H) VCaP cells were treated with YJ1206 and/or uprosertib at varying concentrations to determine the effect on cell growth and drug synergism, with assessments using the Bliss method. Red peaks in the 3D plots denote synergy, and the average synergy score is noted above the plot.

(I-K) Real-time growth curves of 22Rv1 cells upon treatment with YJ1206 or THZ531 and/or AKT inhibitors, uprosertib, MK2206, or capivasertib. Data are presented as mean +/− SD from n = 3 independent experiments.

(L) Images of PC310 PDX organoids after treatment with YJ1206 and/or capivasertib for 5 days (scale=50 μm).

Figure S7

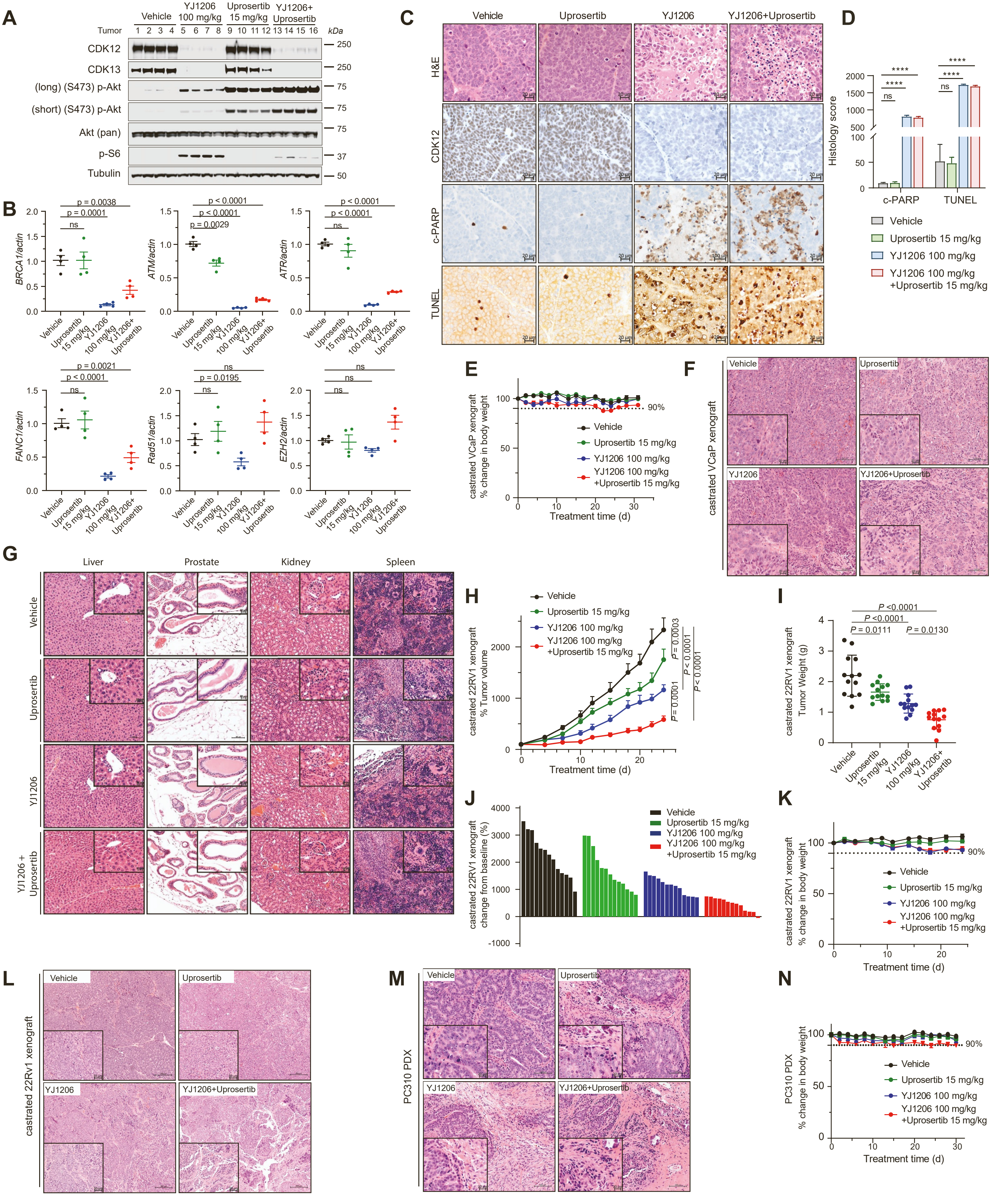

A
Vehicle
YJ1206 100 mg/kg
Uprosertib 15 mg/kg
YJ1206+ Uprosertib
Tumor 1 2 3 4 5 6 7 8 9 10 11 12 13 14 15 16
kDa
CDK12
CDK13
(long) (S473) p-Akt
(short) (S473) p-Akt
Akt (pan)
p-S6
Tubulin
250
250
75
75
75
37
50
B
BRCA1/actin
ATM/actin
ATR/actin
FANC1/actin
Rad51/actin
EZH2/actin
p = 0.0038
p = 0.0001
p < 0.0001
p = 0.0029
p = 0.0021
p = 0.0195
ns
Vehicle
Uprosertib 15 mg/kg
YJ1206 100 mg/kg
YJ1206+ Uprosertib
C
Vehicle
Uprosertib
YJ1206
YJ1206+Uprosertib
H&E
CDK12
c-PARP
TUNEL
D
Histology score
c-PARP
TUNEL
****
ns
Vehicle
Uprosertib 15 mg/kg
YJ1206 100 mg/kg
YJ1206 100 mg/kg +Uprosertib 15 mg/kg
E
castrated VCaP xenograft % change in body weight
90%
Treatment time (d)
F
castrated VCaP xenograft
G
Liver
Prostate
Kidney
Spleen
YJ1206 + Uprosertib
H
castrated 22RV1 xenograft % Tumor volume
P= 0.0003
P < 0.0001
P = 0.0001
Treatment time (d)
I
castrated 22RV1 xenograft Tumor Weight (g)
P <0.0001
P = 0.0111
P = 0.0130
J
castrated 22RV1 xenograft change from baseline (%)
K
castrated 22RV1 xenograft % change in body weight
L
castrated 22Rv1 xenograft
M
PC310 PDX
N
PC310 PDX % change in body weight

Figure S7. The combinatorial regimen of CDK12/13 degraders with AKT inhibitors suppresses tumor growth *in vivo.* Related to Figure 6.

(A) Immunoblot of the noted proteins from castrated VCaP xenograft tumors after 5 days of treatment with YJ1206 (p.o., 100 mg/kg, 3x/week). Tubulin is the loading control.

(B) Expression of the indicated gene by qPCR in tumors from panel A. Data are presented as mean values ± SD (n = 4 per condition). $*p \leq 0.05$, $**p \leq 0.01$, $***p \leq 0.001$, $****p \leq 0.0001$ by t test.

(C) Representative H&E staining and immunohistochemistry of CDK12, cleaved PARP, and TUNEL for the VCaP CRPC xenograft tumors (scale=20 μm).

(D) Histology score for immunohistochemistry of cleaved PARP and TUNEL in the castrated VCaP xenograft tumors.

(E) Percentage of body weight for VCaP-CRPC throughout the treatment period. Data are presented as mean ± SEM (n = 20 per condition).

(F) Representative tumor images from the vehicle, uprosertib, YJ1206, and combinatorial regimen in castrated VCaP CDX models (scale=200 μm). The inset scale=20 μm.

(G) H&E staining of normal organs from the vehicle, uprosertib, YJ1206, and combinatorial regimen in castrated VCaP CDX (scale=200 μm). The inset scale=20 μm.

(H) Tumor volume of 22Rv1 model with YJ1206 (p.o., 100 mg/kg, 3x/week) alone or in combination with uprosertib (p.o., 15 mg/kg, 5x/week) treatment. Data are mean ± SEM (n = 12 per condition; two-sided t-test).

(I) Tumor weights from castrated 22Rv1 xenograft study (two-sided t-test). Data are presented as mean ± SEM.

(J) Waterfall plot depicting the change in tumor volume after 24 days of treatment from 22Rv1 study.

(K) Percent body weight for 22Rv1 CRPC model throughout the treatment period. Data are presented as mean ± SEM.

(L-M) Representative H&E staining for 22Rv1 CRPC xenograft or PC310 PDX tumors at the end point (scale=200 μm). The inset scale=20 μm.

(N) Percent body weight for PC310 PDX throughout the treatment period. Data are presented as mean ± SEM (n = 6 per condition).

Table S1. Detailed oligonucleotides information. Related to STAR Methods and Key resources table.

Oligonucleotides	SOURCE	IDENTIFIER
*FANCI*_Fwd: CACCACACTTACAGCCCTTG	Quereda, V. et al[16]	N/A
*FANCI*_Rev: ATTCCTCCGGAGCTCTGAC	Quereda, V. et al[16]	N/A
*NRAS*_Fwd: GCGAAGGCTTCCTCTGTGTA	Quereda, V. et al[16]	N/A
*NRAS*_Rev: CTTGTTTCCCACTAGCACCA	Quereda, V. et al[16]	N/A
*RAD51*_Fwd: GCTGATGAGTTTGGTGTAGCAG	Quereda, V. et al[16]	N/A
*RAD51*_Rev: GGAAGACAGGGAGAGTCGTAGA	Quereda, V. et al[16]	N/A
*EZH2*_Fwd: GACCTCTGTCTTACTTGTGGAGC	This paper	N/A
*EZH2*_Rev: CGTCAGATGGTGCCAGCAATAG	This paper	N/A
*ACTB*_Fwd: AGGATGCAGAAGGAGATCACTG	This paper	N/A
*ACTB*_Rev: AGTACTTGCGCTCAGGAGGAG	This paper	N/A
*ATM exon1*_Fwd: GACCGCGTGATACTGGATG	This paper	N/A
*ATM exon1*_Rev: TCAAACCCTGCGTGACTG	This paper	N/A
*ATM exon10*_Fwd: GGCATAAATATTCCAGCAGACC	This paper	N/A
*ATM exon10*_Rev: TTCCATAGTAGGGACAACAACA	This paper	N/A
*ATR exon1*_Fwd: CCTGGGTCCTGCATCCT	This paper	N/A
*ATR exon1*_Rev: CAGCCATAGCGCAGCAG	This paper	N/A
*ATR exon5*_Fwd: CTACCAAAGTCAGCAGCTTTATC	This paper	N/A
*ATR exon5*_Rev: CATGATGTAGGATCAGGGAATGT	This paper	N/A
*NRAS exon1*_Fwd: GCTGTTCATGGCGGTTC	This paper	N/A
*NRAS exon1*_Rev: CTCAAGCTCCACTGCCT	This paper	N/A
*NRAS exon5*_Fwd: GTTCTTCCACAGCACAAACAC	This paper	N/A
*NRAS exon5*_Rev: ATCACCAGCAGTTGCTACTTTA	This paper	N/A
*ERGIC3 exon1*_Fwd: CACGAGCCATCCAGAAGAAA	This paper	N/A
*ERGIC3 exon1*_Rev: AGGGAGAAAGAGGAGACAGAG	This paper	N/A
*ERGIC3 exon3*_Fwd: TGCTTCCTCTGTCTCCTCTT	This paper	N/A
*ERGIC3 exon3*_Rev: CTGACCGAGGAGGTGGA	This paper	N/A
siRNA targeting sequence CDK12: CUACAGAGCGACUUCCUUA	Horizon Discovery	J-004031-10-0050
siRNA targeting sequence CDK13: GCUGAUAGCUUACGAGGAA	Horizon Discovery	J-004688-06-0050

Method S1. Chemical structures and synthesis of CDK12/13 degraders and analogues. Related to Figure 1 and Figure 4.

General information: All commercially available reagents and solvents were used without further purification. All chemical reactions were monitored by thin-layer chromatography (TLC) plates with visualization under UV light (254 or 365 nm). ^{1}H NMR spectra were performed with Bruker AV-400/600 spectrometer, and ^{13}C NMR spectra were recorded on Bruker AV-600 spectrometer at 150 MHz; internal reference was either TMS or deuterated NMR solvent. Low-resolution mass spectra (MS) were recorded on an Agilent 1200 HPLC-MSD mass spectrometer. High resolution mass spectral analysis was recorded on an Applied Biosystems Q-STAR Elite ESI-LC-MS/MS mass spectrometer. Purity of all final compounds was confirmed to be >95% by HPLC analysis with the Agilent 1260 system. The analytical columns were YMC-Triart C18 reversed-phase column, 5 μm, 4.6 mm × 250 mm, and flow rate 1.0 mL/min.

Scheme S1. Synthetic route of YJ9068, YJ9069, YJ1090, YJ1094, YJ1114, YJ1130, YJ1096, YJ1105, YJ1205, YJ1206 and YJ1078[a]

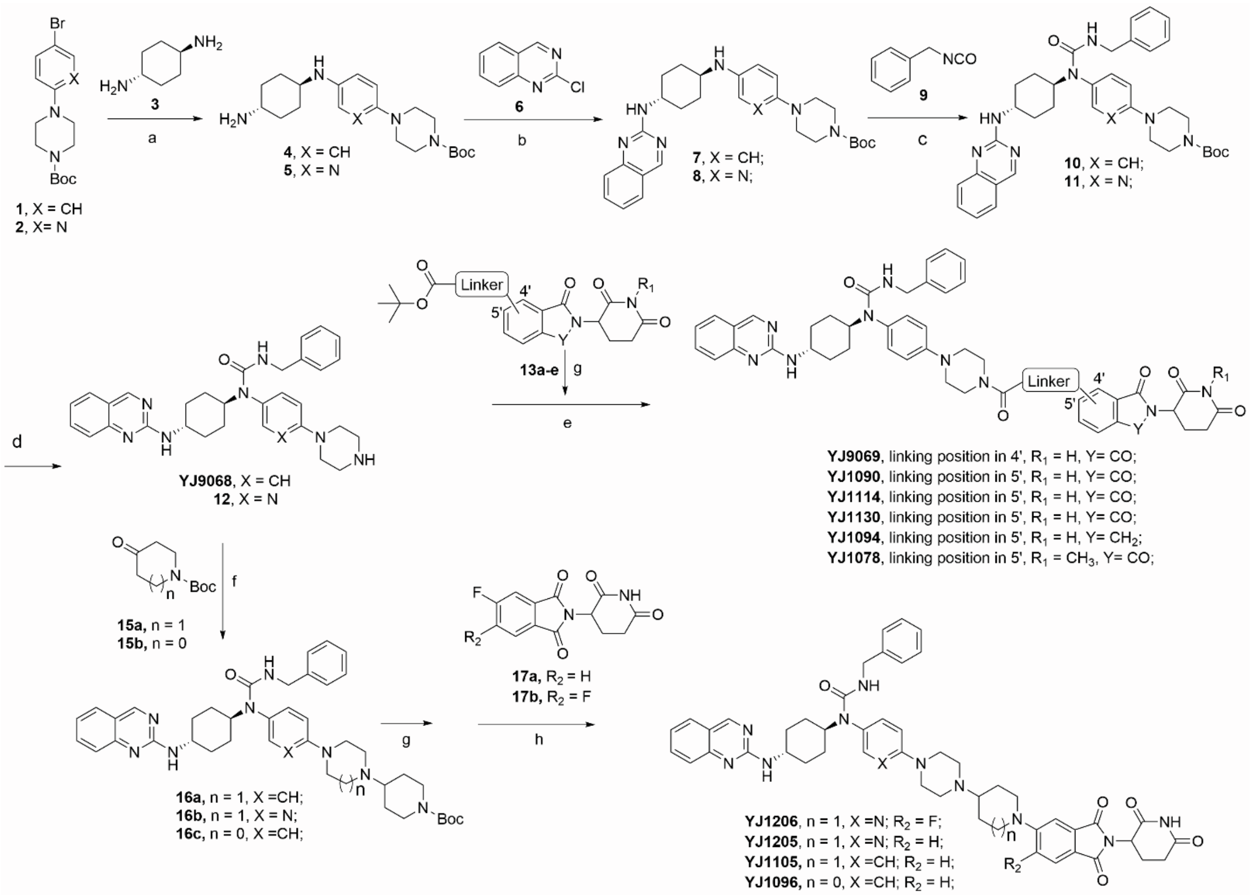

[a]Reagents and conditions: (a) *D*-proline, CuI, K_3PO_4, dry dimethyl sulfoxide (DMSO), 100 °C, 10 h, 44%; (b) Cs_2CO_3, *N,N*-dimethylformamide (DMF), 60 °C, 3 h, 90%; (c) *N,N*-diisopropylethylamine (DIPEA), DMF, 95 °C, 4 h, 62%; (d) TFA, DCM, 4 h, rt, 79%; (e) 2-(7-Azabenzotriazol-1-yl)-*N,',N',N'*-tetramethyluronium hexafluorophosphate (HATU), DIPEA, DMF, rt, 15 min, 75%; (f) DCM, Sodium triacetoxyborohydride, rt 52%; (g) TFA, DCM, 4 h, rt; (h) DMSO, DIPEA, 120 °C, 8 h, 63%.

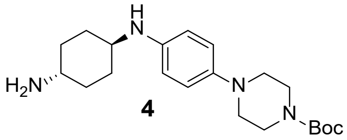

4

tert-butyl 4-(4-(((1r,4r)-4-aminocyclohexyl)amino)phenyl)piperazine-1-carboxylate (4)

Potassium phosphate (31.0 g, 146 mmol) was added to a solution of *trans*-cyclohexane-1,4-diamine (29.3 g, 256.4 mmol), *tert*-butyl 4-(4-bromophenyl)piperazine-1-carboxylate **3** (25.0 g, 73.26 mmol), CuI (1.4 g, 7.3 mmol), and *D*-Proline (843 mg, 7.3 mmol) in anhydrous DMSO (500 mL). The resulted suspension was then evacuated and backfilled with argon (3 cycles). The reaction mixture was then heated at 100 °C for 10 hours before being filtered through celite. The reaction solvent was evaporated under reduced pressure and purified by silica gel column chromatography to afford the title compound as a gray solid. (12.0 g, yield 44%): ^{1}H NMR (400 MHz, DMSO-d_6) δ 6.74 (d, J = 8.8 Hz, 2H), 6.49 (d, J = 8.9 Hz, 2H), 4.88 (d, J = 8.2 Hz, 1H), 3.42 (t, J = 5.1 Hz, 4H), 3.03 (s, 1H), 2.83 (t, J = 5.1 Hz, 4H), 2.76 (s, 1H), 1.95 (d, J = 12.8 Hz, 2H), 1.85 (d, J = 12.4 Hz, 2H), 1.41 (s, 9H), 1.26 (q, J = 10.9 Hz, 2H), 1.11 (q, J = 11.6 Hz, 2H). HRMS (ESI) for $C_{21}H_{34}N_4O_2$ $[M+H]^+$, calcd: 375.2755, found: 375.2739.

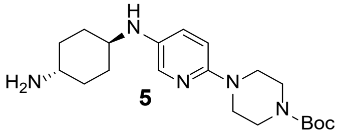

5

tert-butyl 4-(5-(((1r,4r)-4-aminocyclohexyl)amino)pyridin-2-yl)piperazine-1-carboxylate (5)

Compound **5** was synthesized by following a similar procedure as that of Compound **4**. ^{1}H NMR (400 MHz, DMSO-d_6) δ 7.60 (s, 1H), 6.94 (d, J = 8.9 Hz, 1H), 6.68 (d, J = 8.9 Hz, 1H), 4.83 (d, J = 8.5 Hz, 1H), 3.40 (t, J = 4.8 Hz, 4H), 3.19 (t, J = 4.8 Hz, 4H), 3.08 – 2.95 (m, 2H), 2.62 – 2.53 (m, 1H), 1.95 – 1.85 (m, 2H), 1.82 – 1.70 (m, 2H), 1.42 (s, 9H), 1.12 (q, J = 11.3 Hz, 4H).

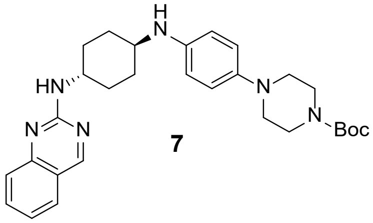

7

tert-butyl 4-(4-(((1r,4r)-4-(quinazolin-2-ylamino)cyclohexyl)amino)phenyl) piperazine-1-carboxylate (7)

To a solution of *tert*-butyl 4-(4-(((1r,4r)-4-aminocyclohexyl)amino)phenyl)piperazine-1-carboxylate **4** (9.0 g, 24 mmol) in DMF (50 mL) was added 2-chloroquinazoline (3.95 g, 24 mmol), Cs_2CO_3 (9.4 g, 28.9 mmol). The mixture was stirred at room temperature overnight. The reaction mixture was then filtered, and the solvent was removed under reduced pressure. The crude material was purified by column chromatography to afford **7** as white solid (10.8 g, yield 90%). ^{1}H NMR (400 MHz, DMSO-d_6) δ 9.09 (s, 1H), 7.77 (d, J = 8.0 Hz, 1H), 7.67 (t, J = 7.7 Hz, 1H), 7.45 (d, J = 8.5 Hz, 1H), 7.29 (d, J = 8.0 Hz, 1H), 7.20 (t, J = 7.4 Hz, 1H), 6.76 (d, J = 8.2 Hz, 2H), 6.52 (d, J = 8.3 Hz, 2H), 4.95 (s, 1H), 3.86 (d, J = 9.8 Hz, 1H), 3.43 (t, J = 5.0 Hz, 4H), 3.18 – 3.01 (m, 1H), 2.84 (s, 4H), 2.01 (d, J = 11.6 Hz, 4H), 1.50 – 1.32 (m, 11H), 1.30 – 1.15 (m, 2H).

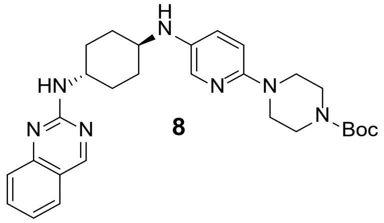

8

tert-butyl 4-(5-(((1r,4r)-4-(quinazolin-2-ylamino)cyclohexyl)amino)pyridin-2-yl)piperazine-1-carboxylate (8)

Compound **8** was synthesized by following a similar procedure as that of Compound **7**. ^{1}H NMR (400 MHz, DMSO-d_6) δ 9.09 (s, 1H), 7.77 (d, *J* = 8.0 Hz, 1H), 7.70 – 7.61 (m, 2H), 7.45 (d, *J* = 8.6 Hz, 1H), 7.29 (d, *J* = 7.9 Hz, 1H), 7.20 (t, *J* = 7.5 Hz, 1H), 6.98 (d, *J* = 8.6 Hz, 1H), 6.70 (d, *J* = 8.9 Hz, 1H), 4.92 (s, 1H), 3.93 – 3.80 (m, 1H), 3.40 (t, *J* = 5.4 Hz, 4H), 3.21 (t, *J* = 5.4 Hz, 4H), 3.17 – 3.06 (m, 1H), 2.02 (d, *J* = 12.1 Hz, 4H), 1.49 – 1.35 (m, 11H), 1.24 (q, *J* = 13.2, 12.4 Hz, 2H).

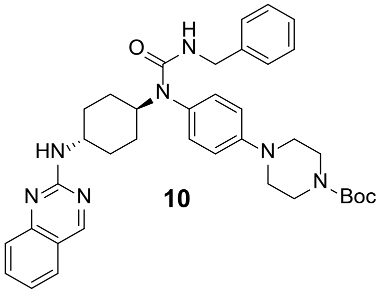

10

tert-butyl 4-(4-(3-benzyl-1-((1r,4r)-4-(quinazolin-2-ylamino)cyclohexyl)ureido) phenyl)piperazine-1-carboxylate (10)

To a solution of *tert*-butyl 4-(4-(((1r,4r)-4-(quinazolin-2-ylamino)cyclohexyl)amino)phenyl)piperazine-1-carboxylate **7** (10.0 g, 19.89 mmol) and DIPEA (7.7 g, 59.67 mmol) in DMF (20 mL) was added benzyl isocyanate (7.94 g, 59.67 mmol) at room temperature. The mixture was stirred at 95 °C for 4 hours. The solvent was removed under reduced pressure and purified by column chromatography to give a white solid (7.8 g, yield 62%). ^{1}H NMR (400 MHz, DMSO-d_6) δ 9.05 (s, 1H), 7.74 (d, *J* = 8.0 Hz, 1H), 7.63 (t, *J* = 7.8 Hz, 1H), 7.40 (d, *J* = 8.8 Hz, 1H), 7.31 – 7.22 (m, 3H), 7.21 – 7.13 (m, 4H), 7.08 – 6.98 (m, 4H), 5.58 (t, *J* = 6.1 Hz, 1H), 4.28 (t, *J* = 12.3 Hz, 1H), 4.16 (d, *J* = 5.8 Hz, 2H), 3.58 (s, 1H), 3.47 (t, *J* = 5.1 Hz, 4H), 3.18 (t, *J* = 5.2 Hz, 4H), 1.96 (d, *J* = 12.0 Hz, 2H), 1.79 (d, *J* = 12.3 Hz, 2H), 1.50 – 1.33 (m, 12H), 1.13 (q, *J* = 13.5, 12.5 Hz, 2H).

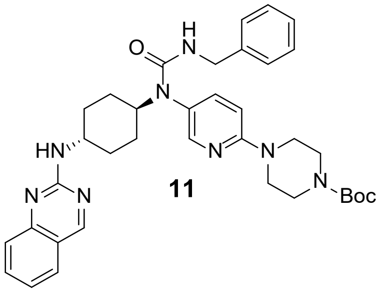

11

tert-butyl 4-(5-(3-benzyl-1-((1r,4r)-4-(quinazolin-2-ylamino)cyclohexyl)ureido) pyridin-2-yl)piperazine-1-carboxylate (11)

Compound **11** was synthesized by following a similar procedure as that of Compound **10**. ^{1}H NMR (400 MHz, DMSO-d_6) δ 9.05 (s, 1H), 7.93 (s, 1H), 7.74 (d, *J* = 8.0 Hz, 1H), 7.63 (t, *J* = 7.8 Hz, 1H), 7.39 (t, *J* = 9.1 Hz, 2H), 7.31 – 7.20 (m, 3H), 7.20 – 7.13 (m, 4H), 6.91 (d, *J* = 8.9 Hz, 1H), 6.05 (t, *J* = 6.1 Hz, 1H), 4.28 (t, *J* = 12.5 Hz, 1H), 4.15 (d, *J* = 6.0 Hz, 2H), 3.66 – 3.49 (m, 5H), 3.45 (t, *J* = 5.1 Hz, 4H), 2.07 – 1.89 (m, 2H), 1.79 (d, *J* = 12.3 Hz, 2H), 1.50 – 1.33 (m, 11H), 1.09 (q, *J* = 12.5 Hz, 2H).

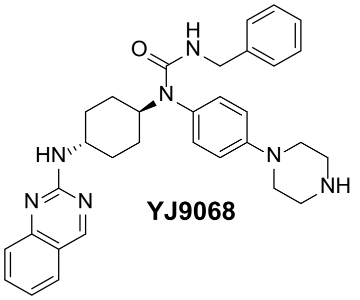

YJ9068

3-benzyl-1-(4-(piperazin-1-yl)phenyl)-1-((1r,4r)-4-(quinazolin-2-ylamino)cyclohexyl) urea (YJ9068)

TFA (7 mL) was added to a solution of *tert*-butyl 4-(4-(3-benzyl-1-((1r,4r)-4-(quinazolin-2-ylamino)cyclohexyl)ureido)phenyl)piperazine-1-carboxylate **10** (5 g, 7.86 mmol) in DCM (21 mL), and the mixture was stirred at 50 °C overnight. The reaction mixture was then concentrated to dryness under reduced pressure, and the resultant crude material was purified by column chromatography to afford the title compound as white solid (3.3 g, yield 79%).^{1}H NMR (400 MHz, DMSO-d_6) δ 9.05 (s, 1H), 7.74 (dd, *J* = 8.0, 1.5 Hz, 1H), 7.63 (t, *J* = 8.5 Hz, 1H), 7.41 (d, *J* = 8.5 Hz, 1H), 7.31 – 7.21 (m, 3H), 7.21 – 7.13 (m, 4H), 7.00 (q, *J* = 9.1 Hz, 4H), 5.56 (t, *J* = 6.1 Hz, 1H), 4.28 (tt, *J* = 12.1, 3.7 Hz, 1H), 4.16 (d, *J* = 6.0 Hz, 2H), 3.65 – 3.53 (m, 1H), 3.11 (dd, *J* = 6.3, 3.7 Hz, 4H), 2.84 (t, *J* = 5.0 Hz, 4H), 1.96 (d, *J* = 10.9 Hz, 2H), 1.79 (d, *J* = 10.8 Hz, 2H), 1.41 (q, *J* = 13.1 Hz, 2H), 1.13 (q, *J* = 13.0 Hz, 2H).

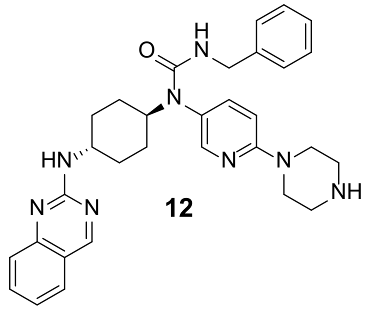

12

3-benzyl-1-(6-(piperazin-1-yl)pyridin-3-yl)-1-((1r,4r)-4-(quinazolin-2-ylamino) cyclohexyl)urea (12)

Compound **12** was synthesized by following a similar procedure as that of Compound **YJ9068**. ^{1}H NMR (400 MHz, DMSO-d_6) δ 9.05 (s, 1H), 7.94 (s, 1H), 7.75 (d, *J* = 8.0 Hz, 1H), 7.63 (t, *J* = 7.8 Hz, 1H), 7.39 (t, *J* = 8.5 Hz, 2H), 7.31 – 7.25 (m, 2H), 7.23 (d, *J* = 7.6 Hz, 1H), 7.21 – 7.13 (d, *J* = 7.4 Hz, 4H), 6.92 (d, *J* = 8.9 Hz, 1H), 6.04 (t, *J* = 6.2 Hz, 1H), 4.28 (t, *J* = 12.4 Hz, 1H), 4.16 (d, *J* = 5.9 Hz, 2H), 3.67 – 3.49 (m, 5H), 3.00 (t, *J* = 5.3 Hz, 4H), 1.98 (d, *J* = 12.3 Hz, 2H), 1.80 (d, *J* = 12.3 Hz, 2H), 1.42 (q, *J* = 12.4 Hz, 2H), 1.09 (q, *J* = 12.4 Hz, 2H).

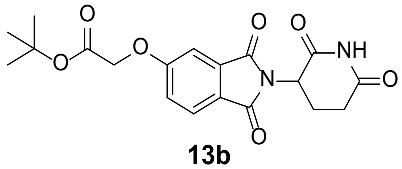

13b

tert-butyl 2-((2-(2,6-dioxopiperidin-3-yl)-1,3-dioxoisoindolin-5-yl)oxy)acetate (13b)

^{1}H NMR (400 MHz, DMSO-d_6) δ 11.11 (s, 1H), 7.81 (dd, *J* = 8.5, 7.3 Hz, 1H), 7.49 (d, *J* = 7.2 Hz, 1H), 7.38 (d, *J* = 8.6 Hz, 1H), 5.11 (dd, *J* = 12.9, 5.4 Hz, 1H), 4.97 (s, 2H), 2.96 – 2.83 (m, 1H), 2.65 – 2.53 (m, 2H), 2.10 – 1.99 (m, 1H), 1.43 (s, 9H).

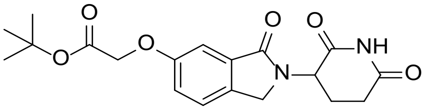

13c

tert-butyl 2-((2-(2,6-dioxopiperidin-3-yl)-3-oxoisoindolin-5-yl)oxy)acetate (13c)

^{1}H NMR (400 MHz, DMSO-d_6) δ 10.99 (s, 1H), 7.52 (d, *J* = 8.3 Hz, 1H), 7.21 (dd, *J* = 8.3, 2.5 Hz, 1H), 7.16 (d, *J* = 2.4 Hz, 1H), 5.10 (dd, *J* = 13.3, 5.1 Hz, 1H), 4.77 (s, 2H), 4.43 – 4.21 (m, 2H), 2.91 (ddd, *J* = 17.3, 13.6, 5.4 Hz, 1H), 2.66 – 2.56 (m, 1H), 2.39 (qd, *J* = 13.2, 4.4 Hz, 1H), 2.06 – 1.95 (m, 1H), 1.43 (s, 9H).

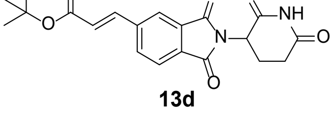

13d

tert-butyl (E)-3-(2-(2,6-dioxopiperidin-3-yl)-1,3-dioxoisoindolin-5-yl)acrylate (13d)

A mixture of 5-bromo-2-(2,6-dioxopiperidin-3-yl)isoindoline-1,3-dione (1 g, 2.95 mmol), *tert*-butyl acrylate (757.3 mg, 5.9 mmol), DIPEA (762.2 mg, 5.9 mmol), $Pd(AcO)_2$ (33.2 mg, 0.15 mmol), $P(ph)_3$ (77.5 mg, 0.3 mmol), and anhydrous DMF (40 mL) were added to a 100 mL round-bottom flask. The flask was evacuated and backfilled with argon (3 cycles). After stirring at 100 °C overnight, the mixture was then filtered and the solvent removed under vacuum. The crude material was purified by column chromatography to give **13d** as white solid (900 mg, yield 79%). ^{1}H NMR (400 MHz, DMSO-d_6) δ 11.14 (s, 1H), 8.30 (s, 1H), 8.19 (d, J = 7.8 Hz, 1H), 7.93 (d, J = 7.7 Hz, 1H), 7.74 (d, J = 16.1 Hz, 1H), 6.85 (d, J = 16.1 Hz, 1H), 5.17 (dd, J = 12.9, 5.4 Hz, 1H), 2.90 (ddd, J = 17.1, 13.8, 5.4 Hz, 1H), 2.65 – 2.52 (m, 2H), 2.08 (ddd, J = 12.6, 5.6, 3.3 Hz, 1H), 1.50 (s, 9H). MS (ESI), *m/z*: 382.8[M-H]$^-$.

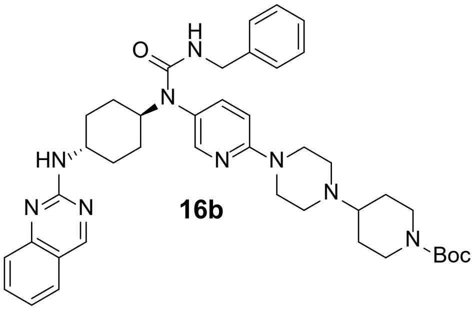

16b

tert-butyl 4-(4-(5-(3-benzyl-1-((1r,4r)-4-(quinazolin-2-ylamino)cyclohexyl) ureido)pyridin-2-yl)piperazin-1-yl)piperidine-1-carboxylate (16b)

To a solution of **13** (200 mg, 0.37 mmol) in DCM (20 mL) was added *tert*-butyl 4-oxopiperidine-1-carboxylate **15** (111 mg, 0.56 mmol). After stirring at room temperature for 30 minutes, then sodium triacetoxyborohydride (117.6 mg, 0.56 mmol) was added to the mixture. The reaction mixture was stirred for another 15 minutes then filtered, and the solvent was removed under reduced pressure. The crude material was purified by column chromatography to afford **16b** as white solid (138 mg, yield 52%).^{1}H NMR (400 MHz, DMSO-d_6) δ 9.05 (s, 1H), 7.91 (d, J = 2.6 Hz, 1H), 7.74 (d, J = 8.0 Hz, 1H), 7.63 (t, J = 7.9 Hz, 1H), 7.41 (d, J = 8.5 Hz, 1H), 7.37 – 7.31 (m, 1H), 7.26 (dt, J = 13.8, 7.8 Hz, 3H), 7.17 (d, J = 7.4 Hz, 4H), 6.88 (d, J = 9.0 Hz, 1H), 6.04 (t, J = 6.0 Hz, 1H), 4.29 (d, J = 12.0 Hz, 1H), 4.15 (d, J = 6.0 Hz, 2H), 3.60 (s, 2H), 3.52 (t, J = 5.2 Hz, 4H), 3.02 (d, J = 12.2 Hz, 2H), 2.58 (t, J = 5.1 Hz, 4H), 2.44 (dt, J = 14.3, 8.4 Hz, 3H), 2.32 (s, 1H), 1.97 (s, 2H), 1.84 – 1.69 (m, 4H), 1.51 – 1.33 (m, 11H), 1.29 – 1.21 (m, 2H),1.09 (q, J = 13.3 Hz, 2H).

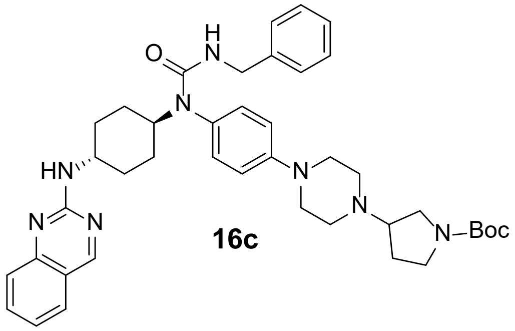

16c

tert-butyl 3-(4-(4-(3-benzyl-1-((1r,4r)-4-(quinazolin-2-ylamino)cyclohexyl)ureido)phenyl)piperazin-1-yl)pyrrolidine-1-carboxylate (16c)

Compound **16c** was synthesized by following a similar procedure as that of **16b**.^{1}H NMR (400 MHz, DMSO-d_6) δ 9.05 (s, 1H), 7.74 (d, J = 8.0 Hz, 1H), 7.63 (t, J = 7.7 Hz, 1H), 7.40 (d, J = 8.5 Hz, 1H), 7.31 – 7.25 (m, 2H), 7.22 (d, J = 7.8 Hz, 1H), 7.21 – 7.13 (m, 4H), 7.07 – 6.96 (m, 4H), 5.57 (t, J = 6.9 Hz, 1H), 4.28 (t, J = 11.4 Hz, 1H), 4.16 (d, J = 6.0 Hz, 2H), 3.65 – 3.45 (m, 3H), 3.41 (t, J = 9.7 Hz, 1H), 3.26 – 3.11 (m, 5H), 3.01 (q, J = 10.7 Hz, 1H), 2.88 – 2.73 (m, 1H), 2.65 – 2.56 (m, 2H), 2.12 – 1.89 (m, 4H), 1.74 – 1.62 (m, 1H), 1.72 (s, 1H), 1.50 – 1.32 (m, 11H), 1.13 (q, J = 13.3, 12.8 Hz, 2H).

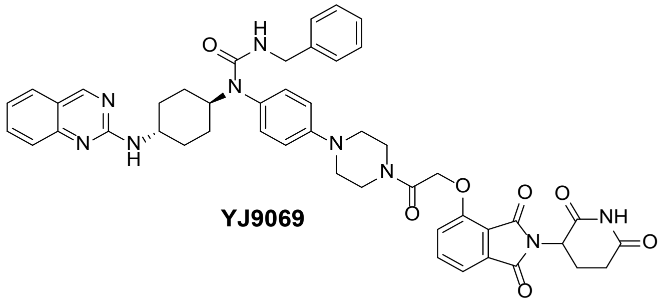

YJ9069

3-benzyl-1-(4-(4-(2-((2-(2,6-dioxopiperidin-3-yl)-1,3-dioxoisoindolin-4-yl)oxy) acetyl)piperazin-1-yl)phenyl)-1-((1r,4r)-4-(quinazolin-2-ylamino)cyclohexyl)urea (YJ9069)

To a solution of **YJ9068** (54 mg, 0.1 mmol), 2-((2-(2,6-dioxopiperidin-3-yl)-1,3-dioxoisoindolin-4-yl)oxy)acetic acid **13** (36.5 mg, 0.11), HATU (45.6 mg, 0.12 mmol), and DIPEA (25.8 mg, 0.2 mmol) in DMF (10 mL). The mixture was stirred at room temperature for 15 minutes and then evaporated under vacuum, purified by silica column chromatography to afford the compound **YJ9069** as a white solid (64 mg, yield 75%). ^{1}H NMR (400 MHz, DMSO-d_6) δ 11.12 (s, 1H), 9.05 (s, 1H), 7.79 (t, J = 7.9 Hz, 1H), 7.74 (d, J = 8.0 Hz, 1H), 7.63 (t, J = 7.8 Hz, 1H), 7.46 (d, J = 7.2 Hz, 1H), 7.39 (t, J = 8.1 Hz, 2H), 7.27 (q, J = 9.9, 8.7 Hz, 3H), 7.22 – 7.13 (m, 4H), 7.11 – 7.02 (m, 4H), 5.60 (t, J = 6.0 Hz, 1H), 5.26 (s, 2H), 5.12 (dd, J = 12.8, 5.4 Hz, 1H), 4.27 (d, J = 12.4 Hz, 1H), 4.17 (d, J = 6.0 Hz, 2H), 3.62 (s, 4H), 3.31 (s, 1H), 3.24 (s, 2H), 2.96 – 2.82 (m, 1H), 2.65 – 2.53 (m, 2H), 2.10 – 2.00 (m, 1H), 1.97 (d, J = 10.8 Hz, 2H), 1.80 (d, J = 11.8 Hz, 2H), 1.41 (q, J = 12.5 Hz, 2H), 1.13 (q, J = 12.3 Hz, 2H). ^{13}C NMR (151 MHz, DMSO-d_6) δ 173.31, 170.43, 167.29, 165.76, 165.61, 162.50, 157.33, 156.06, 150.34, 141.73, 137.09, 134.48, 133.55, 132.13 (2C), 129.17, 128.52 (3C), 128.32, 127.17 (4C), 126.75, 122.21, 120.67, 119.95, 116.60, 116.40 (2C), 116.00, 66.57, 53.54, 49.23, 49.13, 48.29, 48.16, 44.33, 43.94, 41.69, 40.043, 31.75, 31.40, 30.87 (2C), 22.46. HRMS (ESI) for $C_{47}H_{47}N_9O_7$[M+H]$^+$, calcd: 850.36712, found: 850.3646. HPLC analysis: MeOH-H_2O (80:20), 12.08 min, 97.4% purity.

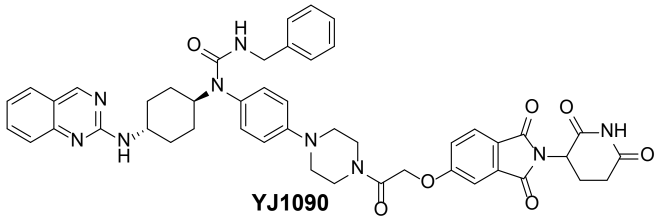

YJ1090

3-benzyl-1-(4-(4-(2-((2-(2,6-dioxopiperidin-3-yl)-1,3-dioxoisoindolin-5-yl)oxy)acetyl)piperazin-1-yl)phenyl)-1-((1r,4r)-4-(quinazolin-2-ylamino)cyclohexyl)urea (YJ1090)

Compound **YJ1090** was synthesized by following a similar procedure as that of **YJ9069**.^{1}H NMR (400 MHz, DMSO-d_6) δ 11.11 (s, 1H), 9.05 (s, 1H), 7.85 (d, J = 8.3 Hz, 1H), 7.74 (d, J = 8.3 Hz, 1H), 7.63 (t, J = 7.9 Hz, 1H), 7.48 (d, J = 2.3 Hz, 1H), 7.43 – 7.35 (m, 2H), 7.32 – 7.24 (m, 3H), 7.23 (d, J = 8.2 Hz, 1H), 7.21 – 7.13 (m, 4H), 7.11 – 7.00 (m, 4H), 5.60 (t, J = 5.8 Hz, 1H), 5.21 (s, 2H), 5.12 (dd, J = 12.9, 5.4 Hz, 1H), 4.27 (t, J = 12.3 Hz, 1H), 4.17 (d, J = 6.0 Hz, 2H), 3.70 – 3.52 (m, 5H), 3.31 (s, 2H), 3.24 (s, 2H), 2.95 – 2.83 (m, 1H), 2.65 – 2.54 (m, 2H), 2.10 – 2.01 (m, 1H), 1.97 (d, J = 11.4 Hz, 2H), 1.80 (d, J = 11.9 Hz, 2H), 1.41 (q, J = 12.4 Hz, 2H), 1.14 (q, J = 12.0 Hz, 2H). ^{13}C NMR (151 MHz, DMSO-d_6) δ 173.30, 170.42, 167.42, 167.27, 165.69, 164.15, 162.50, 157.32, 150.32, 141.74, 134.47, 134.17, 132.13 (2C), 129.19, 128.52 (3C), 128.32, 127.17 (4C), 126.74, 125.65, 123.70, 122.21, 121.69, 119.95, 116.39 (2C), 109.58, 66.60, 53.54, 49.45, 49.13, 48.34, 48.16, 44.28, 43.94, 41.64, 40.44, 31.75, 31.41, 30.87 (2C), 22.52. HRMS (ESI) for $C_{47}H_{47}N_9O_7$[M+H]$^+$, calcd: 850.36712, found: 850.3633. HPLC analysis: MeOH-H_2O (75:25), 6.96 min, 95.0% purity.

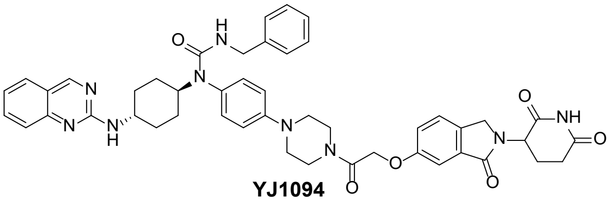

YJ1094

3-benzyl-1-(4-(4-(2-((2-(2,6-dioxopiperidin-3-yl)-3-oxoisoindolin-5-yl)oxy)acetyl)piperazin-1-yl)phenyl)-1-((1r,4r)-4-(quinazolin-2-ylamino)cyclohexyl)urea (YJ1094)

Compound **YJ1094** was synthesized by following a similar procedure as that of **YJ9069**.^{1}H NMR (400 MHz, DMSO-d_6) δ 10.98 (s, 1H), 9.05 (s, 1H), 7.74 (d, *J* = 7.9 Hz, 1H), 7.63 (t, *J* = 7.7 Hz, 1H), 7.51 (d, *J* = 8.2 Hz, 1H), 7.40 (d, *J* = 8.5 Hz, 1H), 7.31 – 7.21 (m, 5H), 7.21 – 7.13 (m, 4H), 7.08 – 7.00 (m, 4H), 5.60 (t, *J* = 5.9 Hz, 1H), 5.11 (dd, *J* = 13.2, 5.1 Hz, 1H), 5.03 (s, 2H), 4.43 – 4.21 (m, 3H), 4.16 (d, *J* = 6.0 Hz, 2H), 3.71 – 3.53 (m, 5H), 3.30 (s, 2H), 3.23 (s, 2H), 2.96 – 2.84 (m, 1H), 2.68 – 2.53 (m, 2H), 2.04 – 1.90 (s, 3H), 1.80 (d, *J* = 12.1 Hz, 2H), 1.41 (q, *J* = 12.3 Hz, 2H), 1.20 – 1.06 (m, 2H). ^{13}C NMR (151 MHz, DMSO-d_6) δ 173.41, 171.47, 168.57, 166.31, 162.50, 158.74, 157.33, 150.34, 141.75, 134.97, 134.48, 133.27, 132.11 (2C), 129.16, 128.52 (3C), 128.32, 127.16 (4C), 126.74, 124.92, 122.21, 120.65, 119.95, 116.38 (2C), 107.67, 66.52, 53.54, 52.21 (2C), 48.45, 48.18, 47.28, 44.44, 43.93, 41.63, 40.43, 31.74, 31.66, 30.86 (2C), 22.92. HRMS (ESI) for $C_{47}H_{49}N_9O_6$[M+H]$^+$, calcd: 836.38786, found: 836.3843. HPLC analysis: MeOH-H_2O (75:25), 6.33 min, 95.6% purity.

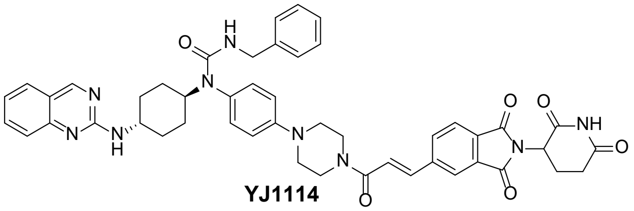

YJ1114

3-benzyl-1-(4-(4-((E)-3-(2-(2,6-dioxopiperidin-3-yl)-1,3-dioxoisoindolin-5-yl)acryloyl)piperazin-1-yl)phenyl)-1-((1r,4r)-4-(quinazolin-2-ylamino)cyclohexyl)urea (YJ1114)

Compound **YJ1114** was synthesized by following a similar procedure as that of **YJ9069**.^{1}H NMR (400 MHz, DMSO-d_6) δ 11.15 (s, 1H), 9.05 (s, 1H), 8.48 (s, 1H), 8.19 (d, *J* = 7.8 Hz, 1H), 7.96 (d, *J* = 7.7 Hz, 1H), 7.74 (d, *J* = 7.9 Hz, 1H), 7.71 (s, 2H), 7.63 (t, *J* = 7.8 Hz, 1H), 7.39 (d, *J* = 8.6 Hz, 1H), 7.31 – 7.21 (m, 3H), 7.21 – 7.13 (m, 4H), 7.07 (s, 4H), 5.59 (t, *J* = 6.4 Hz, 1H), 5.19 (dd, *J* = 13.0, 5.4 Hz, 1H), 4.27 (t, *J* = 11.9 Hz, 1H), 4.17 (d, *J* = 5.8 Hz, 2H), 3.95 (s, 2H), 3.76 (s, 2H), 3.58 (s, 1H), 2.97 – 2.83 (m, 1H), 2.69 – 2.56 (m, 2H), 2.13 – 2.04 (m, 2H), 1.96 (d, *J* = 11.9 Hz, 2H), 1.80 (d, *J* = 11.9 Hz, 2H), 1.41 (q, *J* = 12.1 Hz, 2H), 1.16 (q, *J* = 12.1 Hz, 2H). ^{13}C NMR (151 MHz, DMSO-d_6) δ 173.26, 170.31, 167.43, 167.23, 164.45, 162.49, 157.29, 150.37, 142.28, 141.79, 140.14, 135.37, 134.45, 132.59, 132.13 (2C), 131.66, 129.21, 128.51(3C), 128.31, 127.18(4C), 126.72, 124.38, 122.87, 122.63, 122.18, 119.95, 116.38 (2C), 53.54, 49.57 (2C), 48.90, 48.27, 45.38, 43.95, 42.19, 40.50, 31.76, 31.41, 30.88 (2C), 22.45. HRMS (ESI) for $C_{48}H_{47}N_9O_6$[M+H]$^+$, calcd: 846.37221, found: 846.3687. HPLC analysis: MeOH-H_2O (75:25), 14.00 min, 99.3% purity.

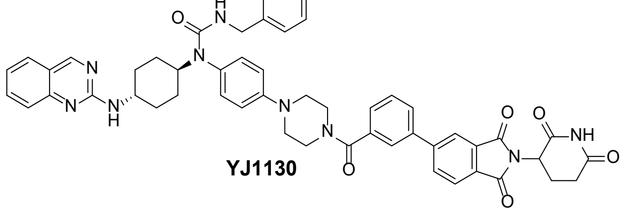

YJ1130

3-benzyl-1-(4-(4-(3-(2-(2,6-dioxopiperidin-3-yl)-1,3-dioxoisoindolin-5-yl)benzoyl)piperazin-1-yl)phenyl)-1-((1r,4r)-4-(quinazolin-2-ylamino)cyclohexyl)urea (YJ1130)

Compound **YJ1130** was synthesized by following a similar procedure as that of **YJ9069**.^{1}H NMR (400 MHz, DMSO-d_6) δ 11.15 (s, 1H), 9.05 (s, 1H), 8.30 – 8.21 (m, 2H), 8.03 (d, *J* = 7.7 Hz, 1H), 7.97 (d, *J* = 7.9 Hz, 1H), 7.92

(s, 1H), 7.75 (d, *J* = 8.1 Hz, 1H), 7.68 – 7.60 (m, 2H), 7.55 (d, *J* = 7.6 Hz, 1H), 7.40 (d, *J* = 8.5 Hz, 1H), 7.31 – 7.23 (m, 3H), 7.21 – 7.13 (m, 4H), 7.11 – 6.98 (m, 4H), 5.57 (t, *J* = 6.8 Hz, 1H), 5.19 (dd, *J* = 12.6, 5.3 Hz, 1H), 4.28 (t, *J* = 12.1 Hz, 1H), 4.16 (d, *J* = 5.7 Hz, 2H), 3.82 (s, 1H), 3.57 (s, 4H), 3.25 (s, 4H), 2.97 – 2.84 (m, 1H), 2.69 – 2.58 (m, 2H), 2.14 – 2.04 (m, 1H), 1.96 (d, *J* = 11.4 Hz, 2H), 1.80 (d, *J* = 12.0 Hz, 2H), 1.41 (q, *J* = 12.1 Hz, 2H), 1.14 (q, *J* = 12.1 Hz, 2H). ^{13}C NMR (151 MHz, DMSO-d_6) δ 173.26, 170.33, 169.02, 167.42, 167.39, 162.48, 157.27, 150.37, 146.31, 141.76, 138.78, 137.40, 134.44, 133.72, 132.81, 132.13 (2C), 130.67, 129.99, 129.25, 128.96, 128.51 (3C), 128.31, 127.93, 127.17 (4C), 126.72, 126.26, 124.58, 122.21, 122.18, 119.96, 116.46 (2C), 53.53, 49.57 (2C), 49.12, 48.28, 47.52, 43.95, 40.51, 31.75, 31.42 (2C), 30.88 (2C), 22.47. HRMS (ESI) for $C_{52}H_{49}N_9O_6[M+H]^+$, calcd: 896.38786, found: 896.3845. HPLC analysis: MeOH-H_2O (75:25), 19.82 min, 98.9% purity.

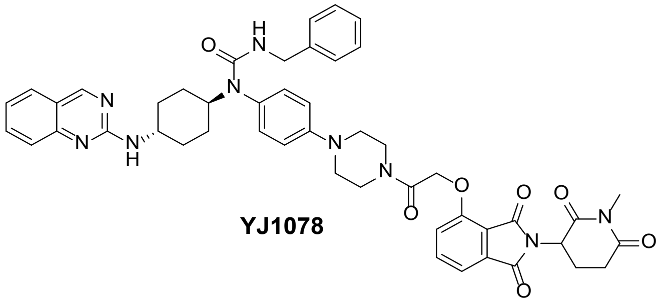

YJ1078

3-benzyl-1-(4-(4-(2-((2-(1-methyl-2,6-dioxopiperidin-3-yl)-1,3-dioxoisoindolin-4-yl)oxy)acetyl)piperazin-1-yl)phenyl)-1-((1r,4r)-4-(quinazolin-2-ylamino)cyclohexyl) urea (YJ1078)

Compound **YJ1078** was synthesized by following a similar procedure as that of **YJ9069**. ^{1}H NMR (400 MHz, DMSO-d_6) δ 9.05 (s, 1H), 7.79 (t, *J* = 7.8 Hz, 1H), 7.74 (d, *J* = 8.0 Hz, 1H), 7.63 (t, *J* = 7.8 Hz, 1H), 7.47 (d, *J* = 7.3 Hz, 1H), 7.39 (d, *J* = 8.4 Hz, 2H), 7.31 – 7.21 (m, 3H), 7.21 – 7.13 (m, 4H), 7.10 – 6.99 (s, 4H), 5.59 (t, *J* = 6.0 Hz, 1H), 5.25 (s, 2H), 5.18 (dd, *J* = 12.7, 5.0 Hz, 1H), 4.28 (t, *J* = 10.4 Hz, 1H), 4.17 (d, *J* = 6.0 Hz, 2H), 3.67 – 3.56 (m, 5H), 3.32 (s, 2H), 3.24 (s, 2H), 3.02 (s, 3H), 3.00 – 2.88 (m, 1H), 2.81 – 2.70 (m, 1H), 2.60 – 2.54 (m, 1H), 2.06 (d, *J* = 9.7 Hz, 1H), 1.97 (d, *J* = 11.6 Hz, 2H), 1.80 (d, *J* = 11.9 Hz, 2H), 1.42 (q, *J* = 12.8, 12.4 Hz, 2H), 1.14 (q, *J* = 12.3, 11.6 Hz, 2H). ^{13}C NMR (151 MHz, DMSO-d_6) δ 172.28, 170.18, 167.28, 165.76, 165.60, 162.50, 157.33, 156.07, 150.34, 141.72, 137.14, 134.48, 133.54, 132.12 (2C), 129.17, 128.52 (3C), 128.32, 127.17 (4C), 126.75, 122.21, 120.71, 119.95, 116.58, 116.39 (2C), 116.03, 66.61, 53.54, 49.81, 49.13, 48.31, 48.15, 44.34, 43.94, 41.70, 40.43, 31.75, 31.55, 30.87 (2C), 27.08, 21.67. HRMS (ESI) for $C_{48}H_{49}N_9O_7[M+H]^+$, calcd: 864.38277, found: 864.3804. HPLC analysis: MeOH-H_2O (75:25), 9.27 min, 97.5% purity.

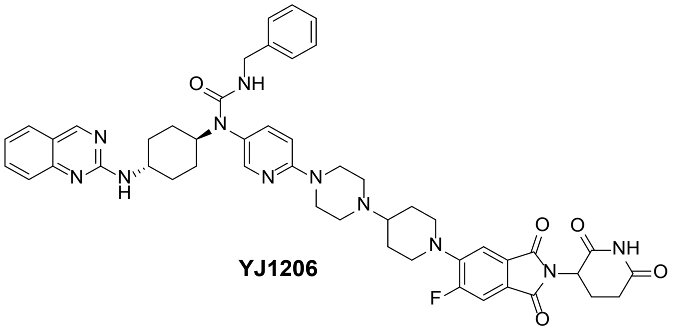

YJ1206

3-benzyl-1-(6-(4-(1-(2-(2,6-dioxopiperidin-3-yl)-6-fluoro-1,3-dioxoisoindolin-5-yl)piperidin-4-yl)piperazin-1-yl)pyridin-3-yl)-1-((1r,4r)-4-(quinazolin-2-ylamino) cyclohexyl)urea (YJ1206)

TFA (3 mL) was added to a suspension of **16b** (72 mg, 0.1 mmol) in DCM (6 mL). After stirring at room temperature for 3 hours, the reaction mixture was quenched with water and extracted with DCM three times. The combined organic phases were concentrated to dryness under reduced pressure. The resultant crude material was added to a suspension of compound **17b** (35 mg, 0.12 mmol) and DIPEA (39 mg, 0.3 mmol) in DMSO (10 mL). The resulting mixture was stirred at 120 °C for 8 h. The solvent was removed under vacuum to afford crude material which was purified by flash column chromatography to afford **YJ1206** as a yellow solid (56 mg, yield 63%). ^{1}H NMR (400 MHz, DMSO-d_6) δ 11.11 (s, 1H), 9.05 (s, 1H), 7.92 (s, 1H), 7.73 (t, *J* = 9.7 Hz, 2H), 7.63 (t, *J* = 8.0 Hz, 1H), 7.46 (d, *J* =

7.4 Hz, 1H), 7.41 (d, J = 8.5 Hz, 1H), 7.35 (d, J = 9.1 Hz, 1H), 7.31 – 7.25 (m, 2H), 7.21 (d, J = 7.5 Hz, 1H), 7.21 – 7.13 (m, 3H), 6.90 (d, J = 9.0 Hz, 1H), 6.05 (t, J = 5.4 Hz, 1H), 5.11 (dd, J = 13.0, 5.3 Hz, 1H), 4.28 (t, J = 12.3 Hz, 1H), 4.15 (d, J = 6.0 Hz, 2H), 3.68 (d, J = 11.9 Hz, 2H), 3.64 – 3.58 (m, 1H), 3.55(t, J = 4.5 Hz, 4H), 2.99 – 2.82 (m, 3H), 2.64 (s, 4H), 2.61 – 2.56 (m, 1H), 2.54(s, 2H), 2.08 – 1.87 (m, 5H), 1.79 (d, J = 11.7 Hz, 2H), 1.62 (q, J = 12.1, 11.3 Hz, 2H), 1.42 (q, J = 13.2, 12.5 Hz, 2H), 1.10 (q, J = 12.3 Hz, 2H). ^{13}C NMR (151 MHz, DMSO-d_6) δ 173.25, 170.40, 167.17, 166.69, 162.47, 158.33, 157.45, 156.89 (d, J = 253.4 Hz, 1C), 149.86, 146.00 (d, J = 8.49 Hz, 1C), 141.83, 140.60, 134.42, 129.26 (d, J = 2.2 Hz, 1C), 128.48 (3C), 128.30, 127.19 (4C), 126.69, 125.28, 124.42, 123.52 (d, J = 9.9 Hz, 1C), 122.17, 119.97, 114.24 (d, J = 4.5 Hz, 1C), 112.48 (d, J = 25.5 Hz, 1C), 107.40, 60.96, 55.39, 53.50, 49.84 (2C), 49.52 (2C), 49.11 (2C), 45.30, 43.97, 40.51, 31.72, 31.42, 30.85 (2C), 28.32 (2C), 22.55. HRMS (ESI) for $C_{49}H_{52}N_{11}O_5F[M+H]^+$, calcd: 894.42097, found: 894.4176. HPLC analysis: MeOH-H_2O (80:20), 8.79 min, 98.9% purity.

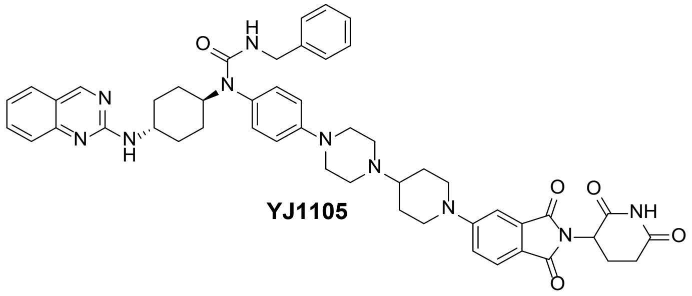

YJ1105

3-benzyl-1-(4-(4-(1-(2-(2,6-dioxopiperidin-3-yl)-1,3-dioxoisoindolin-5-yl)piperidin-4-yl)piperazin-1-yl)phenyl)-1-((1r,4r)-4-(quinazolin-2-ylamino)cyclohexyl)urea (YJ1105)

Compound **YJ1105** was synthesized by following a similar procedure as that of **YJ1206**.^{1}H NMR (400 MHz, DMSO-d_6) δ 11.08 (s, 1H), 9.05 (s, 1H), 7.74 (d, J = 8.0 Hz, 1H), 7.67 (d, J = 8.5 Hz, 1H), 7.63 (t, J = 7.5 Hz, 2H), 7.40 (d, J = 8.5 Hz, 1H), 7.34 (d, J = 2.2 Hz, 1H), 7.30 – 7.24 (m, 3H), 7.23 (d, J = 8.1 Hz, 1H), 7.20 – 7.12 (m, 4H), 7.01 (q, J = 8.8 Hz, 4H), 5.57 (t, J = 6.0 Hz, 1H), 5.07 (dd, J = 12.9, 5.4 Hz, 1H), 4.26 (t, J = 12.1 Hz, 1H), 4.16 (d, J = 6.0 Hz, 2H), 4.09 (d, J = 10.1 Hz, 2H), 3.65 – 3.53 (m, 1H), 3.26 – 3.12 (m, 4H), 3.00 (t, J = 12.3 Hz, 2H), 2.94 – 2.82 (m, 1H), 2.65 (t, J = 4.9 Hz, 4H), 2.62 – 2.59 (m, 1H), 2.59 – 2.53 (m, 2H), 2.07 – 1.86 (m, 5H), 1.78 (d, J = 11.6 Hz, 2H), 1.50 (q, J = 11.1 Hz, 2H), 1.41 (q, J = 12.5 Hz, 4H), 1.13 (q, J = 12.5, 11.9 Hz, 2H). ^{13}C NMR (151 MHz, DMSO-d_6) δ 173.37, 170.61, 168.11, 167.48, 162.50, 157.39, 155.22, 150.62, 141.70, 134.48, 131.99 (2C), 128.56, 128.52 (3C), 128.32, 127.14 (4C), 126.75, 125.53, 125.24, 122.23, 119.94, 118.17, 118.01, 115.79 (2C), 108.24, 60.99, 53.51, 49.20 (3C), 48.38 (2C), 47.06 (2C), 43.91, 40.73, 40.36, 31.75, 31.42, 30.85 (2C), 27.64 (2C), 22.65. HRMS (ESI) for $C_{50}H_{54}N_{10}O_5[M+H]^+$, calcd: 875.43514, found: 875.4322. HPLC analysis: MeOH-H_2O (80:20), 11.36 min, 99.2% purity.

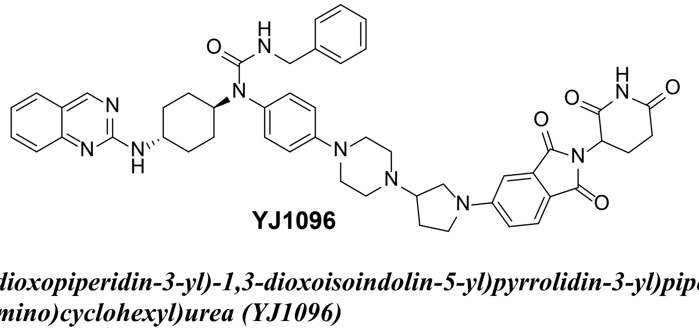

YJ1096

3-benzyl-1-(4-(4-(1-(2-(2,6-dioxopiperidin-3-yl)-1,3-dioxoisoindolin-5-yl)pyrrolidin-3-yl)piperazin-1-yl)phenyl)-1-((1r,4r)-4-(quinazolin-2-ylamino)cyclohexyl)urea (YJ1096)

Compound **YJ1096** was synthesized by following a similar procedure as that of **YJ1206**.^{1}H NMR (400 MHz, DMSO-d_6) δ 11.07 (s, 1H), 9.05 (s, 1H), 7.74 (d, J = 8.0 Hz, 1H), 7.68 – 7.59 (m, 2H), 7.40 (d, J = 8.4 Hz, 1H), 7.28 (t, J = 7.5 Hz, 2H), 7.23 (d, J = 8.1 Hz, 1H), 7.18 (t, J = 7.3 Hz, 4H), 7.04 (s, 4H), 6.99 (s, 1H), 6.87 (d, J = 9.0 Hz, 1H), 5.58 (d, J = 7.0 Hz, 1H), 5.06 (dd, J = 12.7, 5.4 Hz, 1H), 4.30 (t, J = 12.4 Hz, 1H), 4.16 (d, J = 5.9 Hz, 2H), 3.75 (t, J = 8.4 Hz, 1H), 3.66 – 3.53 (m, 2H), 3.42 (q, J = 8.8 Hz, 2H), 3.31 – 3.27 (m, 1H), 3.24 (t, J = 6.2 Hz, 4H), 3.02 (p, J = 7.6 Hz, 1H), 2.95 – 2.82 (m, 1H), 2.67 (s, 4H), 2.63 – 2.53 (m, 2H), 2.34 – 2.26 (m, 1H), 2.05 – 1.88 (m, 4H), 1.79 (d, J = 11.7 Hz, 2H), 1.41 (q, J = 12.4 Hz, 2H), 1.13 (q, J = 12.8 Hz, 2H). ^{13}C NMR (151 MHz, DMSO-d_6) δ

173.35, 170.65, 168.20, 167.74, 162.49, 157.36, 152.27, 150.57, 141.76, 134.46, 132.03 (2C), 128.74, 128.52 (4C), 128.31, 127.15 (4C), 126.74, 125.41, 122.21, 119.95, 116.22, 115.92 (2C), 115.71, 106.05, 63.97, 53.52, 52.42, 51.76 (2C), 49.15 (2C), 48.05 (2C), 47.49, 43.93, 40.43, 31.76, 31.45, 30.87 (2C), 29.28, 22.71. HRMS (ESI) for $C_{49}H_{52}N_{10}O_5[M+H]^+$, calcd: 861.41949, found: 861.4168. HPLC analysis: MeOH-H_2O (75:25), 15.48 min, 98.2% purity.

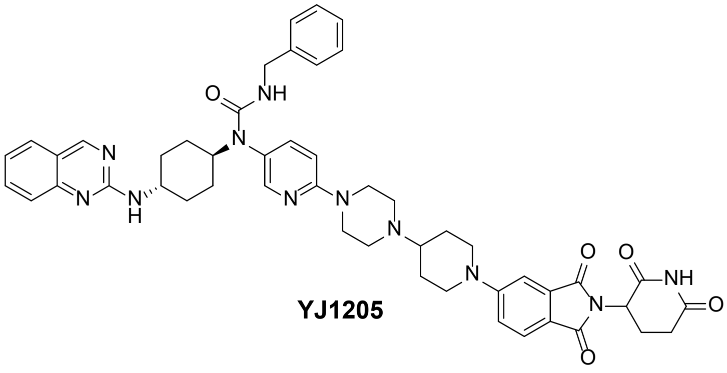

YJ1205

3-benzyl-1-(6-(4-(1-(2-(2,6-dioxopiperidin-3-yl)-1,3-dioxoisoindolin-5-yl)piperidin-4-yl)piperazin-1-yl)pyridin-3-yl)-1-((1r,4r)-4-(quinazolin-2-ylamino)cyclohexyl)urea (YJZ1205)

Compound **YJ1205** was synthesized by following a similar procedure as that of **YJ1206**. ^1H NMR (400 MHz, DMSO-d_6) δ 11.08 (s, 1H), 9.05 (s, 1H), 7.91 (s, 1H), 7.74 (d, J = 8.0 Hz, 1H), 7.70 – 7.58 (m, 2H), 7.40 (d, J = 8.4 Hz, 1H), 7.34 (s, 2H), 7.31 – 7.21 (m, 4H), 7.21 – 7.13 (m, 4H), 6.88 (d, J = 9.0 Hz, 1H), 6.05 (t, J = 6.6 Hz, 1H), 5.07 (dd, J = 12.8, 5.3 Hz, 1H), 4.26 (t, J = 12.8 Hz, 1H), 4.12 (dd, J = 18.7, 9.2 Hz, 4H), 3.60 (s, 1H), 3.52 (s, 4H), 3.00 (t, J = 12.4 Hz, 2H), 2.94 – 2.81 (m, 1H), 2.70 – 2.53 (m, 7H), 2.07 – 1.93 (m, 3H), 1.90 (d, J = 12.2 Hz, 2H), 1.78 (d, J = 11.7 Hz, 2H), 1.57 – 1.33 (m, 4H), 1.09 (q, J = 12.9 Hz, 2H). ^{13}C NMR (151 MHz, DMSO-d_6) δ 173.30, 170.59, 168.09, 167.44, 162.47, 158.32, 157.45, 155.22, 149.85, 141.82, 140.60, 134.51, 134.43, 128.48 (3C), 128.31, 127.18 (4C), 126.68, 125.49, 124.43, 122.17, 119.97, 118.17, 118.09, 108.28, 107.40, 61.12, 55.39, 53.49, 49.21(2C), 49.10 (2C), 47.10 (2C), 45.29, 43.96, 31.70, 31.45, 30.85 (2C), 27.66 (2C), 22.66. HRMS (ESI) for $C_{49}H_{53}N_{11}O_5[M+H]^+$, calcd: 876.43039, found: 876.4276. HPLC analysis: MeOH-H_2O (80:20), 6.94 min, 97.8% purity.

The ^{1}H NMR, ^{13}C NMR, HRMS, and HPLC traces of compounds YJ9069, YJ1078, and YJ1206.

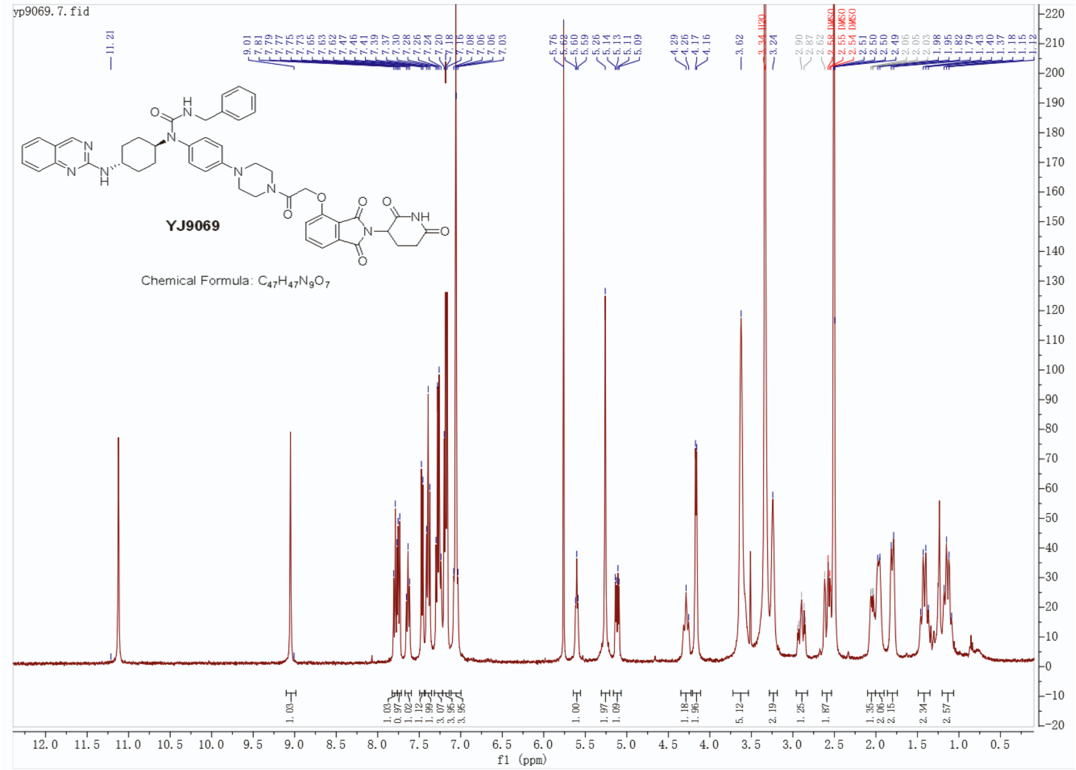

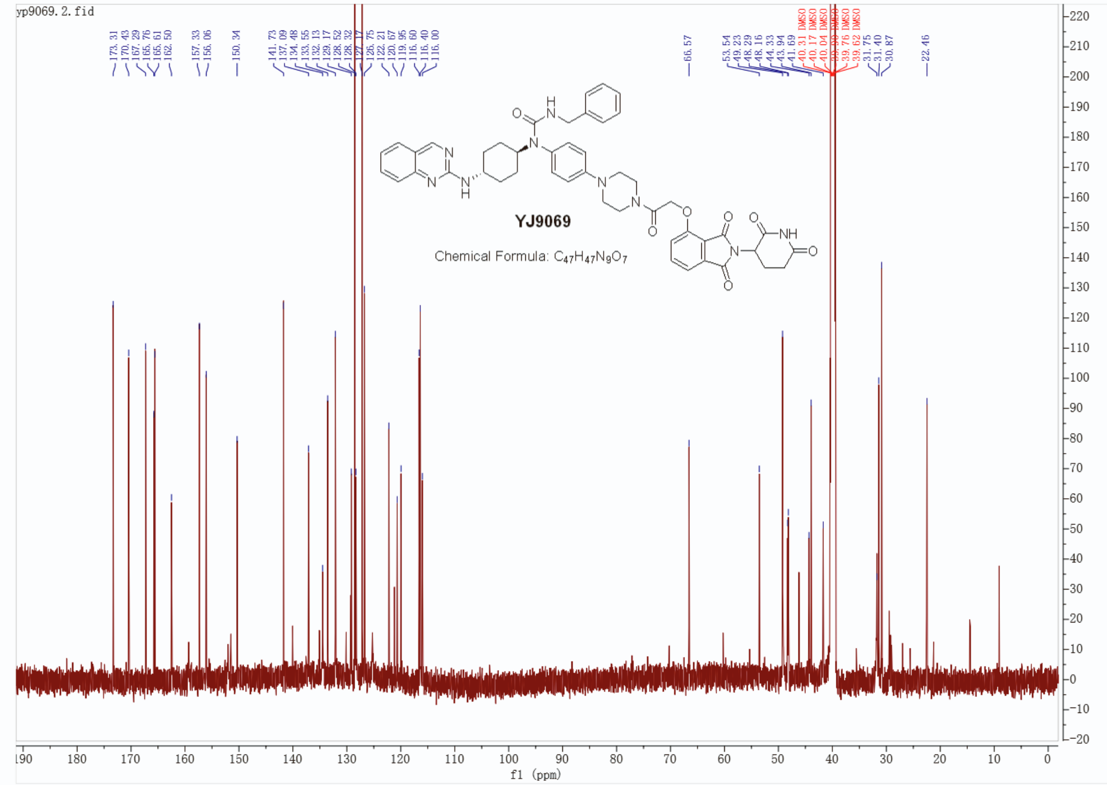

Data File E:\DK\YJZ\data\20210904\4996.D
Sample Name: 9069

===

Acq. Operator : 系统
Sample Operator : 系统
Acq. Instrument : 1260LC Location : 11
Injection Date : 04/09/2021 14:49:45
Inj Volume : 10.000 µl
Method : E:\DK\TL\方法\70C-30D-30min-1u.M
Last changed : 04/09/2021 14:22:01 by 系统
(modified after loading)

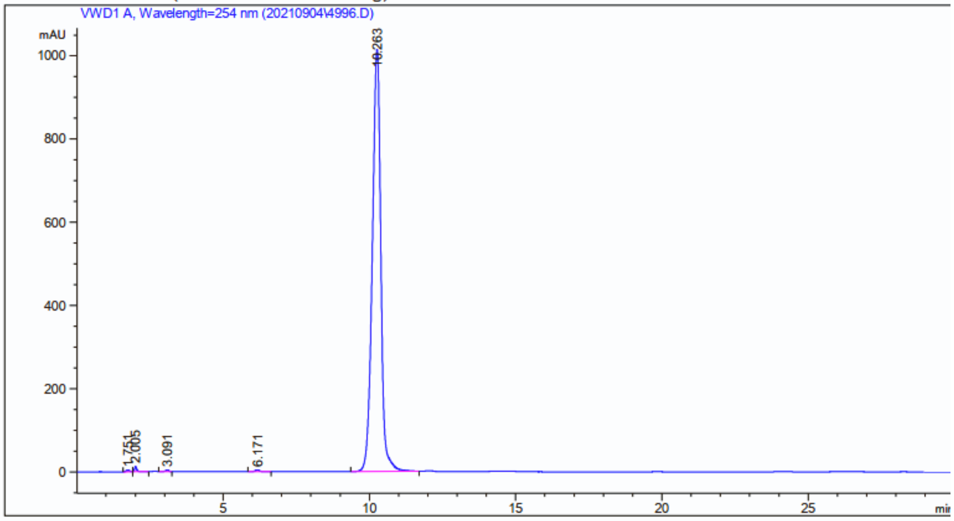

===

Area Percent Report

===

Sorted By : Signal
Multiplier : 1.0000
Dilution : 1.0000
Sample Amount: : 10.00000 [ng/ul] (not used in calc.)
Use Multiplier & Dilution Factor with ISTDs

Signal 1: VWD1 A, Wavelength=254 nm

Peak #	RetTime [min]	Type	Width [min]	Area [mAU*s]	Height [mAU]	Area %
1	1.751	BV	0.1065	36.82307	4.80255	0.1776
2	2.005	VB	0.0835	69.05422	12.48212	0.3330
3	3.091	VB	0.1160	29.43379	3.77336	0.1419
4	6.171	BB	0.1584	44.70434	4.22628	0.2156
5	10.263	BB	0.3102	2.05592e4	1015.45947	99.1320

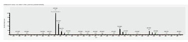

Elemental Composition Results - Spectrum 1

Peak Mass	Display Formula	S Fit	RDB	Delta [ppm]	Theo. mass	Rank	Combined Score	# Matched Iso.	# Missed Iso.	MS Cov. [%]	Pattern Cov. [%]
850.3646	$C_{46}H_{52}O_{11}N_5$	68.80	23.50	-1.36	850.36578	1	98.36	5	0	100.00	100.00
850.3646	$C_{45}H_{56}O_{15}N$	65.54	18.50	0.21	850.36445	2	98.19	5	0	100.00	100.00
850.3646	$C_{47}H_{48}O_7N_9$	59.19	28.50	-2.94	850.36712	3	97.85	5	0	100.00	100.00
850.3646	$C_{58}H_{48}O_4N_3$	28.76	36.50	0.81	850.36393	4	94.19	5	0	97.82	97.12
850.3646	$C_{41}H_{52}O_{13}N_7$	24.88	19.50	3.37	850.36176	5	85.54	5	0	88.91	89.93

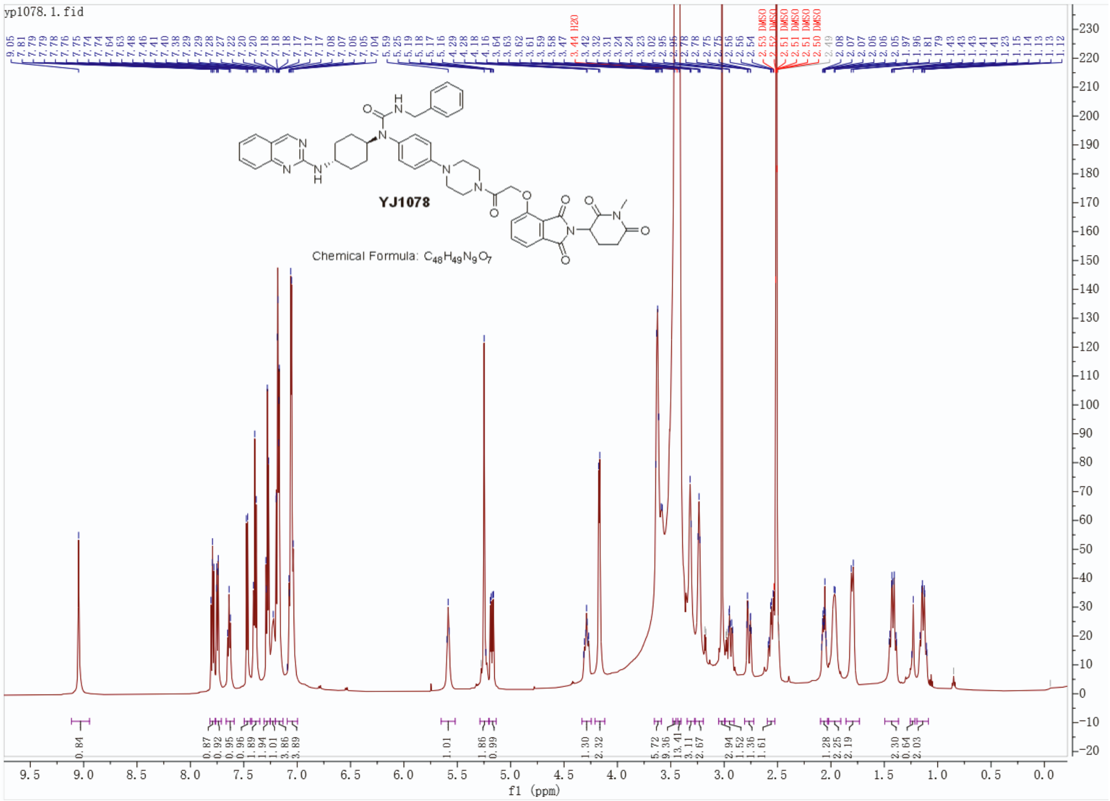
yp1078.1.fid
YJ1078
Chemical Formula: $C_{48}H_{49}N_9O_7$
f1 (ppm)

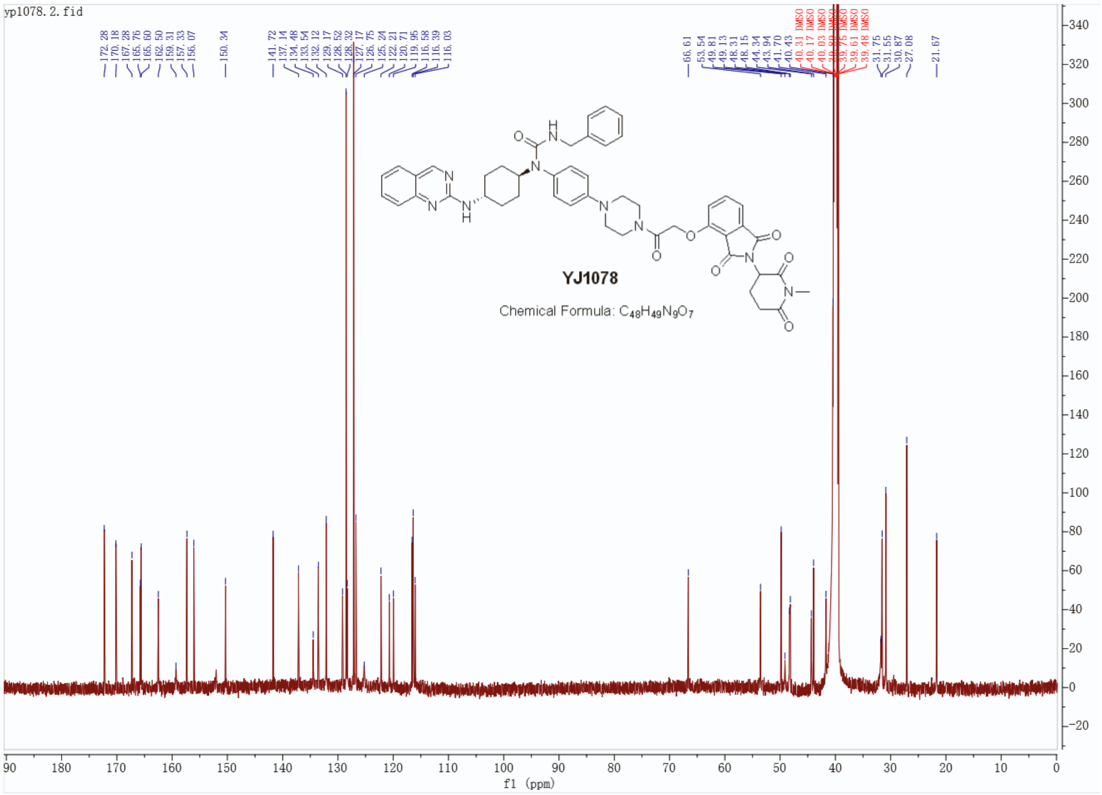
yp1078.2.fid
YJ1078
Chemical Formula: $C_{48}H_{49}N_9O_7$
f1 (ppm)

Data File E:\DK\YJZ\data\20221017\YJZ1901DEF_LC 2022-10-17 21-09-45\008-88-1078.D
Sample Name: 1078

===

Acq. Operator : 系统 Seq. Line : 8
Acq. Instrument : 1260LC Location : 88
Injection Date : 18/10/2022 00:53:15 Inj : 1
Inj Volume : 5.000 µl
Different Inj Volume from Sample Entry! Actual Inj Volume : 10.000 µl
Method : E:\DK\YJZ\data\20221017\YJZ1901DEF_LC 2022-10-17 21-09-45\75C-25D-30min-Y.M
(Sequence Method)
Last changed : 17/10/2022 21:09:45 by 系统

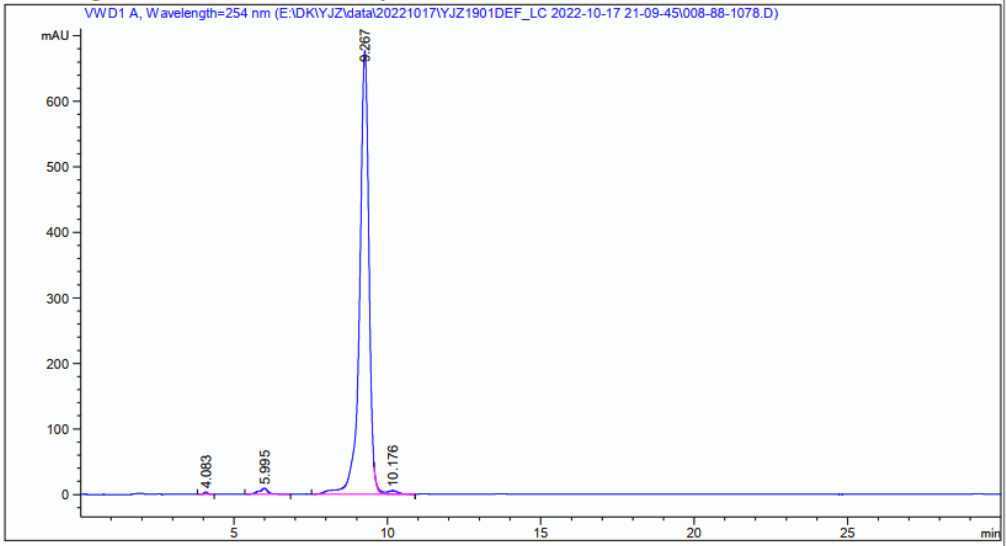

===

Area Percent Report

===

Sorted By : Signal
Multiplier : 1.0000
Dilution : 1.0000
Use Multiplier & Dilution Factor with ISTDs

Signal 1: VWD1 A, Wavelength=254 nm

Peak #	RetTime [min]	Type	Width [min]	Area [mAU*s]	Height [mAU]	Area %
1	4.083	BB	0.1157	27.69697	3.64108	0.1890
2	5.995	BB	0.2804	186.65962	9.25254	1.2735
3	9.267	BV R	0.3182	1.42854e4	676.94824	97.4599
4	10.176	VB E	0.4224	157.96930	5.28968	1.0777

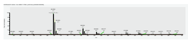

Elemental Composition Results - Spectrum 1

Peak Mass	Display Formula	S Fit	RDB	Delta [ppm]	Theo. mass	Rank	Combined Score	# Matched Iso.	# Missed Iso.	MS Cov. [%]	Pattern Cov. [%]
864.3804	$C_{47}H_{54}O_{11}N_5$	65.96	23.50	-1.16	864.38143	1	98.15	5	0	99.94	100.00
864.3804	$C_{48}H_{50}O_7N_9$	59.89	28.50	-2.71	864.38277	2	97.83	5	0	99.94	100.00
864.3804	$C_{46}H_{58}O_{15}N$	54.23	18.50	0.39	864.38010	3	97.34	6	0	99.74	99.61
864.3804	$C_{42}H_{54}O_{13}N_7$	32.12	19.50	3.50	864.37741	4	96.12	5	0	99.68	99.69
864.3804	$C_{59}H_{50}O_4N_3$	35.04	36.50	0.98	864.37958	5	94.71	6	0	98.03	97.00

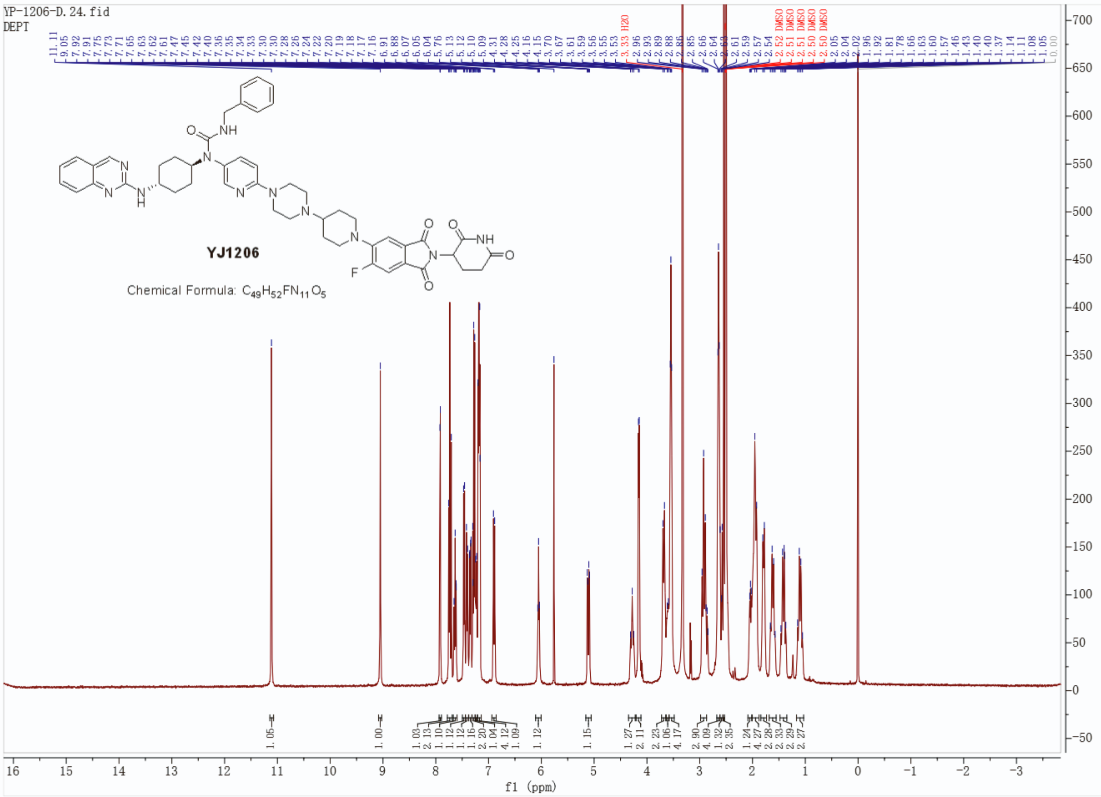
YP-1206-D.24.fid
DEPT
YJ1206
Chemical Formula: $C_{49}H_{52}FN_{11}O_5$
f1 (ppm)

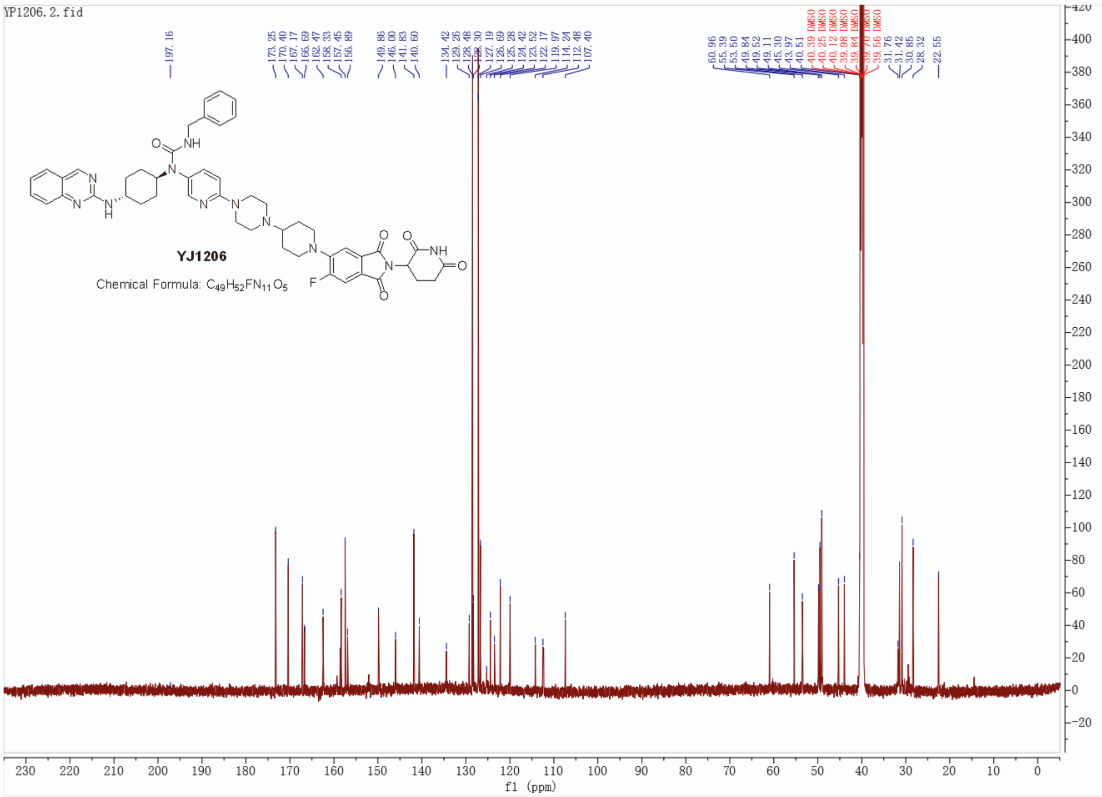
YP1206.2.fid
YJ1206
Chemical Formula: $C_{49}H_{52}FN_{11}O_5$
f1 (ppm)

Data File E:\DK\YJZ\data\20221019\YJZ1901DEF_LC 2022-10-19 17-10-43\004-77-1206.D
Sample Name: 1206

===

Acq. Operator : 系统 Seq. Line : 4
Acq. Instrument : 1260LC Location : 77
Injection Date : 19/10/2022 18:14:13 Inj : 1
Inj Volume : 5.000 µl
Different Inj Volume from Sample Entry! Actual Inj Volume : 10.000 µl
Method : E:\DK\YJZ\data\20221019\YJZ1901DEF_LC 2022-10-19 17-10-43\80C-20D-20MIN-20UL.M (Sequence Method)
Last changed : 19/10/2022 17:10:43 by 系统

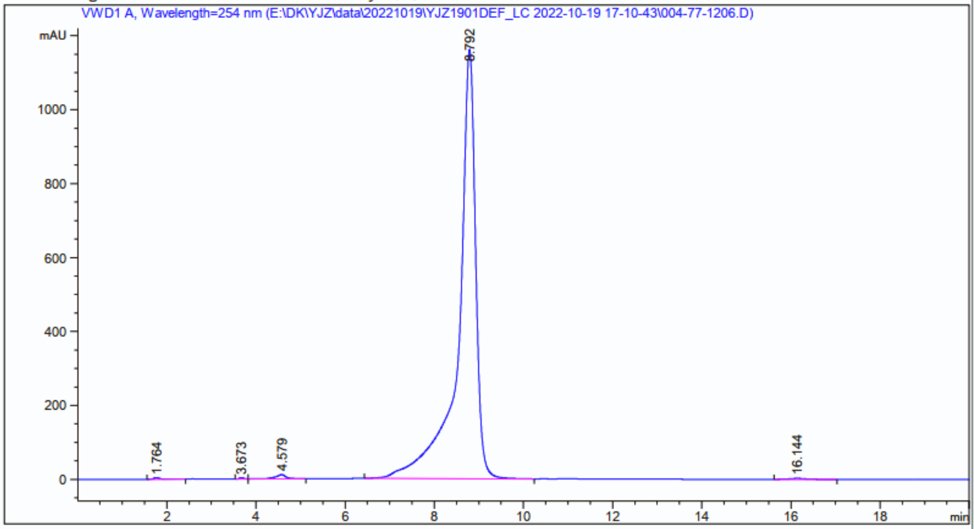

===

Area Percent Report

===

Sorted By : Signal
Multiplier : 1.0000
Dilution : 1.0000
Use Multiplier & Dilution Factor with ISTDs

Signal 1: VWD1 A, Wavelength=254 nm

Peak #	RetTime [min]	Type	Width [min]	Area [mAU*s]	Height [mAU]	Area %
1	1.764	BV R	0.1694	51.99037	4.20069	0.1647
2	3.673	BV	0.1049	27.78824	3.95838	0.0880
3	4.579	VB R	0.2552	219.13762	11.82306	0.6941
4	8.792	BB	0.3837	3.12137e4	1160.87976	98.8703
5	16.144	BB	0.3604	57.72878	2.45443	0.1829

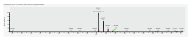

Elemental Composition Results - Spectrum 1

Peak Mass	Display Formula	S Fit	RDB	Delta [ppm]	Theo. mass	Rank	Combined Score	# Matched Iso.	# Missed Iso.	MS Cov. [%]	Pattern Cov. [%]
894.4176	$C_{52}H_{52}O_4N_{11}$	66.86	32.50	-2.49	894.41983	5	98.26	5	0	100.00	100.00
894.4176	$C_{44}H_{62}O_{14}N_3F_2$	62.02	14.50	-2.05	894.41944	6	98.00	5	0	100.00	100.00
894.4176	$C_{48}H_{48}O_2N_{17}$	60.97	33.50	0.52	894.41714	7	97.95	5	0	100.00	100.00
894.4176	$C_{57}H_{54}O_3N_5F_2$	59.22	32.50	-1.48	894.41892	8	97.85	5	0	100.00	100.00
894.4176	$C_{47}H_{52}O_6N_{13}$	53.94	28.50	2.01	894.41580	9	97.58	5	0	100.00	100.00
894.4176	$C_{49}H_{53}O_5N_{11}F$	52.01	28.50	-3.76	894.42097	10	97.47	5	0	100.00	100.00
